# Impact of the COVID‐19 pandemic on cancer healthcare utilization in southwestern China on March 2021

**DOI:** 10.1002/cam4.6028

**Published:** 2023-05-11

**Authors:** Peiyi Li, Yajuan Zhu, Yaqiang Wang, Xiaoyu Liu, Xiang Fang, Yuanxin Hou, Rujun Zheng, Junying Li, Bo Zhang, Zhuo Chen, Chengdi Wang, Tao Zhu, Weimin Li, Xuesong Han

**Affiliations:** ^1^ Department of Anesthesiology West China Hospital, Sichuan University Chengdu Sichuan China; ^2^ Laboratory of Anesthesia and Critical Care Medicine, National‐Local Joint Engineering Research Centre of Translational Medicine of Anesthesiology West China Hospital, Sichuan University Chengdu Sichuan China; ^3^ The Research Units of West China (2018RU012)‐Chinese Academy of Medical Sciences West China Hospital, Sichuan University Chengdu Sichuan China; ^4^ Department of Biotherapy and Cancer Center, State Key Laboratory of Biotherapy West China Hospital, Sichuan University Chengdu Sichuan China; ^5^ College of Software Engineering, Chengdu University of Information Technology Chengdu Sichuan China; ^6^ Sichuan Key Laboratory of Software Automatic Generation and Intelligent Service Chengdu Sichuan China; ^7^ Department of Health Policy and Management UCLA Fielding School of Public Health, University of California Los Angeles California USA; ^8^ Department of Orthopedics Orthopedic Research Institute, West China Hospital, Sichuan University Chengdu Sichuan China; ^9^ Institute of Hospital Management, West China Hospital of Sichuan University Chengdu China; ^10^ Department of Thoracic Oncology West China School of Nursing, West China Hospital, Sichuan University Chengdu Sichuan China; ^11^ Department of Neurology and ICCTR Biostatistics and Research Design Center Boston Children's Hospital, Harvard Medical School Boston Massachusetts USA; ^12^ Department of Health Policy and Management College of Public Health, University of Georgia Athens Georgia USA; ^13^ Faculty of Humanities and Social Sciences School of Economics, University of Nottingham Ningbo China Ningbo Zhejiang China; ^14^ Department of Respiratory and Critical Care Medicine West China Hospital, Sichuan University Chengdu Sichuan China; ^15^ Frontiers Science Center for Disease‐Related Molecular Network Institute of Respiratory Health, West China Hospital, Sichuan University Chengdu Sichuan China; ^16^ President's Office West China Hospital, Sichuan University Chengdu Sichuan China; ^17^ Surveillance & Health Equity Science, American Cancer Society Atlanta Georgia USA

**Keywords:** cancer care disparities, cancer care utilization, COVID‐19, southwestern China

## Abstract

**Background:**

Oncological care has been disrupted worldwide during the COVID‐19 pandemic. We aimed to quantify the long‐term impact of the pandemic on cancer care utilization and to examine how this impact varied by sociodemographic and clinical factors in southwestern China, where the Dynamic Zero‐COVID Strategy was implemented. This strategy mainly included lockdowns, stringent testing, and travel restrictions to prevent the spread of COVID‐19.

**Method:**

We identified 859,497 episodes of the utilization of cancer care from electronic medical records between January 1, 2019, and March 31, 2021, from the cancer center of a tertiary hospital serving an estimated population of 8.4 million in southwestern China. Changes in weekly utilization were evaluated via segmented Poisson regression across service categories, stratified by cancer type and sociodemographic factors.

**Results:**

A sharp reduction in utilization of in‐person cancer services occurred during the first week of the pandemic outbreak in January 2020, followed by a quick rebound in February 2020. Although there were few COVID‐19 cases from March 2020 until this analysis, the recovery of most in‐person services was slow and remained incomplete as of March 31, 2021. The exceptions were outpatient radiation and surgery, which increased and exceeded pre‐pandemic levels, particularly among lung cancer patients; meanwhile, telemedicine utilization increased substantially after the onset of the pandemic. Care disruptions were most prominent for women, rural residents, uninsured, and breast cancer patients.

**Conclusions:**

As of March 2021, despite few COVID‐19 cases, the COVID‐19 pandemic has had a strong and continuing impact on in‐person oncology care utilization in southwestern China under the Dynamic Zero‐COVID Strategy. Equitable and timely access to cancer care requires adjustment in strict policies for COVID‐19 prevention and control, as well as targeted remedies for the most vulnerable populations during and beyond the pandemic. Future studies should monitor the long‐term effects of the COVID‐19 pandemic and response strategies on cancer care and outcomes.

## Abstract 2

The COVID‐19 pandemic's disruptive impacts on oncology care may persist under the “Dynamic Zero‐COVID Strategy” in Southwestern China, with preserved healthcare capacity but overwhelming testing requirements and travel restrictions. Balanced and coordinated efforts are urgently needed to ensure vulnerable patients' equitable and timely access to affordable oncology care.

**What's new**

- As of March 31, 2021, utilization of most in‐person cancer care services in southwestern China had not returned to pre‐pandemic levels, except for outpatient radiation and surgery services, particularly for lung cancer patients, as well as telemedicine use.
- Women, rural residents, uninsured patients, and breast cancer patients experienced the most care disruptions during the pandemic. The COVID‐19 pandemic's disruptive impact on oncology care may endure under the Dynamic Zero‐COVID Strategy in southwestern China, with preserved healthcare capacity but overwhelming testing requirements and travel restrictions.
- Balanced and coordinated efforts are urgently needed to ensure vulnerable patients' equitable and timely access to affordable oncology care.

## 1 INTRODUCTION

Responses to the COVID‐19 pandemic have varied by country.^1^ China's Dynamic Zero‐COVID Strategy, carried out nationwide throughout the pandemic, involves large‐scale community lockdowns and travel bans, and mass testing once a case is detected.^2^ Nonpharmaceutical interventions (NPIs) had early success in reducing COVID‐19 infections and deaths following the outbreak in 2020, with only 87,052 infections and 4634 deaths from COVID‐19 nationwide,^3^ but also with extensive mobility restrictions across China.^4^ For instance, following Wuhan's lockdown on January 23, 2020, over 95% of Chinese cities had implemented infection control measures by February 4, 2020; approximately 40% of cities suspended intracity public transportation (bus and subway), and 64% limited travel to and from any other cities.^5^, ^6^ China maintained this Dynamic Zero‐COVID Strategy nationwide for almost 2 years, until relaxations began in December 2022.

Disruptions in cancer care have been reported worldwide during the pandemic owing to overwhelmed healthcare systems, mobility restrictions caused by regional lockdowns, fear of COVID‐19 infection, and potential individual wage loss.^7^, ^8^ However, how cancer care was affected in China under the Dynamic Zero‐COVID Strategy remains largely unknown. Cancer is the leading cause of death in China.^9^ Each year 24% of the world's newly diagnosed cancer cases and 30% of its cancer deaths occur in China, with the highest cancer incidence rates in southwestern China.^10^, ^11^ In Sichuan, the most populous province in southwestern China, it is estimated that 209,175 new cancer cases were diagnosed in 2018.^12^ To inform COVID prevention and control efforts and oncology response practices, we examined changes in cancer care utilization following the COVID‐19 pandemic by service type, and identified populations most affected by the pandemic, using medical record data from a large hospital in southwestern China during the period January 1, 2019, through March 31, 2021.

## 2 METHODS

### 2.1 Study setting and data sources

**FIGURE 1:**
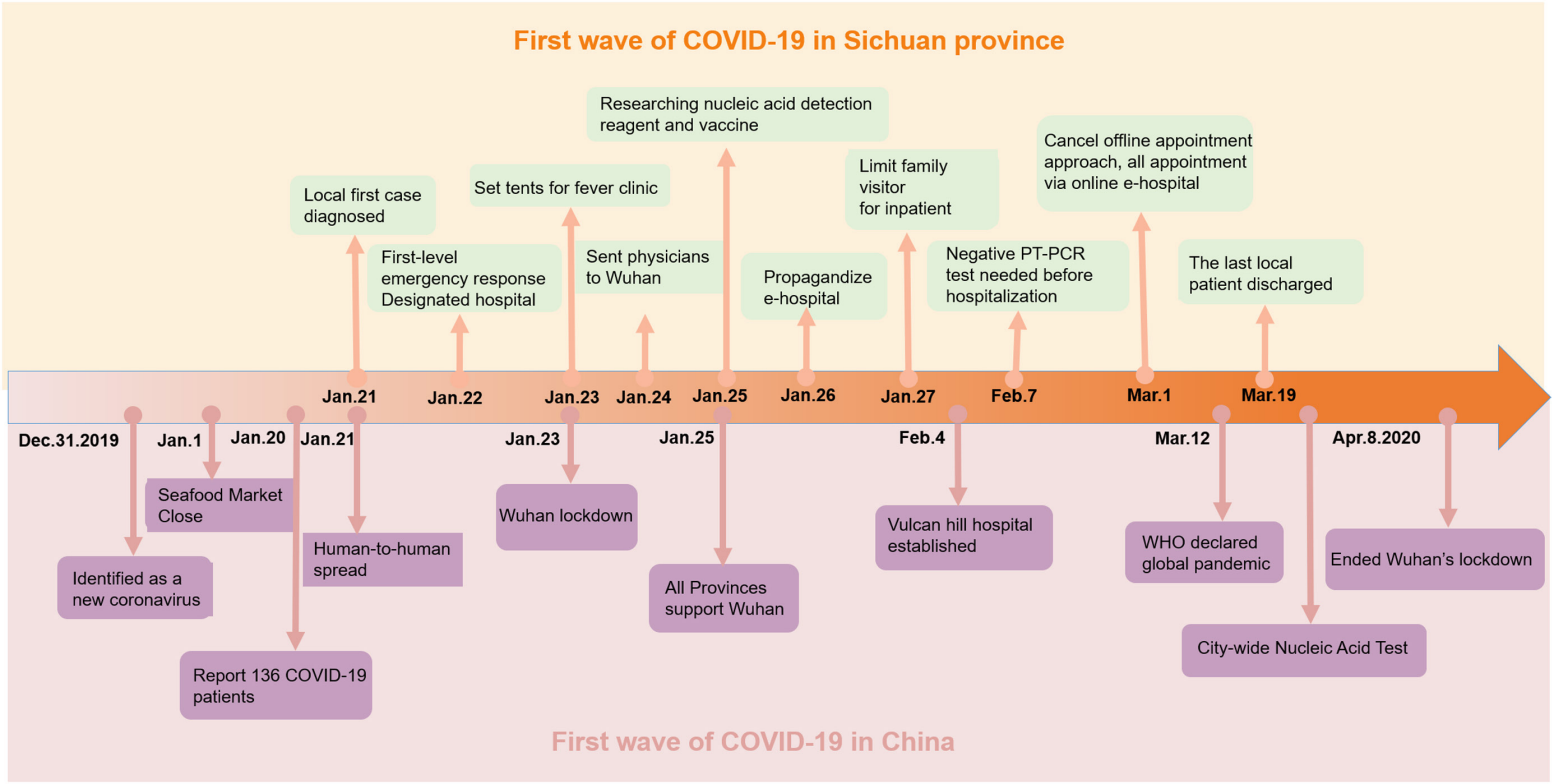
Lists of specific precautions adopted in West China Hospital, Sichuan Province and China since the COVID‐19 pandemic outbreak in January, 2020. After the outbreak of the COVID‐19 in China, Wuhan, the city with the most severe situation, instituted a citywide lockdown. Sichuan has conducted specific antipandemic measures, while the West China Hospital has concurrently carried out comparable antipandemic efforts.

Data were retrieved from the West China Hospital (WCH) in Chengdu, the capital of Sichuan province. WCH is one of the largest single‐site hospitals in the world and a leading medical center in China.^13^ With more than 4800 inpatient beds and outpatient clinics that can accommodate over 20,000 patients per day, WCH is the principal hospital serving southwestern China. In addition to the government's Dynamic Zero‐COVID Strategy, WCH has been implementing the following measures in response to the pandemic since the COVID‐19 outbreak in January 2020: expansion of COVID‐19 qRT‐PCR testing with the addition of five medical tents; screening with colored QR codes (a dynamic color‐based code with green representing no COVID‐19 exposure risk and the ability to move freely, yellow or red indicating medium or high exposure risk requiring 7–14 days self‐quarantine) before patients enter the hospital^14^; limiting family accompanies and visits for all inpatients; transferring appointment check‐in from on‐site to online; and requiring a negative qRT‐PCR test within 48 hours for hospitalization (Figure 1).^15^, ^16^ A total of 539 COVID‐19 cases were reported from January 21 to March 19, 2020, in Sichuan, and then 15 by March 31, 2021 (Figure S1).^17^

The WCH Cancer Center provides comprehensive cancer care services, including inpatient, surgery, outpatient physician visits, outpatient chemotherapy, outpatient radiotherapy, pharmacy, and laboratory, as well as telemedicine (Table S1), for the entire 8.4 million population of Sichuan province. The WCH Cancer Center operates at approximately 1.5 times the capacity of the only other comprehensive cancer center in Sichuan. Electronic medical records of all patients who utilized oncology services in the WCH Cancer Center between January 1, 2019, and March 31, 2021, were extracted from the Hospital Information System (HIS) and included in this analysis. The HIS was developed in 2007, and its data have been previously used elsewhere in health services research.^18^, ^19^ The data are stored, organized, audited (including validation with the Department of Operations & Management), and de‐identified by the Department of Information. Our research is being reported in line with the Epidemiology STROBE criteria, and was approved by the Institutional Review Board of WCH (No. 2022#1364).

### 2.2 Statistical analysis

We defined cancer care utilization by the total number of weekly visits to the cancer center. Segmented Poisson regression was performed to examine trends in cancer care utilization and to quantify changes associated with the COVID‐19 pandemic. The start (January 21, 2020, when the first case was reported in Sichuan) and end of the first wave (March 19, 2020, when the last case was discharged in Sichuan and no new cases were reported for 20 consecutive days) of COVID‐19 in Sichuan were specified as two change points. We defined weeks using the International Organization for Standardization calendar and started from January 7, 2019, the first full week in the study period, adjusting for holidays and seasonal variations (Appendix S1). The regression model was used to evaluate the immediate change in the first week following the two change points and the slope change in the weekly trend during the periods. A two‐sided *p* < 0.05 indicated statistical significance, with 95% confidence intervals (CIs) derived using the bootstrap technique.

The segmented regression was conducted for care utilization for overall patients and for patients diagnosed with the five most common cancer types: lung, colorectal, breast, gastric, and prostate cancers. We also examined whether the changes in utilization over time differed among subgroups by key sociodemographic factors, including age, residence area, and rurality, sex, health insurance coverage, employment status. All analyses were conducted using Python Version 3.9 (Python Software Foundation) and SPSS Version 25.0. Data were presented graphically using Python, Origin Version 2020 (OriginLab Corporation), and Excel Version 2019 (Microsoft).

## 3 RESULTS

There were 859,497 cancer care visits (telemedicine or in‐person visits) to the WCH Cancer Center between January 1, 2019, and March 31, 2021. The majority of the visits were by women (63.0%) and patients <60 years old (69.0%). In‐person cancer care visits accounted for 87.2% (749,562) of all visits, including 51.8% of the patients residing outside of Chengdu and 41.4% of rural inhabitants.

**FIGURE 2:**
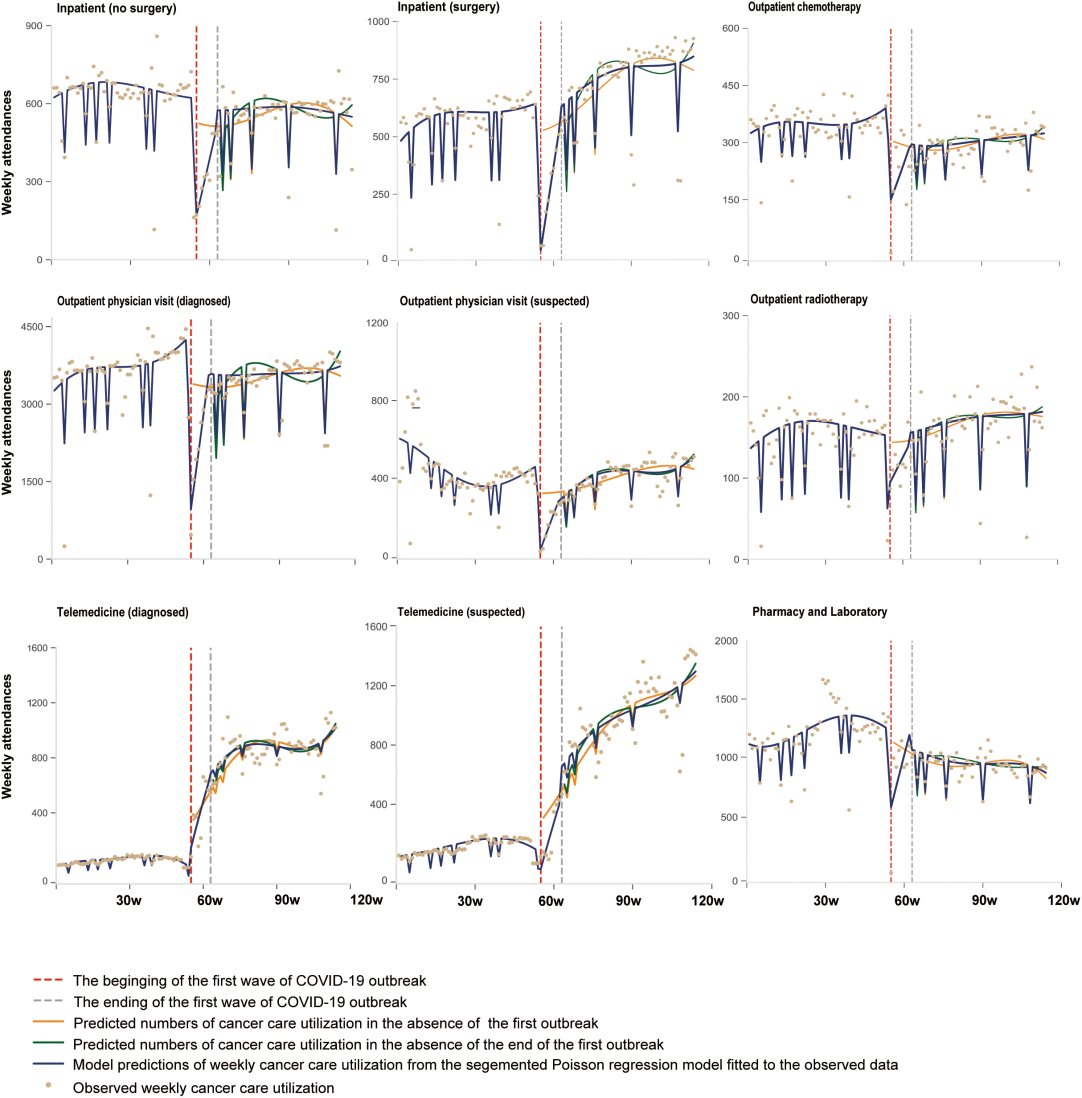
Poisson‐segmented regression analyses of changes in cancer care utilization in West China Hospital Cancer Center, stratified by service type. The first change point (the first wave of COVID‐19 outbreaks) is defined as January 21, 2020, when the first case was reported in Sichuan; the second change point (the end of the first wave of COVID‐19 outbreaks) is defined as the last case was discharged in Sichuan on Mar 19, 2020 and no new cases were reported for 20 consecutive days. Parameter estimates are tabulated in Table S2.

As depicted in Figure 2 and Table S2, sharp decreases were observed for all in‐person oncology care utilizations during the first week following the COVID‐19 onset (*p* < 0.005 for *β*_2_), ranging from −64.07 (95% CI [−108.44 to −19.69]) for radiotherapy to −3693.57 (95% CI [−4400.33 to −2986.81]) for outpatient physician visits. The utilization rebounded gradually after the first week, with varying slopes for different services. By March 19, 2020, the end of the first wave of COVID‐19, the use of almost all in‐person cancer care services was still lower than that of the pre‐COVID period. Since then, utilization for most of the services has slowly increased with a smaller upward slope compared with the trend during the outbreak period. However, most cancer care utilization did not rebound to pre‐COVID levels by March 31, 2021, 1 year after the first wave. The exceptions are the utilization of surgery services, which quickly exceeded the pre‐COVID level after the first wave (*β*_4_ = 69.04, 95% CI [−31.85 to 106.22] indicating trend change, *p* < 0.001), and the utilization of outpatient radiotherapy, which maintained a similar level as pre‐COVID and slowly increased to a level higher than pre‐COVID by the end of the study period (*β*_4_ = 6.79, 95% CI [−1.97 to 15.55], *p* = 0.127). In contrast, telemedicine for cancer care was activated by the COVID‐19 outbreak with an increasing trend for both diagnosed (*β*_4_ = 84.40, 95% CI [53.25 to 115.55], *p* < 0.001) and suspected (*β*_4_ = 78.07, 95% CI [36.96 to 119.18], *p* < 0.001) cancer patients after January 21, 2020, and then continued to increase, albeit at a slower rate, after March 19, 2020.

**FIGURE 3:**
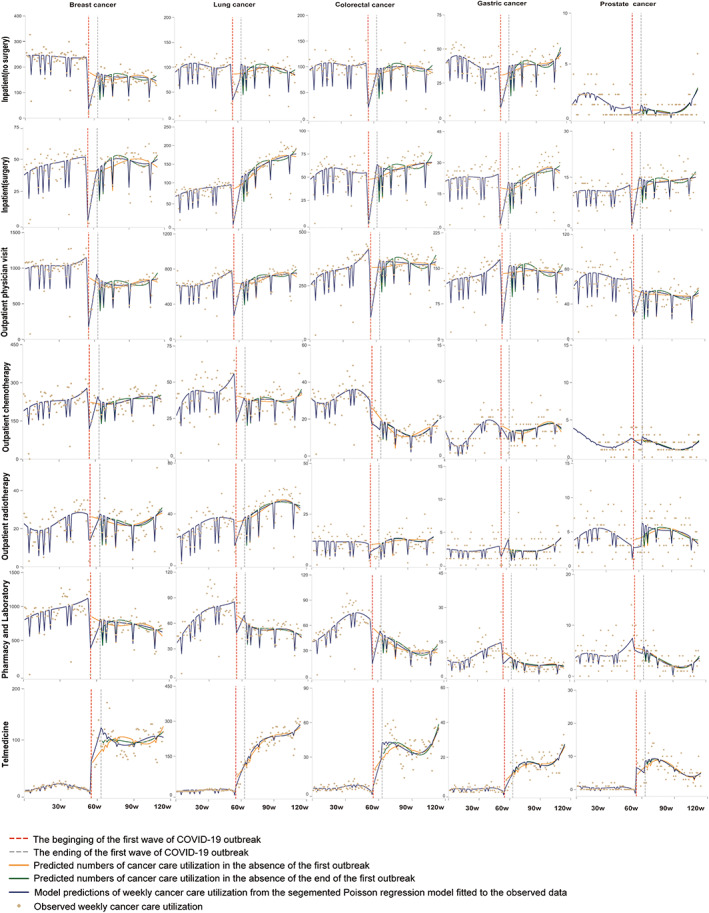
Poisson‐segmented regression analyses of changes in cancer care utilization in West China Hospital Cancer Center, stratified by service type and cancer type. See Table 1 for full relative change and 95% CIs, and testing for interactions.

Among patients with the five most common cancer types (Figure 3 and Table S3), large immediate reductions in all in‐person cancer care visits were observed for breast cancer, lung cancer, and colorectal cancer at the first week of COVID‐19 onset (*p* < 0.05 for *β*_2_), and then rebounded in the subsequent weeks. The decrease and slow recovery were most pronounced for breast cancer patients, with the greatest significant reduction observed in outpatient visits for breast cancer (*β*_2_ = −1086.98, 95% CI [−1278.87 to −893.09] indicating immediate change, *p* < 0.001). After March 19, 2020, quick rebounds and continuous increases were observed in surgery care utilization (*β*_3_ = 22.92, 95% CI [−13.98 to 59.83] indicating immediate change, *p* = 0.221; *β*_5_ = −13.28, 95% CI [−21.16 to −5.40] indicating trend change, *p* = 0.001) and outpatient radiotherapy (*β*_3_ = 1.21, 95% CI [−12.53 to 14.96], *p* = 0.861; *β*_5_ = −2.14, 95% CI [−5.07 to 0.80], *p* = 0.152) for lung cancer patients. In contrast, the utilization of online cancer services increased by 13.56 (95% CI [−17.65 to 44.77], *p* = 0.391) for breast cancer, 0.07 (95% CI [−37.25 to 37.39], *p* = 0.997) for lung cancer, 14.10 (95% CI [0.855 to 27.344], *p* = 0.037) for colorectal cancer, 1.69 (95% CI [−4.17 to 7.55], *p* = 0.569) for gastric cancer, and 3.32 (95% CI [−0.56 to 7.21], *p* = 0.093) for prostate cancer (all *β*_2_ indicating immediate change). By March 2021, online visits had surpassed their pre‐pandemic level; the most evident was for lung cancer, with an increasing trend of 12.54 (*β*_1_ = −2.48, 95% CI [−7.16 to 2.19]; *β*_4_ = 15.77, 95% CI [7.89 to 23.66], *p* < 0.001; *β*_5_ = −0.75, 95% CI [−8.72 to 7.22], *p* = 0.852; all indicating trend changes) per week.

**FIGURE 4:**
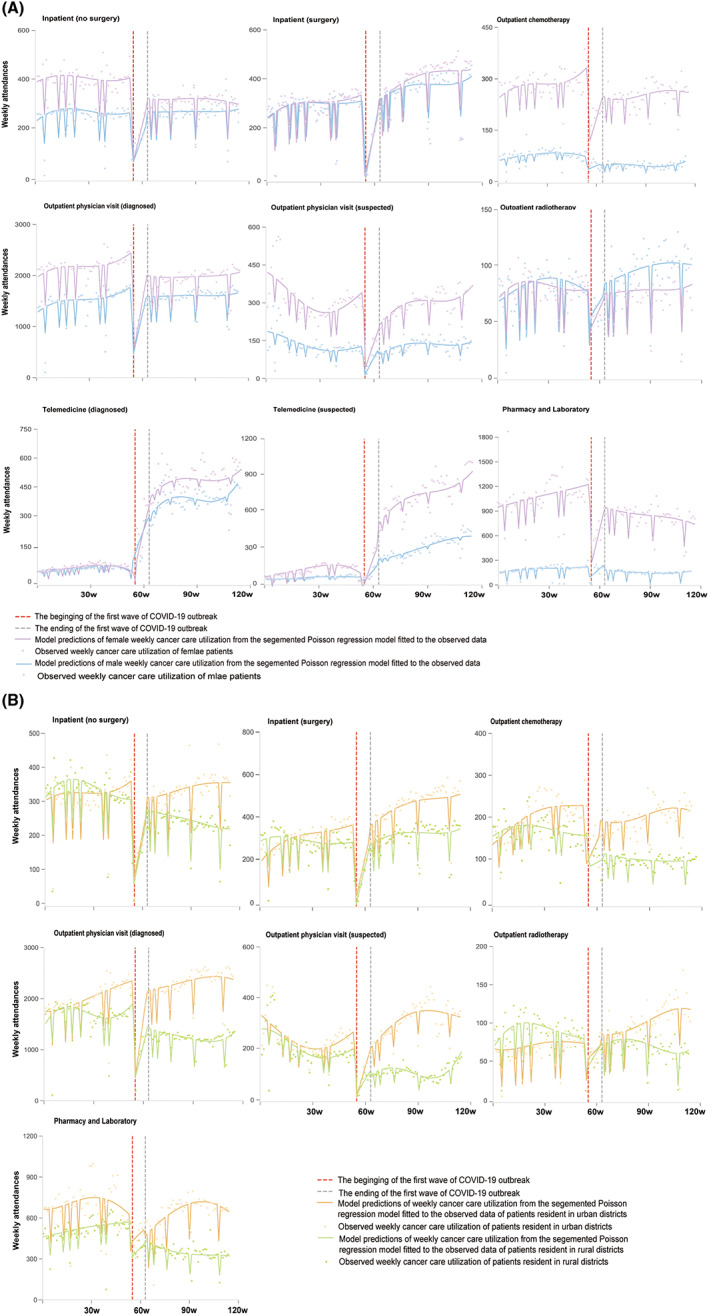
Poisson‐segmented regression analyses of changes in cancer care utilization in cancer center of West China Hospital, for a range of service types and patient sex and residence districts. Panel A represents different change among males and females, while Panel B represents different changes among urban and rural residents. See Appendix P8 for full relative change and 95% CIs, and testing for interactions.

The changes varied by sociodemographic subgroups (Figure 4A,B and Tables S4–S9 and Figures S2–S5). After March 19, 2020, the utilization of most in‐person cancer services rebounded more in men (vs. women, Figure 4A and Table S5), urban residents (vs. rural, Figure 4B and Table S5), patients from Chengdu (vs. outside Chengdu or Sichuan Province, Figure S2 and Table S6), and patients who paid via insurance (vs. uninsured, Figure S3 and Table S7). By the end of March 2021, utilization of outpatient radiation and surgery services had surpassed their pre‐pandemic levels for patients from urban areas, and those employed by government organizations or companies. In contrast, as depicted in Figure 4A and Table S4, the sharp increase in telemedicine cancer care utilization was significant for women suspected or diagnosed with cancer, as evidenced by rising trends per week for diagnosed patients (*β*_1_ = −5.08; *β*_4_ = 48.94, 95% CI [30.18 to 67.69], *p* < 0.001; *β*_5_ = −27.14, 95% CI [−46.11 to −8.18], *p* = 0.006; all indicating trend changes) and for suspected patients (*β*_1_ = −11.05; *β*_4_ = 60.11, 95% CI [29.71 to 90.51], *p* < 0.001; *β*_5_ = −16.43, 95% CI [−47.17 to 14.31], *p* = 0.292).

## 4 DISCUSSION

Leveraging a large medical administrative dataset from a major medical facility in southwest China, we assessed the impact of the COVID‐19 pandemic on the utilization of various oncology services in the 14 months following the pandemic's onset in January 2020. Our study illustrates substantial reductions in all in‐person cancer services use during the first wave of the pandemic, and reveals that utilization of most services had not returned to pre‐COVID levels by March 31, 2021, despite few new COVID‐19 cases being reported in the region. An exception was identified for increased surgery and outpatient radiation visits among lung cancer patients. These care disruptions were more evident for patients who were women, breast cancer patients, living in remote areas and rural areas, or uninsured.

Consistent with observations from other regions in China and other countries, our study revealed a remarkable decline in most cancer care utilization following the COVID‐19 outbreak and a slow recovery afterwards.^6^, ^7^, ^20^, ^21^, ^22^ For example, a previous study found that in comparison with the pre‐COVID‐19 period, diagnosis and surgery for breast cancer care in Wuhan had striking delays as of March 31, 2020.^23^ However, unlike other countries and Wuhan, where reduction in care utilization was primarily attributed to constrained service capacity and mandatory lockdown,^24^ the WCH did not exhaust its capacities (including human resources),^25^ and the WCH Cancer Center did not proactively reduce the intensity of care provided. Moreover, the disruptions in most cancer services lasted throughout the study period despite few cases being reported in Sichuan since March 2020. Therefore, the reduction of cancer services utilization in the WCH Cancer Center was most likely attributable to massive mobility restrictions under the Dynamic Zero‐COVID Strategy and the fear of COVID‐19 infection within medical facilities. With travel restrictions adopted in other provinces following the Wuhan lockdown, the population flow across China reached its minimum level on February 15, 2020, dropping to nearly a quarter of the level on January 22, 2020.^26^, ^27^ Patient mobility to medical facilities was restricted, with exceptions for special permission or ambulatory care once a case is confirmed in their area.^28^ Meanwhile, fear of visits to medical facilities and delaying/forgoing care was reported, for instance, in an antenatal care web‐based survey conducted in Guangzhou, China, during the pandemic.^29^ Delayed cancer care leads to diagnosis at a more advanced stage, increased likelihood of relapse, and shortened survival time. Considering the delayed care in diagnosis and treatment in southwestern China, more efforts are urgently needed to mitigate the downstream burden of cancer deaths and related economic costs under the Dynamic Zero‐COVID policy.^30^ Moreover, programs to improve health literacy and communication during pandemics should be prioritized to assist patients in overcoming the fear of contraction in medical facilities and to ensure timely visits to care providers, especially for cancer patients.^31^

An exception in our data is that surgery, especially for lung cancer patients, rebounded quickly after the COVID‐19 wave in early 2020 and surpassed the pre‐COVID level. An international study reported that one in seven patients refused to receive planned operations for pandemic‐related reasons during the months following the COVID‐19 pandemic outbreak.^32^ Nevertheless, the increase in surgery demand for lung cancer in WCH is likely related to the respiratory evaluation for COVID‐19 detection, as an increase in pulmonary nodule detection has been reported anecdotally as a spillover effect of the widely conducted chest radiograph as a method for COVID‐19 diagnosis.^33^ Future studies should evaluate the effect of the increased use of this methodology on the lung cancer burden.

Another exception in our data is that outpatient radiotherapy recovered to a level higher than before the pandemic as of March 31, 2021. This is in line with the reports from multiple countries that modifications in cancer care treatment practice since the pandemic had minimized exposure to the virus and potentially improved outcomes.^34^, ^35^ For example, short‐course radiation therapy was recommended as an alternative for delayed surgery treatment modalities with higher infection risk by the European Society for Medical Oncology.^36^ A study from England reported that the use of radiotherapy for rectal cancer in 2020 increased by 44% compared with that in 2019.^37^ Another study in the UK found that radiation use decreased only 10%, compared to a reduction of 40% for surgery for cancer patients up to April 2020.^38^ In Latin America, over 97% of practices continued to provide radiation services during the pandemic.^39^ The long‐term effects of this shift from other treatment modalities to radiotherapy on recurrence, prognosis, and survival would be an important area for future research.

The explosion of telemedicine since the onset of the COVID‐19 pandemic has been reported in various healthcare systems worldwide.^40^, ^41^ To the best of our knowledge, our study represents the first of such reports in China. In Western countries, policies and laws that require insurers to cover telemedicine are considered the main facilitator of the fast and broad uptake of telemedicine,^42^ while in our study population, where no emergency telehealth laws and reimbursement were implemented at the provincial or national level, the increases in telehealth visits can be attributable to efforts by the WCH in promoting online clinic services on social media, the quick assemblage of clinical experts, and the waiving of registration fees for these online services.^43^ The “digital divide” reported in Western countries, where those without smart devices or internet access, older adults, rural residents, and those with limited digital literacy were more likely to be left behind,^44^, ^45^ should not be overlooked in China. For instance, we found that those aged 75 years or older had the smallest increase in telehealth visits after the pandemic among all age groups. Policies strengthening the digital infrastructure and programs improving digital literacy at the individual and community levels are essential for health equity in telehealth use.^46^ Moreover, the tradeoffs of shifting to telehealth and video consultations, including missed cues, reduced examination, and loss of personal interaction holistic assessment, and their long‐term impact on efficacy, safety, and costs, warrant future evaluation.^47^, ^48^, ^49^

Our study found that the reduction in cancer care utilization was more marked among women. Our data also showed that patients with breast cancer, the most common cancer in Chinese women,^50^ experienced the greatest decrease in various services compared to patients with other cancer types. This pattern has also been reported in studies in China and the Netherlands.^21^, ^51^ The findings may reflect several sex differences during the pandemic: women were more likely to become unemployed and lose insurance coverage^52^, ^53^; also, women were more likely to have increased family responsibilities such as taking care of children and elderly individuals when schools, nursing homes and long‐term care facilities were closed.^54^, ^55^ Under financial constraints and other life priorities, women may have a higher risk of delaying or forgoing required treatment. In preparation for the cancer care aftermath of COVID‐19, developing targeted screening and treatment plans for women and breast cancer patients may be required.

As the best‐regarded medical facility in western China, WCH serves the whole population of southwest China. However, we identified significant geographic variation in care utilization changes following the COVID‐19 pandemic among patients receiving care at WCH Cancer Center, with patients from outside of Sichuan province and rural areas experiencing the worst care disruptions. Historically, rural populations have had poor healthcare resources and have long depended on traveling to hospitals in cities for serious diseases.^56^ About 95% of the Chinese population receive some form of public health insurance, but the level of coverage varies significantly by geography, and rural residents tend to receive less coverage than their urban compatriots.^57^, ^58^ In 2019, the government's annual expenditure for urban employees' basic medical insurance was $507 per capita, compared with $113 for rural residents' basic medical insurance.^59^ Moreover, during the pandemic, to receive care from WCH, patients were required to reside in a low‐risk area as defined by the local government based on epidemiological investigation, and needed to overcome various intercity and interprovincial travel restrictions and meet various testing requirements. Coupled with the long‐standing unbalanced medical resource distribution and lack of adequate insurance coverage, COVID‐19‐related restrictions may have exacerbated the geographic disparities in timely access to quality care. Our findings highlight the urgent need to address the structural disparities among rural residents through increasing investment in rural county hospitals^60^ and insurance reforms^61^ both in response to the COVID‐19 pandemic and for the long term.^62^ During the pandemic, in addition to lifting unnecessary travel restrictions and testing requirements, policies such as tax exemption, rent reduction, and loan postponement, which have shown to be effective in providing relief to disadvantaged populations in other countries,^63^, ^64^ could help vulnerable rural patients access affordable care and survive the catastrophe.

The strengths of our study include its use of real‐world data covering a large population and spanning a relatively long period of the pandemic, which enabled us to control for seasonal patterns, secular trends, and multiple potential confounding factors and powered us to examine disparities by key sociodemographic factors. Another strength is the availability of various cancer services information from a leading hospital in southwestern China.

### 4.1 Limitations

This study has several limitations. The data include attendance from a single cancer center. Therefore, findings are not generalizable to the population, although most cancer patients in China choose tertiary hospitals for diagnosis and treatment as cancer services often require better medical equipment and sources.^65^ This lack of generalizability may be particularly pronounced for the older population, as cancer patients older than 75 in China may prefer treatment and long‐term cancer care visits in local clinics over center hospitals in metropolitan cities.^66^, ^67^ Also, our de‐identified data were based on visits but not individual patients. In addition, because of available data limitations, we were not able to evaluate changes in cancer stage at presentation or survival outcomes. Lastly, we were not able to consider COVID‐19 vaccinations in the analyses because we do not have access to such information.

## 5 CONCLUSIONS

Using electronic medical records from the cancer center of a large hospital, we found that cancer care disruptions were widespread during the pandemic in southwestern China, and most in‐person care services declined and had not fully recovered even over a year after the first wave ended, despite few COVID‐19 cases in the period. We observed an increase in surgery primarily for lung cancer, suggesting a spillover effect of chest radiography as a diagnosis tool for COVID‐19 cases; we also documented an increase in outpatient daycare radiotherapy utilization, possibly initiated as an alternative to other treatments. We also observed a significant increase in teleconsultation use. The long‐term effects of these utilization changes on cancer care and outcomes warrant further examination. Moreover, we identified disparities among breast cancer patients, rural residents, and uninsured patients, who experienced the most cancer care disruptions during the pandemic. Our findings highlight the importance of national and local health policies that consider equitable and timely access to oncology care in during emergency response. Future studies are warranted to monitor the long‐term effects of the pandemic and response policies on cancer care and outcomes.

## AUTHOR CONTRIBUTIONS

**Peiyi Li:** Conceptualization (lead); data curation (lead); formal analysis (lead); methodology (equal); writing – original draft (lead). **Yajuan Zhu:** Conceptualization (supporting); data curation (supporting); formal analysis (supporting); methodology (supporting); visualization (lead). **Yaqiang Wang:** Formal analysis (equal); software (lead); visualization (supporting). **Xiaoyu Liu:** Conceptualization (supporting); methodology (supporting); writing – original draft (supporting). **Xiang Fang:** Data curation (supporting); visualization (supporting). **Yuanxin Hou:** Data curation (supporting); formal analysis (supporting); visualization (supporting). **Rujun Zheng:** Methodology (supporting); resources (equal). **Junying Li:** Project administration (supporting); resources (supporting). **Bo Zhang:** Methodology (lead). **Zhuo Chen:** Supervision (supporting); writing – review and editing (supporting). **Chengdi Wang:** Funding acquisition (equal); methodology (supporting); project administration (equal); writing – review and editing (supporting). **Tao Zhu:** Project administration (supporting); resources (supporting); supervision (supporting); writing – review and editing (supporting). **Weimin Li:** Conceptualization (supporting); funding acquisition (lead); project administration (lead); supervision (lead). **Xuesong Han:** Project administration (supporting); supervision (supporting); writing – review and editing (lead).

## FUNDING INFORMATION

This study was funded by the National Natural Science Foundation of China (91859203 to WL, 72207174 to PL), the Science and Technology Project of Sichuan (2020YFG0473 to WL), China Postdoctoral Science Foundation (2022M722262 to PL), and the Postdoctoral Program of West China Hospital, Sichuan University (2020HXBH084 to CW, 2023HXBH009 to PL). The funders had no role in the design and conduct of the study; collection, management, analysis, or interpretation of the data; preparation, review, or approval of the manuscript; or decision to submit the manuscript for publication.

## CONFLICT OF INTEREST STATEMENT

We declare no competing interests.

## DISCLOSURES

XH has received a grant from AstraZeneca for research outside of the current study.

## Supporting information


Appendix S1.

## Data Availability

Individual data underpinning the findings described in this study are accessible to other qualified medical researchers upon request to the corresponding author. Requestors will need to sign an agreement for data access. Data will be accessible at 12 months and ending at 36 months after Article publication.
