# Supplementary material for: Impact of the COVID‐19 pandemic on cancer healthcare utilization in southwestern China on March 2021
Source: Cancer Med. 2023 May 11;12(12):13821–33. doi: 10.1002/cam4.6028 (PMC10315764; doi:10.1002/cam4.6028)
Supplement: Supplementary file 1 — Appendix S1. [file CAM4-12-13821-s001.docx]

**Impact of the COVID-19 pandemic on cancer healthcare utilization in Southwestern China**

**Supplementary Appendix**

**eFigure1.** The monthly confirmed and deaths numbers in China and Sichuan Province since the pandemic outbreak

**eFigure2.** Poisson segmented regression analyses of changes in cancer care utilizations in West China Hospital Cancer Center, stratified by service type and residence distance

**eFigure3.** Poisson segmented regression analyses of changes in cancer care utilization in West China Hospital Cancer Center, stratified by service type and payment method

**eFigure4.** Poisson segmented regression analyses of changes in cancer care utilization in West China Hospital Cancer Center, stratified by service type and occupation

**eFigure5.** Poisson segmented regression analyses of changes in cancer care utilization in West China Hospital Cancer Center, stratified by service type and age

**eText.** Regression Equation

**eTable1.** Admissions description of cancer care services in West China Hospital Cancer Center

**eTable2**. Poisson segmented regression analyses of changes in cancer care utilizations in West China Hospital Cancer Center, stratified by service type

**eTable3.** Poisson segmented regression model of the impact of the COVID-19 pandemic on cancer care utilization related to the top five cancer types in West China Hospital Cancer Center.

**eTable4.** Changes in cancer care utilizations in West China Hospital Cancer Center, stratified by service type and sex

**eTable5.** Changes in cancer care utilizations in West China Hospital Cancer Center, stratified by service type and rurality

**eTable6.** Changes in cancer care utilization in West China Hospital Cancer Center, stratified by service type and residence distance

**eTable7.** Changes in cancer care utilization in West China Hospital Cancer Center, stratified by service type and payment method

**eTable8.** Changes in cancer care utilization in West China Hospital Cancer Center, stratified by service type and occupation

**eTable9.** Changes in cancer care utilization in West China Hospital Cancer Center, stratified by service type and age


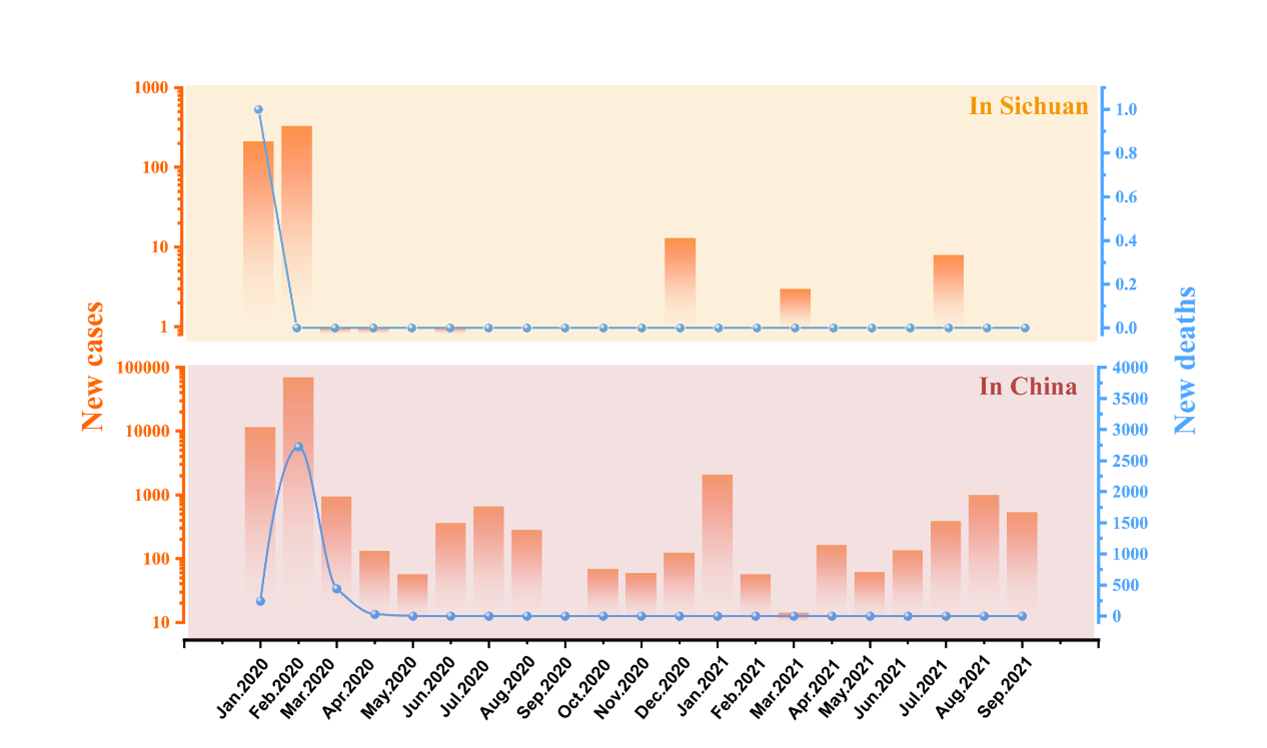
eFigure1. The monthly confirmed and deaths numbers in China and Sichuan Province since the pandemic outbreak.

The height of the column represents the cumulative number of confirmed cases per month, and the line graph represents the number of deaths per month due to the epidemic.

eFigure2. Poisson segmented regression analyses of changes in cancer care utilizations in West China Hospital Cancer Center, stratified by service type and residence distance.


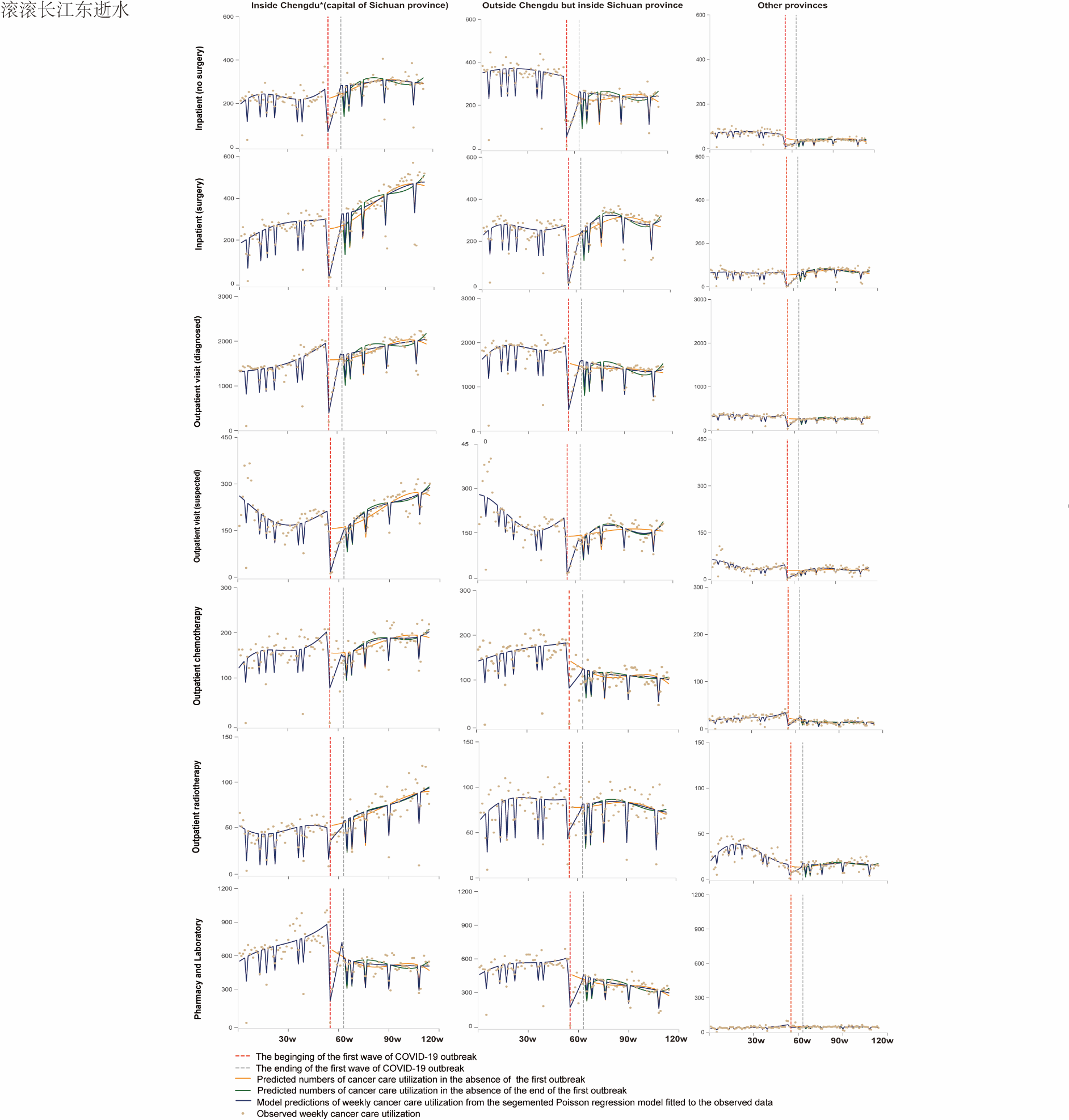


eFigure3. Poisson segmented regression analyses of changes in cancer care utilization in West China Hospital Cancer Center, stratified by service type and payment method.


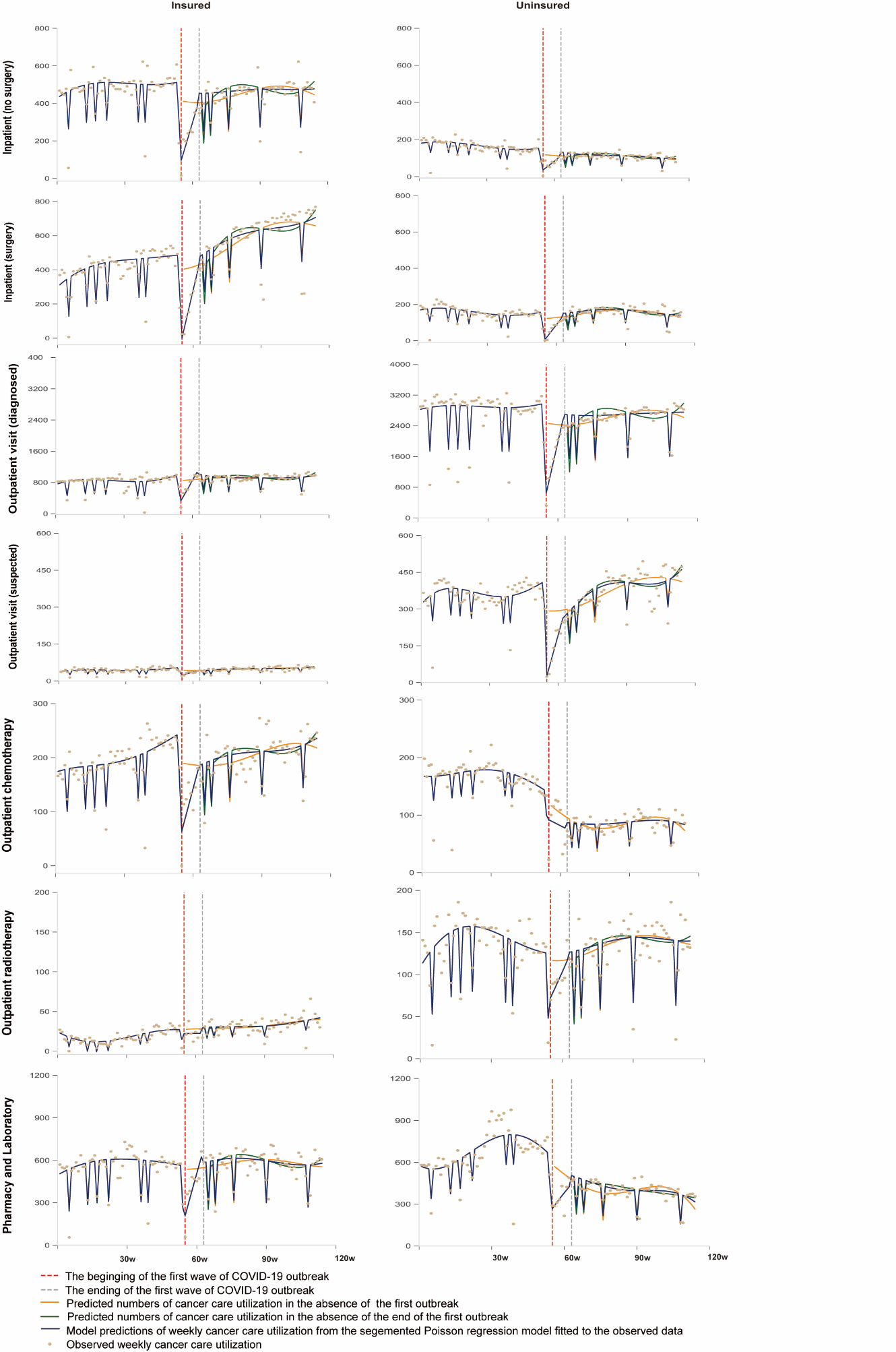


eFigure 4. Poisson segmented regression analyses of changes in cancer care utilization in West China Hospital Cancer Center, stratified by service type and occupation.


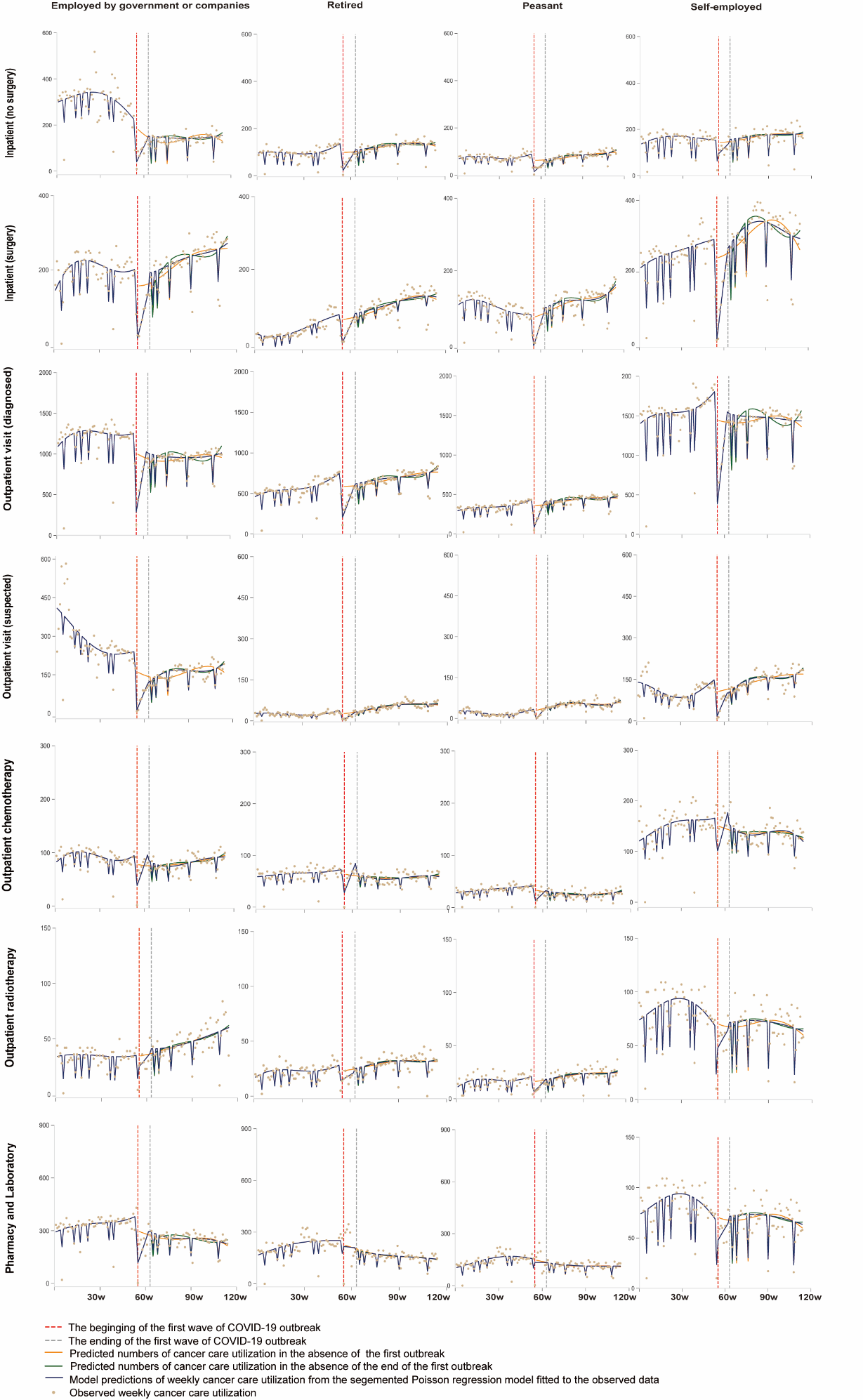


eFigure 5. Poisson segmented regression analyses of changes in cancer care utilization in West China Hospital Cancer Center, stratified by service type and age.

**
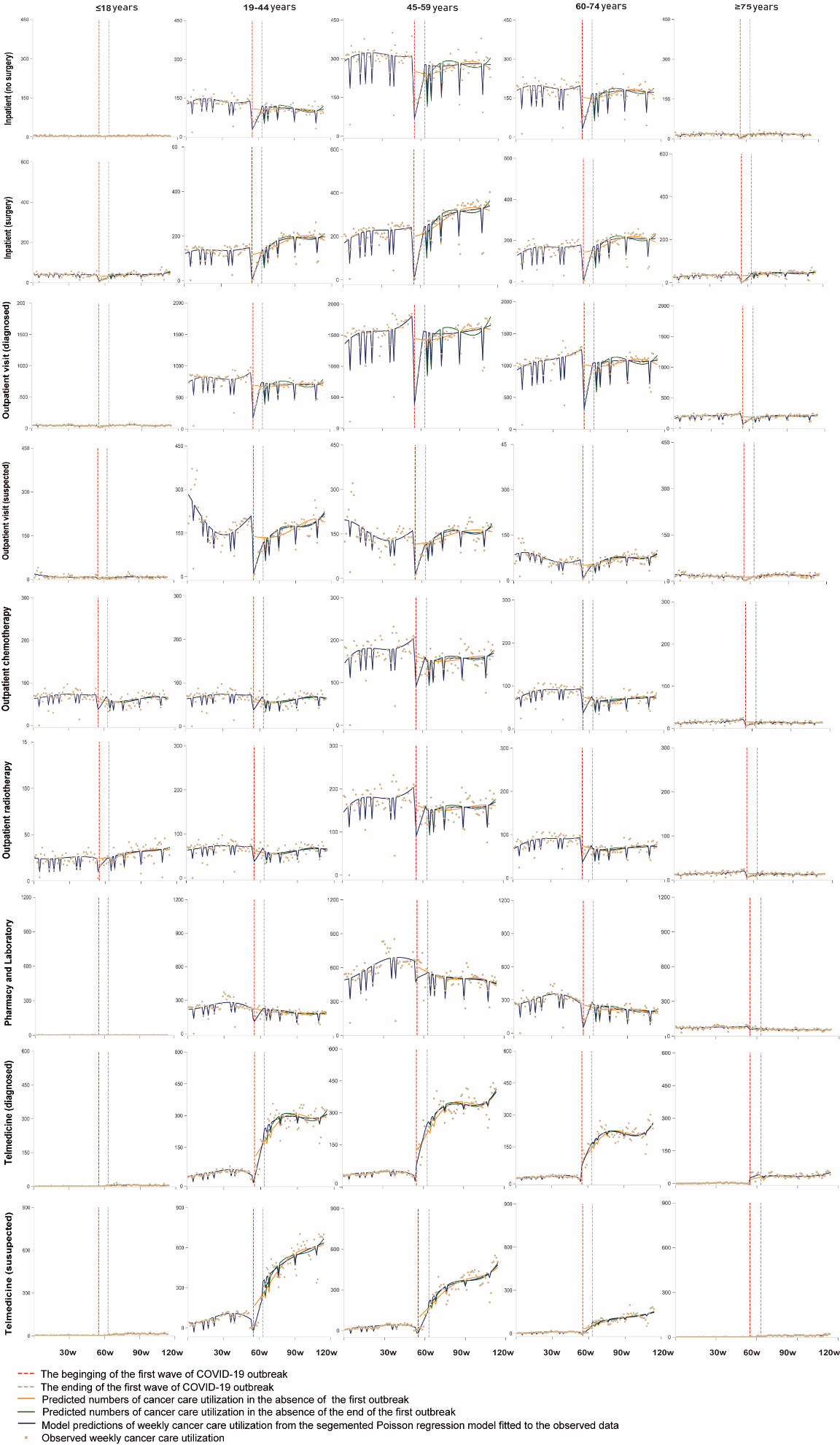
**

**eText: Regression Equation:**

$${Attandances at time(\mu}_{t}) =\beta_{0}+\beta_{1}t+\beta_{2}X_{t}^{\left( 1 \right)}+\beta_{3}X_{t}^{\left( 2 \right)}+\beta_{4}\left( t-T_{1} \right)X_{t}^{\left( 1 \right)}+\beta_{5}{\left( t-T_{2} \right)X}_{t}^{\left( 2 \right)}+\beta_{6}H_{t}+\sum_{k=1,2} \left[ \beta_{ck}\cos\left( 2k\pi m_{t} \right)+\beta_{sk}\sin\left( 2k\pi m_{t} \right) \right]+\epsilon_{t}$$

$\mu_{t}$ is the number of weekly utilization, represents the time series counts of patients for different healthcare service types at time (week) *t*. *t* is the study time from the start to the end of the study; $X_{t}^{\left( 1 \right)}$ is a binary indicator coded as 0 before the first wave outbreak of COVID-19 in Sichuan and 1 after the outbreak (Jan 21, 2020), while$X_{t}^{\left( 2 \right)}$ is a binary indicator coded as 0 before the end of first wave in Sichuan and 1 after the end (Mar 19, 2020). Moreover, $T_{1}$ is the first time point after the outbreak of COVID-19, $T_{2}$ is the first time point after the end of first wave of COVID-19 in Sichuan (Mar 19, 2020). Each year China has 7 major public holidays with 3-7 days off, during which, hospitals are shut and only some staff on duty for emergency. Thus, $H_{t}$is a binary variable, and coded as 1 for all weeks with holidays. $m_{t}$is the variable indicating monthly (the value of $m_{t}$depends on the month at time t, equal to 1/12 for January, 2/12 for February, etc.), and $\epsilon_{t}$is the random error term. The coefficient $\beta_{0}$ is the regression intercept representing the starting level of the aggregated outcome variable, $\beta_{1}$ is the slope or trajectory of the aggregated outcome variable before the pandemic, $\beta_{2}$and $\beta_{3}$represent the change in the level of the outcome that occurs immediately in the first week of change point, indicating an immediate effect of onset and end of the first COVID-19 wave ;and$\beta_{4}$and $\beta_{5}$represent the difference between pre-COVID and during-COVID slopes and the difference between during-COVID and post-COVID slopes of the aggregated outcome, respectively, indicating the effect in terms of changes in trends. In addition, $\beta_{6}$represent the influence of holidays, while $\beta_{ck}$and $\beta_{sk}$ represent the influence of season.

| **Cancer care service type** | | | **Admission population*** | | **Care description** | |
| --- | --- | --- | --- | --- | --- | --- |
| In-person | Inpatient non-surgery visit | Patients diagnosed with cancer, whose physical condition are usually too weak to have outpatient treatments or their treatments require hospitalization | | Hospitalized for systemic treatment, including infusion of chemotherapeutic drugs, radiation, palliative treatment. | |  |
|  | Inpatient surgery visit |  |  | Hospitalized for treatment, mainly including surgery, a range of postoperative anti-cancer treatment such as infusion of chemotherapeutic drugs, radiation, palliative treatment. | |  |
|  | Outpatient physician visit | Patients diagnosed with cancer | | Seek further high-quality treatment, and follow-up | |  |
|  |  | Patients suspected with cancer | | To determine if the individual have malignant diseases | |  |
|  | Outpatient chemotherapy | Patients diagnosed with cancer, whose physical condition and chemotherapy plan are suitable for day-care | | Infusion of chemotherapeutic drugs without hospitalization, allow patients to walk home the same day of chemotherapy infusion | |  |
|  |  |  |  | Infusion of immunotherapy and targeted drugs without hospitalization, allow patients to walk home the same day of radiotherapy infusion, accounting for 10.8% of total chemotherapy attendances | |  |
|  | Outpatient radiotherapy | Patients diagnosed with cancer, whose physical condition and radiotherapy plan are suitable for day-care | | Receive external beam radiation without hospitalization, allow patients to walk home the same day of radiation | |  |
|  | Pharmacy and Laboratory | Patients diagnosed with cancer, who need regularly surveillance and medication | | Only prescribe examination (e.g., CT, MRI, X-ray), blood test, and medication, but no treatment consultation provides | |  |
| Online | Telemedicine | Patients diagnosed with cancer | | Seek further high-quality treatment, follow-up | |  |
|  |  | Patients suspected of cancer | | To determine if the individual have malignant diseases | |  |

**eTable 1. Admissions description of cancer care services in West China Hospital Cancer Center**.

* Both suspected and diagnosed cancer patients utilize outpatient physician visits and telemedicine units, and only patient with a cancer diagnosis use other services provided by the cancer center.

**eTable 2. Poisson segmented regression analyses of changes in cancer care utilizations in West China Hospital Cancer Center, stratified by service type.**

|  | **Coef.** | **P value** | **95%CI (Lower)** | **95%CI(Upper)** |
| --- | --- | --- | --- | --- |
| **Inpatient unit(non-surgery)** | | | | |
| Constant β_0_ | 512.213 | 0.1335 | -159.563 | 1183.990 |
| Time(weeks)β_1_ | 1.472 | 0.8668 | -15.892 | 18.836 |
| Seasonality terms β_ck_ and β_sk_ |  |  |  |  |
| Cos1 | 63.641 | 0.7940 | -418.381 | 545.664 |
| Sin1 | 100.020 | 0.6413 | -324.542 | 524.583 |
| Cos2 | 41.646 | 0.5319 | -90.036 | 173.329 |
| Sin2 | 21.331 | 0.7454 | -108.627 | 151.288 |
| Pandemic outbreak β_2_ | -496.156 | 0.0000 | -644.443 | -347.869 |
| Pandemic remission β_3_ | 98.266 | 0.408 | 63.873 | 64.353 |
| Outbreak-time interaction β_4_ | 44.8584 | 0.0032 | 15.311 | 73.858 |
| Remission-time interaction β_5_ | -44.377 | 0.0037 | -73.976 | -14.778 |
| Holiday β_6_ | -233.223 | 0.0000 | -284.065 | -182.380 |
| **Inpatient unit (surgery)** | | | | |
| Constant β_0_ | 86.689 | 0.8407 | -766.633 | 940.111 |
| Time(weeks)β_1_ | 8.005 | 0.4733 | -14.052 | 30.062 |
| Seasonality terms β_ck_ and β_sk_ |  |  |  |  |
| Cos1 | 260.200 | 0.4013 | -352.087 | 872.487 |
| Sin1 | -291.600 | 0.2861 | -247.698 | 830.899 |
| Cos2 | 121.113 | 0.1540 | -46.157 | 288.383 |
| Sin2 | -49.106 | 0.5565 | -214.184 | 115.971 |
| Pandemic outbreak β_2_ | -713.086 | 0.0000 | -901.447 | -524.725 |
| Pandemic remission β_3_ | 101.348 | 0.2562 | -74.677 | 277.374 |
| Outbreak-time interaction β_4_ | 69.039 | 0.0004 | 31.854 | 106.223 |
| Remission-time interaction β_5_ | -62.108 | 0.0014 | -99.707 | -24.510 |
| Holiday β_6_ | -296.349 | 0.0000 | -360.931 | -231.767 |
| **Outpatient physician visit unit (diagnosed)** | | | | |
| Constant β_0_ | 1178.457 | 0.4671 | -2023.337 | 4380.250 |
| Time(weeks)β_1_ | 76.421 | 0.0699 | -6.340 | 159.182 |
| Seasonality terms β_ck_ and β_sk_ |  |  |  |  |
| Cos1 | 1601.145 | 0.1699 | -696.248 | 3898.538 |
| Sin1 | -261.206 | 0.7985 | -2284.740 | 1762.329 |
| Cos2 | 426.275 | 0.1809 | -201.347 | 1053.898 |
| Sin2 | -127.149 | 0.6848 | -746.544 | 492.245 |
| Pandemic outbreak β_2_ | -3693.569 | 0.0000 | -4400.331 | -2986.807 |
| Pandemic remission β_3_ | 36.331 | 0.9133 | -624.156 | 696.818 |
| Outbreak-time interaction β_4_ | 311.078 | 0.0000 | 171.557 | 450.599 |
| Remission-time interaction β_5_ | -386.877 | 0.0000 | -527.952 | -245.802 |
| Holiday β_6_ | -1208.936 | 0.0000 | -1451.260 | -996.613 |
| **Outpatient physician visit unit (suspected)** | | | | |
| Constant β_0_ | 247.079 | 0.4754 | -437.058 | 931.217 |
| Time(weeks)β_1_ | 4.689 | 0.6001 | -12.994 | 22.373 |
| Seasonality terms β_ck_ and β_sk_ |  |  |  |  |
| Cos1 | 266.008 | 0.2850 | -224.883 | 756.900 |
| Sin1 | 34.688 | 0.8739 | -397.705 | 467.081 |
| Cos2 | 96.006 | 0.1587 | -38.101 | 230.112 |
| Sin2 | -159.685 | 0.0185 | -292.038 | -27.332 |
| Pandemic outbreak β_2_ | -473.602 | 0.0000 | -624.617 | -332.587 |
| Pandemic remission β_3_ | -1.345 | 0.9850 | -142.477 | 139.787 |
| Outbreak-time interaction β_4_ | 28.208 | 0.0634 | -1.604 | 58.020 |
| Remission-time interaction β_5_ | -21.533 | 0.1596 | -51.676 | 8.611 |
| Holiday β_6_ | -147.984 | 0.0000 | -199.762 | -96.207 |
| **Outpatient chemotherapy unit** | | | | |
| Constant β_0_ | 134.967 | 0.4879 | -249.519 | 519.454 |
| Time(weeks)β_1_ | 7.480 | 0.1386 | -2.459 | 17.418 |
| Seasonality terms β_ck_ and β_sk_ |  |  |  |  |
| Cos1 | 144.660 | 0.3008 | -131.222 | 420.542 |
| Sin1 | -71.146 | 0.5627 | -314.143 | 171.850 |
| Cos2 | 40.647 | 0.2873 | -34.720 | 235.435 |
| Sin2 | -2.996 | 0.9365 | -77.377 | 71.384 |
| Pandemic outbreak β_2_ | -262.899 | 0.0000 | -347.770 | -178.029 |
| Pandemic remission β_3_ | 12.287 | 0.7593 | -67.026 | 91.600 |
| Outbreak-time interaction β_4_ | 12.604 | 0.1388 | -4.150 | 29.358 |
| Remission-time interaction β_5_ | -21.501 | 0.0134 | -38.441 | -4.560 |
| Holiday β_6_ | -90.383 | 0.0000 | -119.482 | -61.283 |
| **Outpatient radiotherapy unit** | | | | |
| Constant β_0_ | 78.727 | 0.4391 | -122.312 | 279.767 |
| Time(weeks)β_1_ | 0.837 | 0.7501 | -4.360 | 6.033 |
| Seasonality terms β_ck_ and β_sk_ |  |  |  |  |
| Cos1 | 38.513 | 0.5976 | -105.740 | 182.766 |
| Sin1 | 58.844 | 0.3605 | -68.214 | 185.901 |
| Cos2 | 16.546 | 0.4069 | -22.862 | 55.954 |
| Sin2 | 3.804 | 0.8466 | -35.088 | 42.696 |
| Pandemic outbreak β_2_ | -64.065 | 0.0051 | -108.442 | -19.688 |
| Pandemic remission β_3_ | 16.675 | 0.4270 | -24.796 | 58.146 |
| Outbreak-time interaction β_4_ | 6.791 | 0.1273 | -1.970 | 15.551 |
| Remission-time interaction β_5_ | -5.483 | 0.2224 | -14.341 | 3.375 |
| Holiday β_6_ | -89.680 | 0.0000 | -104.896 | -74.465 |
| **Pharmacy and Laboratory unit** | | | | |
| Constant β_0_ | 2062.131 | 0.0008 | 884.555 | 3239.707 |
| Time(weeks)β_1_ | -21.266 | 0.1688 | -51.704 | 9.172 |
| Seasonality terms β_ck_ and β_sk_ |  |  |  |  |
| Cos1 | -714.683 | 0.0965 | -559.634 | 130.269 |
| Sin1 | 8.347 | 0.9823 | -735.863 | 752.556 |
| Cos2 | -226.420 | 0.0545 | -457.250 | 4.409 |
| Sin2 | 146.779 | 0.2042 | -81.022 | 374.580 |
| Pandemic outbreak β_2_ | -759.919 | 0.0000 | -1019.855 | -499.984 |
| Pandemic remission β_3_ | -119.277 | 0.3324 | -362.186 | 123.632 |
| Outbreak-time interaction β_4_ | 106.525 | 0.0001 | 55.512 | 157.838 |
| Remission-time interaction β_5_ | -94.710 | 0.0005 | -146.595 | -42.826 |
| Holiday β_6_ | -319.368 | 0.0000 | -408.490 | -230.245 |
| **Telemedicine unit (diagnosed)** | | | | |
| Constant β_0_ | -250.390 | 0.4888 | -965.192 | 464.412 |
| Time(weeks)β_1_ | -9.883 | 0.2912 | -28.359 | 8.594 |
| Seasonality terms β_ck_ and β_sk_ |  |  |  |  |
| Cos1 | 202.439 | 0.4355 | -310.456 | 219.652 |
| Sin1 | 834.278 | 0.0004 | 382.523 | 1286.032 |
| Cos2 | 59.444 | 0.4021 | -80.673 | 199.560 |
| Sin2 | -193.743 | 0.0065 | -332.024 | -55.462 |
| Pandemic outbreak β_2_ | 61.867 | 0.4386 | -95.917 | 219.652 |
| Pandemic remission β_3_ | 50.478 | 0.4987 | -96.974 | 197.930 |
| Outbreak-time interaction β_4_ | 84.402 | 0.0000 | 53.254 | 115.551 |
| Remission-time interaction β_5_ | -35.179 | 0.0289 | -66.674 | -3.684 |
| Holiday β_6_ | -70.072 | 0.0016 | -124.171 | -15.973 |
| **Telemedicine unit (suspected)** | | | | |
| Constant β_0_ | 132.825 | 0.7806 | -810.602 | 1076.251 |
| Time(weeks)β_1_ | -12.610 | 0.3075 | -36.996 | 11.777 |
| Seasonality terms β_ck_ and β_sk_ |  |  |  |  |
| Cos1 | -87.481 | 0.7982 | -764.430 | 589.467 |
| Sin1 | 589.881 | 0.0524 | -6.357 | 1186.119 |
| Cos2 | -0.330 | 0.9972 | -185.257 | 184.592 |
| Sin2 | -75.621 | 0.4131 | -258.128 | 106.886 |
| Pandemic outbreak β_2_ | -188.555 | 0.0755 | -396.809 | 19.698 |
| Pandemic remission β_3_ | 231.154 | 0.0204 | 36.541 | 425.768 |
| Outbreak-time interaction β_4_ | 78.068 | 0.0003 | 36.957 | 119.179 |
| Remission-time interaction β_5_ | -32.113 | 0.1286 | -73.681 | 9.456 |
| Holiday β_6_ | -121.002 | 0.0011 | -192.405 | -49.599 |

Coef., coefficient

CI, confidence interval.

**eTable 3. Poisson segmented regression model of the impact of the COVID-19 pandemic on cancer care utilization related to the top five cancer types in West China Hospital Cancer Center.**

|  | **Breast cancer**  **N=21289** | | | | **Lung cancer**  **N=10550** | | | | **Colorectal cancer**  **N=10529** | | | | **Gastric cancer**  **N=4140** | | | | **Prostate cancer**  **N=102** | | | |
| --- | --- | --- | --- | --- | --- | --- | --- | --- | --- | --- | --- | --- | --- | --- | --- | --- | --- | --- | --- | --- |
| **Inpatient unit(non-surgery)** | **Coef.** | **L 95%CI** | **U 95%CI** | **P** | **Coef.** | **L 95%CI** | **U 95%CI** | **P** | **Coef.** | **L 95%CI** | **U 95%CI** | **P** | **Coef.** | **L 95%CI** | **U 95%CI** | **P** | **Coef.** | **L 95%CI** | **U 95%CI** | **P** |
| Constant β_0_ | 242.237 | 23.772 | 460.702 | 0.0301 | 26.892 | -113.208 | 166.993 | 0.7042 | 33.886 | -112.679 | 180.451 | 0.6475 | -26.899 | -90.199 | 30.421 | 0.4016 | -9.931 | -18.342 | -1.521 | 0.0211 |
| Time(weeks)β_1_ | 1.089 | -4.558 | 6.736 | 0.7029 | 2.574 | -1.047 | 6.196 | 0.1616 | 1.642 | -2.146 | 5.431 | 0.3919 | 1.392 | -0.245 | 3.028 | 0.0947 | 0.254 | 0.036 | 0.471 | 0.0226 |
| Seasonality terms β_ck_ and β_sk_ |  |  |  |  |  |  |  |  |  |  |  |  |  |  |  |  |  |  |  |  |
| Cos1 | 1.139 | -155.618 | 157.897 | 0.9885 | 43.308 | -57.219 | 143.835 | 0.3949 | 41.371 | -63.794 | 146.537 | 0.4371 | 53.551 | 8.124 | 8.978 | 0.0213 | 9.266 | 3.231 | 15.301 | 0.0030 |
| Sin1 | -54.388 | -192.453 | 83.676 | 0.4364 | -25.656 | -114.199 | 62.888 | 0.5668 | 11.077 | -81.552 | 103.706 | 0.8130 | 11.480 | -28.531 | 51.492 | 0.5706 | 0.602 | -4.714 | 5.917 | 0.8228 |
| Cos2 | 1.301 | -41.523 | 44.124 | 0.9521 | 18.037 | -9.426 | 45.500 | 0.1956 | 16.658 | -12.072 | 45.388 | 0.2528 | 11.874 | -0.536 | 24.284 | 0.0606 | 1.461 | -0.188 | 3.110 | 0.0818 |
| Sin2 | 14.547 | -27.716 | 56.810 | 0.4964 | 9.871 | -17.232 | 36.974 | 0.4717 | -1.165 | -29.518 | 27.189 | 0.9352 | -8.386 | -20.634 | 3.861 | 0.1774 | -0.895 | -2.522 | 0.732 | 0.2781 |
| Pandemic outbreak β_2_ | -221.964 | -270.185 | -173.742 | 0.000 | -79.011 | -109.937 | -48.086 | 0.0000 | -95.358 | -127.711 | -63.006 | 0.0000 | -33.766 | -47.741 | -19.791 | 0.0000 | -0.823 | -2.679 | 1.034 | 0.3814 |
| Pandemic remission β_3_ | 19.627 | -25.436 | 64.691 | 0.3897 | 32.249 | 3.349 | 61.149 | 0.0291 | 14.852 | -15.382 | 45.086 | 0.3322 | 9.642 | -3.417 | 22.702 | 0.1462 | 0.741 | -0.994 | 2.476 | 0.3988 |
| Outbreak-time interaction β_4_ | 16.522* | 7.003 | 26.042 | 0.0008 | 3.686 | -2.409 | 9.801 | 0.2327 | 7.503 | 1.116 | 13.889 | 0.0218 | 2.409 | -0.349 | 5.168 | 0.0863 | 0.039 | -0.406 | 0.327 | 0.8322 |
| Remission-time interaction β_5_ | -20.426* | -30.051 | -10.800 | 0.0001 | -7.853 | -14.026 | -1.680 | 0.0132 | -8.821 | -15.279 | -2.363 | 0.0079 | -2.896 | -5.685 | -0.106 | 0.0420 | -0.123 | -0.494 | 0.247 | 0.5101 |
| Holiday β_6_ | -75.443* | -91.976 | -58.909 | 0.0000 | -41.720 | -52.324 | -31.117 | 0.0000 | -45.337 | -56.430 | -34.245 | 0.0000 | -16.441 | -21.233 | -11.650 | 0.0000 | -0.472 | -1.108 | 0.165 | 0.1449 |
| **Inpatient unit(surgery)** | **Coef.** | **L 95%CI** | **U 95%CI** | **P** | **Coef.** | **L 95%CI** | **U 95%CI** | **P** | **Coef.** | **L 95%CI** | **U 95%CI** | **P** | **Coef.** | **L 95%CI** | **U 95%CI** | **P** | **Coef.** | **L 95%CI** | **U 95%CI** | **P** |
| Constant β_0_ | 14.607 | 0.7018 | -60.851 | 0.7018 | 53.012 | -125.895 | 231.919 | 0.5580 | 17.139 | -74.190 | 108.468 | 0.7105 | 22.315 | -27.230 | 71.859 | 0.3738 | 3.282 | -25.526 | 32.090 | 0.8217 |
| Time(weeks)β_1_ | 0.167 | -1.783 | 2.118 | 0.8652 | 0.674 | -3.950 | 5.299 | 0.7730 | 1.184 | -1.177 | 3.545 | 0.3222 | 0.316 | -0.965 | 1.586 | 0.6261 | 0.337 | -0.408 | 1.081 | 0.3722 |
| Seasonality terms β_ck_ and β_sk_ |  |  |  |  |  |  |  |  |  |  |  |  |  |  |  |  |  |  |  |  |
| Cos1 | 15.807 | -38.336 | 69.951 | 0.5638 | 1.663 | -126.709 | 130.035 | 0.9796 | 25.100 | -40.431 | 90.632 | 0.4492 | -1.885 | -37.434 | 33.665 | 0.9165 | 5.705 | -14.965 | 26.376 | 0.5853 |
| Sin1 | 35.280 | -12.410 | 82.969 | 0.1454 | 15.723 | -97.346 | 128.792 | 0.7833 | -11.268 | -68.988 | 46.452 | 0.6994 | -12.701 | -44.013 | 18.611 | 0.4230 | -6.396 | -24.603 | 11.811 | 0.4876 |
| Cos2 | 6.643 | -8.149 | 21.434 | 0.3752 | 7.821 | -27.248 | 42.890 | 0.6592 | 4.448 | -13.454 | 22.351 | 0.6232 | 0.762 | -8.949 | 10.474 | 0.8766 | 0.907 | -4.740 | 6.554 | 0.7507 |
| Sin2 | -8.251 | -22.849 | 6.346 | 0. 2649 | 3.3251 | -31.360 | 37.861 | 0.8526 | 6.498 | -11.170 | 24.165 | 0.4674 | 4.115 | -5.470 | 13.700 | 0.3965 | 0.276 | -5.297 | 5.849 | 0.9220 |
| Pandemic outbreak β_2_ | -55.234* | -71.890 | -38.577 | 0.0000 | -121.866 | -161.357 | -82.375 | 0.0000 | -63.501 | -83.661 | -43.341 | 0.0000 | -26.771 | -37.707 | -15.833 | 0.0000 | -15.465 | -21.824 | -9.106 | 0.0000 |
| Pandemic remission β_3_ | 2.122 | -13.443 | 17.688 | 0.7874 | 22.924 | -13.982 | 59.829 | 0.2208 | 7.500 | -11.339 | 26.340 | 0.4316 | 3.954 | -6.267 | 14.174 | 0.4447 | 0.075 | -5.868 | 6.018 | 0.9800 |
| Outbreak-time interaction β_4_ | 5.399* | 2.111 | 8.687 | 0.0015 | 14.130 | 6.334 | 21.926 | 0.0005 | 7.635 | 3.655 | 11.614 | 0.0002 | 1.895 | -0.264 | 4.054 | 0.0847 | 1.890 | 0.635 | 3.146 | 0.0035 |
| Remission-time interaction β_5_ | -4.017* | -7.342 | -0.692 | 0.0184 | -13.280 | -21.163 | -5.398 | 0.0012 | -9.338 | -13.362 | -5.314 | 0.0000 | -2.650 | -4.833 | -0.467 | 0.0179 | -2.420 | -3.690 | -1.151 | 0.0003 |
| Holiday β_6_ | -21.147* | -26.857 | -15.436 | 0.0000 | -49.930 | -63.470 | -36.390 | 0.0000 | -27.043 | -33.955 | -20.131 | 0.0000 | -10.780 | -14.530 | -7.031 | 0.0000 | -5.199 | -7.379 | -3.018 | 0.0000 |
| **Outpatient physician visit** | **Coef.** | **L 95%CI** | **U 95%CI** | **P** | **Coef.** | **L 95%CI** | **U 95%CI** | **P** | **Coef.** | **L 95%CI** | **U 95%CI** | **P** | **Coef.** | **L 95%CI** | **U 95%CI** | **P** | **Coef.** | **L 95%CI** | **U 95%CI** | **P** |
| Constant β_0_ | 654.962 | -218.869 | 1528.793 | 0.1402 | 652.366 | -9.223 | 1313.954 | 0.0532 | -13.514 | -416.063 | 389.035 | 0.9470 | 44.858 | -124.797 | 214.513 | 0.6011 | -12.819 | -108.692 | 83.055 | 0.7914 |
| Time(weeks)β_1_ | 18.788 | -3.798 | 41.375 | 0.1020 | 3.810 | 20.911 | 20.911 | 0.6595 | 10.424 | 0.019 | 20.829 | 0.0496 | 3.169 | -1.216 | 7.555 | 0.1548 | 1.595 | 4.073 | 4.073 | -0.883 |
| Seasonality terms β_ck_ and β_sk_ |  |  |  |  |  |  |  |  |  |  |  |  |  |  |  |  |  |  |  |  |
| Cos1 | 300.894 | -326.109 | 927.897 | 0.3435 | -36.460 | -511.171 | 438.251 | 0.8792 | 208.519 | -80.324 | 497.361 | 0.1552 | 58.094 | -63.639 | 179.827 | 0.3461 | 57.968 | -10.825 | 126.760 | 0.0977 |
| Sin1 | -395.578 | -947.837 | 156.681 | 0.1585 | -98.110 | -516.237 | 320.017 | 0.6427 | -28.675 | -283.094 | 225.744 | 0.8236 | -22.881 | -130.102 | 84.340 | 0.6730 | 23.488 | -37.104 | 84.080 | 0.4438 |
| Cos2 | 30.376 | -140.914 | 201.666 | 0.7258 | 1.741 | -127.944 | 131.426 | 0.9788 | 54.393 | -24.526 | 133.301 | 0.1746 | 13.250 | -20.006 | 46.505 | 0.4313 | 13.992 | -4.801 | 32.786 | 0.1428 |
| Sin2 | 25.715 | -143.330 | 194.761 | 0.7635 | -25.411 | -153.398 | 102.577 | 0.6946 | -25.659 | -103.535 | 52.218 | 0.5149 | -9.885 | -42.706 | 22.935 | 0.5516 | -5.368 | -23.915 | 13.179 | 0.5672 |
| Pandemic outbreak β_2_ | -1086.978* | -1278.866 | -893.091 | 0.0000 | -572.324 | -718.362 | -426.287 | 0.0000 | -360.596 | -449.457 | -271.735 | 0.0000 | -155.611 | -193.060 | -118.162 | 0.0000 | -47.504 | -68.667 | -26.341 | 0.0000 |
| Pandemic remission β_3_ | -42.129 | -222.388 | 138.127 | 0.6440 | 15.516 | -120.959 | 151.990 | 0.8221 | 23.956 | -59.086 | 106.998 | 0.5685 | 1.780 | -33.217 | 36.777 | 0.9199 | 1.882 | -17.895 | 21.659 | 0.8507 |
| Outbreak-time interaction β_4_ | 88.818* | 50.741 | 126.896 | 0.0000 | 43.791 | 14.962 | 72.620 | 0.0033 | 26.096 | 8.555 | 43.638 | 0.0039 | 14.555 | 7.163 | 21.948 | 0.0002 | 3.549 | -0.628 | 7.727 | 0.0950 |
| Remission-time interaction β_5_ | -120.497 | -158.999 | -81.995 | 0.0000 | -47.561 | -76.711 | -18.410 | 0.0016 | -36.392 | -54.130 | -18.655 | 0.0001 | -18.239 | -25.714 | -10.764 | 0.0000 | -4.244 | -8.468 | -0.020 | 0.0490 |
| Holiday β_6_ | -326.655 | -392.789 | -260.521 | 0.0000 | -210.803 | -260.874 | -160.732 | 0.0000 | -99.970 | -130.437 | -69.503 | 0.0000 | -46.142 | -58.982 | -33.302 | 0.0000 | -27.511 | -34.767 | -20.255 | 0.0000 |
| **Outpatient chemotherapy** | **Coef.** | **L 95%CI** | **U 95%CI** | **P** | **Coef.** | **L 95%CI** | **U 95%CI** | **P** | **Coef.** | **L 95%CI** | **U 95%CI** | **P** | **Coef.** | **L 95%CI** | **U 95%CI** | **P** | **Coef.** | **L 95%CI** | **U 95%CI** | **P** |
| Constant β_0_ | 106.847 | -206.755 | 420.449 | 0.5007 | -63.265 | -139.893 | 13.362 | 0.1046 | 52.176 | 4.458 | 99.894 | 0.0324 | 21.516 | 9.637 | 33.395 | 0.0005 | -0.595 | -10.488 | 9.298 | 0.9053 |
| Time(weeks)β_1_ | 6.479 | -1.627 | 14.585 | 0.1160 | 2.675 | 0.694 | 4.656 | 0.0086 | -0.854 | -2.087 | 0.380 | 0.1729 | -0.451 | -0.758 | -0.144 | 0.0044 | 0.140 | -0.116 | 0.395 | 0.2816 |
| Seasonality terms β_ck_ and β_sk_ |  |  |  |  |  |  |  |  |  |  |  |  |  |  |  |  |  |  |  |  |
| Cos1 | 57.587 | -167.433 | 282.607 | 0.6128 | 69.551 | 14.568 | 124.534 | 0.0137 | -10.378 | -44.617 | 23.862 | 0.5491 | -14.292 | -22.816 | -5.769 | 0.0012 | 4.318 | -2.781 | 11.416 | 0.2304 |
| Sin1 | -128.176 | -326.372 | 70.020 | 0.2025 | 8.257 | -40.172 | 56.685 | 0.7360 | 10.961 | -19.197 | 41.119 | 0.4726 | -0.661 | -8.168 | 6.847 | 0.8618 | -3.688 | -9.940 | 2.565 | 0.2448 |
| Cos2 | 22.685 | -38.787 | 84.158 | 0.4659 | 18.472 | 3.452 | 33.493 | 0.0164 | -10.359 | -19.713 | -1.006 | 0.0303 | -3.998 | -6.326 | -1.669 | 0.0009 | 0.220 | -1.719 | 2.159 | 0.8225 |
| Sin2 | 25.319 | -35.349 | 85.986 | 0.4098 | -7.883 | -22.707 | 6.941 | 0.2941 | -1.928 | -11.159 | 7.303 | 0.6795 | 1.129 | -1.169 | 3.427 | 0.3322 | -1.237 | -3.151 | 0.677 | 0.2027 |
| Pandemic outbreak β_2_ | -181.966 | -251.190 | -112.743 | 0.0000 | -37.029 | -53.943 | -20.114 | 0.0000 | -11.619 | -22.152 | -1.086 | 0.0310 | 0.172 | -2.450 | 2.794 | 0.8969 | -0.202 | -2.386 | 1.981 | 0.8545 |
| Pandemic remission β_3_ | -11.450 | -76.141 | 53.241 | 0.7263 | -2.018 | -17.825 | 13.789 | 0.8006 | 4.894 | -4.950 | 14.737 | 0.3264 | 0.882 | -1.569 | 3.332 | 0.4770 | 1.144 | -0.896 | 3.185 | 0.2686 |
| Outbreak-time interaction β_4_ | 11.2172 | -2.493 | 24.837 | 0.1080 | 0.639 | -2.700 | 3.978 | 0.7050 | 0.755 | -1.325 | 2.834 | 0.4733 | 0.029 | -0.489 | 0.546 | 0.9131 | -0.222 | -0.653 | 0.209 | 0.3099 |
| Remission-time interaction β_5_ | -22.419 | -36.787 | -8.601 | 0.0017 | -2.601 | -5.977 | 0.776 | 0.1297 | 0.535 | -1.568 | 2.637 | 0.6149 | 0.365 | -0.158 | 0.889 | 0.1695 | 0.010 | -0.426 | 0.446 | 0.9628 |
| Holiday β_6_ | -67.381 | -91.115 | -43.646 | 0.0000 | -14.952 | -20.751 | -9.153 | 0.0000 | -8.623 | -12.234 | -5.011 | 0.0000 | -1.382 | -2.281 | -0.483 | 0.0029 | -0.164 | -0.913 | 0.584 | 0.6643 |
| **Outpatient radiotherapy** | **Coef.** | **L 95%CI** | **U 95%CI** | **P** | **Coef.** | **L 95%CI** | **U 95%CI** | **P** | **Coef.** | **L 95%CI** | **U 95%CI** | **P** | **Coef.** | **L 95%CI** | **U 95%CI** | **P** | **Coef.** | **L 95%CI** | **U 95%CI** | **P** |
| Constant β_0_ | 43.139 | -7.600 | 93.878 | 0.0948 | 52.045 | -14.573 | 118.663 | 0.1243 | 5.151 | -28.276 | 38.579 | 0.7605 | 0.690 | -14.245 | 12.864 | 0.9197 | 4.430 | -15.112 | 23.971 | 0.6540 |
| Time(weeks)β_1_ | -0.431 | -1.742 | 0.881 | 0.5164 | -0.576 | -2.298 | 1.146 | 0.5083 | -0.205 | -1.069 | 0.659 | 0.6396 | 0.064 | -0.286 | 0.415 | 0.7173 | -0.023 | -0.528 | 0.482 | 0.9275 |
| Seasonality terms β_ck_ and β_sk_ |  |  |  |  |  |  |  |  |  |  |  |  |  |  |  |  |  |  |  |  |
| Cos1 | -10.657 | -42.064 | 25.750 | 0.5628 | -28.668 | -76.649 | 19.133 | 0.2370 | 4.668 | -19.317 | 28.653 | 0.7003 | 3.031 | -6.695 | 12.757 | 0.5379 | -0.977 | -14.998 | 13.045 | 0.8904 |
| Sin1 | -4.534 | -36.601 | 27.533 | 0.7797 | 8.684 | -33.419 | 50.787 | 0.6833 | 17.066 | -4.060 | 38.192 | 0.1222 | 0.513 | -8.053 | 9.080 | 0.9057 | 0.751 | -11.599 | 13.101 | 0.9042 |
| Cos2 | -9.210 | -19.158 | 0.736 | 0.0692 | -2.201 | -15.260 | 10.857 | 0.7388 | 1.685 | -4.868 | 8.237 | 0.6112 | 0.104 | -2.553 | 2.761 | 0.9384 | 0.234 | -3.596 | 4.065 | 0.9036 |
| Sin2 | -1.679 | -11.495 | 8.137 | 0.7351 | 7.674 | -5.213 | 20.562 | 0.2403 | -4.495 | -10.961 | 1.972 | 0.1710 | -1.144 | -3.766 | 1.478 | 0.3888 | 1.792 | -1.988 | 5.572 | 0.3494 |
| Pandemic outbreak β_2_ | -15.035 | -26.235 | -3.835 | 0.0090 | -20.114 | -34.819 | -5.409 | 0.0078 | -4.270 | -11.649 | 3.108 | 0.2537 | -1.971 | -3.963 | 1.021 | 0.1943 | -0.260 | -4.574 | 4.054 | 0.9051 |
| Pandemic remission β_3_ | 2.996 | -7.471 | 13.463 | 0.5715 | 1.213 | -12.530 | 14.955 | 0.8614 | -0.078 | -6.973 | 6.818 | 0.9822 | -2.117 | -4.913 | 0.680 | 0.1363 | 3.523 | -0.508 | 7.554 | 0.0861 |
| Outbreak-time interaction β_4_ | 2.024 | -0.187 | 4.235 | 0.0724 | 2.795 | -0.107 | 5.698 | 0.0589 | 0.651 | -0.805 | 2.108 | 0.3771 | 0.355 | -0.236 | 0.945 | 0.2364 | 0.088 | -0.763 | 0.940 | 0.8372 |
| Remission-time interaction β_5_ | -1.612 | -3.848 | 0.623 | 0.1556 | -2.136 | -5.071 | 0.799 | 0.1520 | 0.342 | -1.131 | 1.814 | 0.6465 | -0.310 | -0.907 | 0.288 | 0.3064 | -0.172 | -1.033 | 0.689 | 0.6921 |
| Holiday β_6_ | -13.197 | -17.037 | -9.357 | 0.0000 | -20.290 | -25.332 | -15.249 | 0.0000 | -7.239 | -9.769 | -4.709 | 0.0000 | -1.253 | -2.279 | -0.228 | 0.0171 | -1.850 | -3.329 | -0.371 | 0.0147 |
| **Pharmacy and Laboratory** | **Coef.** | **L 95%CI** | **U 95%CI** | **P** | **Coef.** | **L 95%CI** | **U 95%CI** | **P** | **Coef.** | **L 95%CI** | **U 95%CI** | **P** | **Coef.** | **L 95%CI** | **U 95%CI** | **P** | **Coef.** | **L 95%CI** | **U 95%CI** | **P** |
| Constant β_0_ | 642.790 | -619.296 | 1904.877 | 0.3148 | 29.982 | -77.808 | 137.773 | 0.5824 | 61.001 | -26.194 | 148.196 | 0.1693 | 19.541 | -7.964 | 47.047 | 0.1618 | -5.673 | -22.147 | 10.801 | 0.4962 |
| Time(weeks)β_1_ | 12.330 | -20.293 | 44.935 | 0.4552 | 1.371 | -1.415 | 4.157 | 0.3314 | -0.731 | -2.984 | 1.523 | 0.5217 | -0.141 | -0.852 | 0.570 | 0.6952 | 0.317 | -0.109 | 0.742 | -0.109 |
| Seasonality terms β_ck_ and β_sk_ |  |  |  |  |  |  |  |  |  |  |  |  |  |  |  |  |  |  |  |  |
| Cos1 | 97.747 | -807.843 | 1003.336 | 0.8309 | 2.571 | -74.772 | 79.914 | 0.9476 | -8.249 | -70.814 | 54.316 | 0.7942 | -9.519 | -29.255 | 10.217 | 0.3410 | 8.930 | -2.891 | 20.751 | 0.1371 |
| Sin1 | -89.523 | -887.159 | 708.114 | 0.8243 | -7.091 | -75.214 | 61.033 | 0.8369 | 33.050 | -22.060 | 88.160 | 0.2370 | -2.720 | -20.103 | 14.664 | 0.7570 | -3.131 | -13.542 | 7.281 | 0.5523 |
| Cos2 | 55.950 | -191.447 | 303.347 | 0.6547 | 2.783 | -18.346 | 23.913 | 0.7944 | -12.873 | -29.965 | 4.219 | 0.1383 | -2.896 | -8.288 | 2.495 | 0.2892 | 0.528 | -2.701 | 3.758 | 0.7463 |
| Sin2 | 27.404 | -216.750 | 271.559 | 0.8243 | 14.180 | -6.672 | 35.033 | 0.1804 | -0.073 | -16.941 | 16.796 | 0.9932 | 0.055 | -5.266 | 5.376 | 0.9837 | -1.407 | -4.594 | 1.780 | 0.3832 |
| Pandemic outbreak β_2_ | -786.4* | -1064.976 | -507.792 | 0.0000 | -39.723 | -63.517 | -15.930 | 0.0013 | -57.490 | -76.738 | -38.242 | 0.0000 | -9.682 | -15.753 | -3.610 | 0.0021 | -3.874 | -7.510 | -0.237 | 0.0371 |
| Pandemic remission β_3_ | 112.3 | -148.051 | 372.643 | 0.3943 | -7.105 | -29.341 | 15.130 | 0.5276 | -2.507 | -20.494 | 15.480 | 0.7827 | -2.881 | -8.555 | 2.793 | 0.3163 | 1.068 | -2.330 | 4.467 | 0.5344 |
| Outbreak-time interaction β_4_ | 32.4 | -22.550 | 87.443 | 0.2447 | 1.959 | -2.738 | 6.656 | 0.4101 | 7.275 | 3.476 | 11.075 | 0.0002 | 0.384 | -0.815 | 1.582 | 0.5270 | 0.146 | -0.572 | 0.863 | 0.6833 |
| Remission-time interaction β_5_ | -52.2 | -107.851 | 3.367 | 0.0653 | -4.433 | -9.182 | 0.316 | 0.0670 | -5.853 | -9.695 | -2.011 | 0.0032 | -0.351 | -1.563 | 0.861 | 0.5669 | -0.533 | -1.258 | 0.193 | 0.1487 |
| Holiday β_6_ | -287.2* | -382.756 | -191.717 | 0.0000 | -21.473 | -29.631 | -13.315 | 0.0000 | -16.697 | -23.296 | -10.098 | 0.0000 | -3.730 | -5.812 | -1.649 | 0.0006 | -1.628 | -2.874 | -0.381 | 0.0110 |
| **Telemedicine(diagnosed)** | **Coef.** | **L 95%CI** | **U 95%CI** | **P** | **Coef.** | **L 95%CI** | **U 95%CI** | **P** | **Coef.** | **L 95%CI** | **U 95%CI** | **P** | **Coef.** | **L 95%CI** | **U 95%CI** | **P** | **Coef.** | **L 95%CI** | **U 95%CI** | **P** |
| Constant β_0_ | 31.838 | -119.457 | 183.133 | 0.6773 | -81.984 | -262.881 | 98.912 | 0.3708 | -26.262 | -90.442 | 37.919 | 0.4189 | -22.486 | -50.908 | 5.935 | 0.1197 | -3.514 | -22.351 | 15.323 | 0.7122 |
| Time(weeks)β_1_ | 0.604 | -3.307 | 4.514 | 0.7602 | -2.484 | -7.159 | 2.192 | 0.2946 | -0.190 | -1.849 | 1.469 | 0.8208 | -0.069 | -0.804 | 0.665 | 0.8520 | -0.167 | -0.654 | 0.320 | 0.4982 |
| Seasonality terms β_ck_ and β_sk_ |  |  |  |  |  |  |  |  |  |  |  |  |  |  |  |  |  |  |  |  |
| Cos1 | -11.398 | -119.957 | 97.161 | 0.8355 | 43.718 | -86.081 | 173.516 | 0.5056 | 33.069 | -12.983 | 79.121 | 0.1574 | 21.582 | 1.189 | 41.976 | 0.0383 | 3.872 | -9.645 | 17.388 | 0.5712 |
| Sin1 | -70.689 | -166.308 | 24.930 | 0.1456 | 235.873 | 121.543 | 350.203 | 0.0001 | 35.991 | -4.572 | 76.553 | 0.0814 | 29.960 | 11.998 | 47.922 | 0.0013 | 12.201 | 0.295 | 24.106 | 0.0447 |
| Cos2 | -18.664 | -48.322 | 10.993 | 0.2148 | 32.944 | -2.515 | 68.403 | 0.0683 | -3.846 | -16.427 | 8.734 | 0.5456 | 1.992 | -3.579 | 7.564 | 0.4797 | 0.522 | -3.170 | 4.215 | 0.7797 |
| Sin2 | 25.157 | -4.111 | 54.426 | 0.0913 | -54.628 | -89.628 | -19.627 | 0.0025 | -15.098 | -27.514 | -2.682 | 0.0176 | -10.693 | -16.191 | -5.195 | 0.0002 | -3.694 | -7.338 | -0.050 | 0.0470 |
| Pandemic outbreak β_2_ | 50.729 | 17.332 | 84.126 | 0.0033 | 4.449 | -35.481 | 44.379 | 0.8256 | 1.449 | -12.718 | 15.616 | 0.8397 | -0.364 | -6.638 | 5.910 | 0.9086 | 7.086 | 2.928 | 11.244 | 0.0010 |
| Pandemic remission β_3_ | 13.563 | -17.647 | 44.772 | 0.3908 | 0.067 | -37.253 | 37.387 | 0.9972 | 14.095 | 0.855 | 27.334 | 0.0372 | 1.690 | -4.172 | 7.553 | 0.5687 | 3.322 | -0.564 | 7.208 | 0.0930 |
| Outbreak-time interaction β_4_ | 7.781 | 1.188 | 14.374 | 0.0212 | 15.774 | 7.891 | 23.657 | 0.0001 | 4.696 | 1.899 | 7.493 | 0.0012 | 1.845 | 0.606 | 3.083 | 0.0039 | 0.074 | -0.747 | 0.895 | 0.8582 |
| Remission-time interaction β_5_ | -11.799 | -18.466 | -5.133 | 0.0007 | -0.754 | -8.724 | 7.216 | 0.8515 | -2.520 | -5.348 | 0.307 | 0.0801 | -0.132 | -1.385 | 1.120 | 0.8342 | 0.538 | -0.292 | 1.368 | 0.2014 |
| Holiday β_6_ | -6.480 | -17.931 | 4.970 | 0.2643 | -16.807 | -30.498 | -3.117 | 0.0166 | -2.134 | -6.992 | 2.723 | 0.3855 | -1.853 | -4.004 | 0.298 | 0.0905 | -0.887 | -2.312 | 0.539 | 0.2202 |

**eTable 4. Changes in cancer care utilizations in West China Hospital Cancer Center, stratified by service type and sex.**

|  | **Coef.** | **P value** | **95%CI(Lower)** | **95%CI(Upper)** | **Coef.** | **P value** | **95%CI(Lower)** | **95%CI(Upper)** |
| --- | --- | --- | --- | --- | --- | --- | --- | --- |
|  | **Men** | | | | **Women** | | | |
| **Inpatient unit (non-surgery)** | | | | | | | | |
| Constant β_0_ | 21.768 | 0.8967 | -308.879 | 353.414 | 276.059 | 0.1340 | -86.425 | 638.543 |
| Time(weeks)β_1_ | 5.232 | 0.2289 | -3.341 | 13.804 | 3.883 | 0.4130 | -5.486 | 13.253 |
| Seasonality terms β_ck_ and β_sk_ |  |  |  |  |  |  |  |  |
| Cos1 | 158.452 | 0.1896 | -79.515 | 396.419 | 73.513 | 0.5763 | -186.581 | 333.607 |
| Sin1 | 42.599 | 0.6877 | -167.002 | 252.200 | -25.149 | 0.8281 | -254.238 | 203.940 |
| Cos2 | 43.918 | 0.1833 | -21.092 | 108.929 | 34.176 | 0.3424 | -36.878 | 105.231 |
| Sin2 | -4.764 | 0.8832 | -68.922 | 59.395 | 11.271 | 0.7505 | -58.853 | 81.395 |
| Pandemic outbreak β_2_ | -223.254 | 0.0000 | -296.461 | -150.046 | -361.168 | 0.0000 | -441.182 | -281.155 |
| Pandemic remission β_3_ | 60.410 | 0.0829 | -8.004 | 128.823 | 48.144 | 0.2045 | -26.630 | 122.918 |
| Outbreak-time interaction β_4_ | 18.693 | 0.0117 | 4.242 | 33.145 | 25.306 | 0.0020 | 9.511 | 41.102 |
| Remission-time interaction β_5_ | -22.183 | 0.0033 | -36.796 | -7.570 | -30.850 | 0.0002 | -46.821 | -14.879 |
| Holiday β_6_ | -114.639 | 0.0000 | -139.739 | -89.539 | -143.505 | 0.0000 | -170.939 | -116.071 |
| **Inpatient unit (surgery)** | | | | | | | | |
| Constant β_0_ | -24.836 | 0.9056 | -439.115 | 389.443 | 111.527 | 0.6330 | -350.281 | 573.335 |
| Time(weeks)β_1_ | 4.504 | 0.4061 | -6.204 | 15.212 | 3.501 | 0.5620 | -8.436 | 15.438 |
| Seasonality terms β_ck_ and β_sk_ |  |  |  |  |  |  |  |  |
| Cos1 | 191.410 | 0.2045 | -105.850 | 488.669 | 68.789 | 0.6814 | -262.574 | 400.152 |
| Sin1 | 187.894 | 0.1566 | -73.930 | 449.717 | 103.706 | 0.4826 | -188.157 | 395.569 |
| Cos2 | 64.474 | 0.1184 | -16.734 | 145.681 | 56.639 | 0.2175 | -33.886 | 147.163 |
| Sin2 | -41.033 | 0.3123 | -121.177 | 39.110 | -8.073 | 0.8581 | -97.411 | 81.266 |
| Pandemic outbreak β_2_ | -349.933 | 0.0000 | -441.380 | -258.486 | -363.153 | 0.0000 | -465.092 | -261.214 |
| Pandemic remission β_3_ | 33.826 | 0.4343 | -51.633 | 119.284 | 67.523 | 0.1628 | -27.741 | 162.786 |
| Outbreak-time interaction β_4_ | 38.732 | 0.0000 | 20.679 | 56.784 | 30.307 | 0.0035 | 10.183 | 50.430 |
| Remission-time interaction β_5_ | -34.050 | 0.0003 | -52.303 | -15.796 | -28.059 | 0.0073 | -48.407 | -7.711 |
| Holiday β_6_ | -139.226 | 0.0000 | -170.580 | -107.872 | -157.123 | 0.0000 | -192.074 | -122.171 |
| **Outpatient physician visit unit (diagnosed)** | | | | | | | | |
| Constant β_0_ | 334.014 | 0.6441 | -1095.600 | 1763.629 | 842.107 | 0.3635 | -987.583 | 2671.797 |
| Time(weeks)β_1_ | 33.462 | 0.0754 | -3.491 | 70.414 | 43.032 | 0.0741 | -4.263 | 90.237 |
| Seasonality terms β_ck_ and β_sk_ |  |  |  |  |  |  |  |  |
| Cos1 | 725.053 | 0.1640 | -300.742 | 1750.849 | 874.100 | 0.1896 | -438.766 | 2186.955 |
| Sin1 | -7.094 | 0.9876 | -910.598 | 896.410 | -262.213 | 0.6538 | -1418.531 | 894.105 |
| Cos2 | 203.478 | 0.1529 | -76.760 | 483.715 | 222.040 | 0.2223 | -136.619 | 580.698 |
| Sin2 | -69.122 | 0.6212 | -345.689 | 207.444 | -56.509 | 0.7522 | -410.454 | 297.435 |
| Pandemic outbreak β_2_ | -1457.377 | 0.0000 | -1772.947 | -1141.807 | -2230.155 | 0.0000 | -2634.041 | -1826.270 |
| Pandemic remission β_3_ | 41.829 | 0.7790 | -253.072 | 336.730 | -3.283 | 0.9863 | -380.686 | 374.120 |
| Outbreak-time interaction β_4_ | 127.975 | 0.0001 | 65.679 | 190.270 | 181.849 | 0.0000 | 102.120 | 261.579 |
| Remission-time interaction β_5_ | -157.342 | 0.0000 | -220.334 | -94.351 | -228.748 | 0.0000 | -209.368 | -148.129 |
| Holiday β_6_ | -504.080 | 0.0000 | -612.278 | -395.882 | -702.766 | 0.0000 | -841.242 | -564.289 |
| **Outpatient physician visit unit (suspected)** | | | | | | | | |
| Constant β_0_ | 92.601 | 0.4218 | -135.125 | 320.327 | 154.478 | 0.5290 | -330.525 | 639.481 |
| Time(weeks)β_1_ | 0.487 | 0.8699 | -5.399 | 6.374 | 4.202 | 0.5077 | -8.335 | 16.738 |
| Seasonality terms β_ck_ and β_sk_ |  |  |  |  |  |  |  |  |
| Cos1 | 63.767 | 0.4407 | -99.634 | 227.168 | 202.242 | 0.2518 | -145.764 | 550.247 |
| Sin1 | 31.599 | 0.6642 | -112.325 | 175.522 | 3.088 | 0.9841 | -303.441 | 309.617 |
| Cos2 | 31.051 | 0.1707 | -13.588 | 75.690 | 64.954 | 0.1784 | -30.117 | 160.026 |
| Sin2 | -41.483 | 0.0647 | -85.538 | 2.571 | -118.202 | 0.0141 | -212.028 | -24.376 |
| Pandemic outbreak β_2_ | -126.932 | 0.0000 | -177.200 | -76.663 | -346.670 | 0.0000 | -453.729 | -239.611 |
| Pandemic remission β_3_ | -18.135 | 0.4456 | -65.111 | 28.841 | 16.791 | 0.7399 | -83.257 | 116.839 |
| Outbreak-time interaction β_4_ | 12.109 | 0.0173 | 2.186 | 22.033 | 16.099 | 0.1339 | -5.036 | 37.233 |
| Remission-time interaction β_5_ | -9.129 | 0.0741 | -19.163 | 0.905 | -12.404 | 0.2523 | -33.774 | 8.966 |
| Holiday β_6_ | -44.423 | 0.0000 | -61.658 | -27.188 | -103.561 | 0.0000 | -140.268 | -66.855 |
| **Outpatient chemotherapy unit** | | | | | | | | |
| Constant β_0_ | 32.996 | 0.5174 | -67.750 | 133.742 | 100.676 | 0.5653 | -243.878 | 445.231 |
| Time(weeks)β_1_ | -0.373 | 0.7771 | -2.977 | 2.231 | 7.830 | 0.0842 | -1.076 | 16.736 |
| Seasonality terms β_ck_ and β_sk_ |  |  |  |  |  |  |  |  |
| Cos1 | 27.710 | 0.4489 | -44.579 | 99.998 | 100.924 | 0.4200 | -146.306 | 348.154 |
| Sin1 | 65.677 | 0.0433 | 2.006 | 129.348 | -116.934 | 0.2894 | -334.692 | 100.824 |
| Cos2 | 0.787 | 0.79731 | -18.961 | 20.535 | 34.863 | 0.3084 | -32.677 | 102.404 |
| Sin2 | -10.644 | 0.2813 | -30.133 | 8.846 | 18.841 | 0.5763 | -47.815 | 85.496 |
| Pandemic outbreak β_2_ | -39.072 | 0.0007 | -61.310 | -16.833 | -258.484 | 0.0000 | -334.539 | -182.426 |
| Pandemic remission β_3_ | -4.217 | 0.6882 | -24.999 | 16.565 | 16.942 | 0.6374 | -54.133 | 88.018 |
| Outbreak-time interaction β_4_ | 3.842 | 0.0856 | -0.548 | 8.232 | 10.874 | 0.1539 | -4.141 | 25.888 |
| Remission-time interaction β_5_ | -0.793 | 0.7239 | -5.232 | 3.646 | -22.741 | 0.0037 | -37.922 | -7.559 |
| Holiday β_6_ | -20.603 | 0.0000 | -28.227 | -12.978 | -80.117 | 0.0000 | -106.194 | -54.040 |
| **Outpatient radiotherapy unit** | | | | | | | | |
| Constant β_0_ | 83.323 | 0.1723 | -36.914 | 203.561 | -4.597 | 0.9343 | -114.926 | 105.733 |
| Time(weeks)β_1_ | -.0777 | 0.6209 | -3.885 | 2.331 | 1.614 | 0.2642 | -1.237 | 4.466 |
| Seasonality terms β_ck_ and β_sk_ |  |  |  |  |  |  |  |  |
| Cos1 | -16.932 | 0.6979 | -103.206 | 69.343 | 55.455 | 0.1678 | -23.720 | 134.610 |
| Sin1 | 36.092 | 0.3484 | -39.898 | 112.082 | 22.752 | 0.5190 | -46.975 | 92.480 |
| Cos2 | 0.948 | 0.9366 | -22.621 | 24.517 | 15.598 | 0.1556 | -6.029 | 37.225 |
| Sin2 | 7.412 | 0.5288 | -15.849 | 30.672 | -3.608 | 0.7381 | -24.951 | 17.736 |
| Pandemic outbreak β_2_ | -23.699 | 0.0795 | -50.240 | 2.842 | -40.366 | 0.0014 | -64.720 | -16.012 |
| Pandemic remission β_3_ | 10.617 | 0.3979 | -14.186 | 35.420 | 6.058 | 0.5987 | -16.702 | 28.816 |
| Outbreak-time interaction β_4_ | 3.607 | 0.1751 | -1.632 | 8.847 | 3.184 | 0.1920 | -1.624 | 7.991 |
| Remission-time interaction β_5_ | -1.716 | 0.5220 | -7.014 | 3.582 | -3.767 | 0.1274 | -8.628 | 1.094 |
| Holiday β_6_ | -47.325 | 0.0000 | -56.425 | -38.225 | -42.355 | 0.0000 | -50.705 | -34.005 |
| **Pharmacy and Laboratory unit** | | | | | | | | |
| Constant β_0_ | -91.896 | 0.5218 | -375.409 | 191.616 | 1014.477 | 0.0614 | -49.352 | 2078.307 |
| Time(weeks)β_1_ | 6.699 | 0.0727 | -0.629 | 14.028 | 6.352 | 0.6478 | -21.146 | 33.850 |
| Seasonality terms β_ck_ and β_sk_ |  |  |  |  |  |  |  |  |
| Cos1 | 196.882 | 0.0577 | -6.547 | 400.312 | -63.154 | 0.8700 | -826.489 | 700.181 |
| Sin1 | 30.437 | 0.7369 | -148.744 | 209.617 | -130.023 | 0.7021 | -802.358 | 542.312 |
| Cos2 | 31.147 | 0.2689 | -24.428 | 86.721 | -7.917 | 0.9401 | -216.451 | 200.616 |
| Sin2 | -16.891 | 0.5427 | -71.737 | 37.956 | 47.325 | 0.6493 | -158.476 | 253.125 |
| Pandemic outbreak β_2_ | -134.623 | 0.0000 | -197.205 | -72.040 | -1032.962 | 0.0000 | -1267.790 | -798.134 |
| Pandemic remission β_3_ | -47.364 | 0.1113 | -105.848 | 11.120 | 47.470 | 0.6688 | -171.979 | 266.919 |
| Outbreak-time interaction β_4_ | 16.305 | 0.0102 | 3.951 | 28.660 | 80.252 | 0.0009 | 33.895 | -49.552 |
| Remission-time interaction β_5_ | -21.621 | 0.0009 | -34.113 | -9.129 | -96.425 | 0.0001 | -143.298 | -49.552 |
| Holiday β_6_ | -125.717 | 0.0000 | -147.174 | -104.260 | -308.774 | 0.0000 | -389.288 | -228.260 |
| **Telemedicine(diagnosed)** | | | | | | | | |
| Constant β_0_ | -175.885 | 0.2656 | -487.545 | 135.776 | -73.251 | 0.7364 | -503.636 | 357.135 |
| Time(weeks)β_1_ | -4.850 | 0.2352 | -12.906 | 3.205 | -5.084 | 0.3669 | -16.208 | 6.041 |
| Seasonality terms β_ck_ and β_sk_ |  |  |  |  |  |  |  |  |
| Cos1 | 141.158 | 0.2134 | -82.469 | 364.785 | 60.383 | 0.6990 | -248.433 | 369.200 |
| Sin1 | 467.058 | 0.0000 | 270.089 | 664.028 | 367.914 | 0.0085 | 95.913 | 639.916 |
| Cos2 | 38.175 | 0.2181 | -22.917 | 99.267 | 20.997 | 0.6226 | -63.368 | 105.361 |
| Sin2 | -121.879 | 0.0001 | -182.170 | -61.587 | -71.815 | 0.0902 | -155.074 | 11.455 |
| Pandemic outbreak β_2_ | 31.425 | 0.3671 | -37.370 | 100.220 | 30.820 | 0.5214 | -64.181 | 125.822 |
| Pandemic remission β_3_ | 14.417 | 0.6574 | -49.874 | 78.707 | 36.009 | 0.4230 | -52.771 | 124.789 |
| Outbreak-time interaction β_4_ | 35.516 | 0.0000 | 21.935 | 49.097 | 48.938 | 0.0000 | 30.184 | 67.692 |
| Remission-time interaction β_5_ | -8.016 | 0.2497 | -21.748 | 5.716 | -27.143 | 0.0055 | -46.106 | -8.180 |
| Holiday β_6_ | -31.996 | 0.0083 | -55.583 | -8.048 | -38.039 | 0.0225 | -70.612 | -5.466 |
| **Telemedicine(suspected)** | | | | | | | | |
| Constant β_0_ | 139.491 | 0.3111 | -132.272 | 411.255 | -6.082 | 0.9862 | -703.575 | 691.410 |
| Time(weeks)β_1_ | -1.583 | 0.6560 | -8.607 | 5.442 | -11.051 | 0.2669 | -29.081 | 6.980 |
| Seasonality terms β_ck_ and β_sk_ |  |  |  |  |  |  |  |  |
| Cos1 | -106.877 | 0,2796 | -301.877 | 88.122 | 18.977 | 0.9402 | -481.485 | 519.440 |
| Sin1 | -56.720 | 0.5140 | -228.474 | 115.034 | 646.900 | 0.0044 | 206.005 | 1087.796 |
| Cos2 | -19.180 | 0.4768 | -72.452 | 34.091 | 18.724 | 0.7865 | -118.008 | 155.456 |
| Sin2 | 35.527 | 0.1831 | -17.046 | 88.101 | -111.118 | 0.1055 | -246.061 | 23.825 |
| Pandemic outbreak β_2_ | -50.590 | 0.0975 | -110.578 | 9.399 | -137.787 | 0.0789 | -291.782 | 16.209 |
| Pandemic remission β_3_ | 51.302 | 0.0724 | -4.758 | 107.362 | 179.832 | 0.0148 | 35.921 | 323.743 |
| Outbreak-time interaction β_4_ | 17.985 | 0.0033 | 6.143 | 29.827 | 60.106 | 0.0002 | 29.706 | 90.506 |
| Remission-time interaction β_5_ | -15.677 | 0.0108 | -27.651 | -3.703 | -16.428 | 0.2917 | -47.166 | 14.311 |
| Holiday β_6_ | -27.649 | 0.0089 | -48.217 | -7.081 | -93.335 | 0.0007 | -146.134 | -40.535 |

Coef., coefficient

CI, confidence interval.

**eTable 5. Changes in cancer care utilizations in West China Hospital Cancer Center, stratified by service type and rurality.**

|  | **Coef.** | **P value** | **95%CI(Lower)** | **95%CI(Upper)** | **Coef.** | **P value** | **95%CI(Lower)** | **95%CI(Upper)** |
| --- | --- | --- | --- | --- | --- | --- | --- | --- |
|  | **Urban** | | | | **Rural** | | | |
| **Inpatient unit (non-surgery)** | | | | | | | | |
| Constant β_0_ | 206.646 | 0.2588 | -154.257 | 567.549 | 91.181 | 0.5739 | -229.399 | 411.762 |
| Time(weeks)β_1_ | 4.664 | 0.3238 | -4.665 | 13.992 | 4.451 | 0.2892 | -3.835 | 12.738 |
| Seasonality terms β_ck_ and β_sk_ |  |  |  |  |  |  |  |  |
| Cos1 | 66.150 | 0.6135 | -192.810 | 325.109 | 165.815 | 0.1558 | -64.212 | 395.842 |
| Sin1 | -51.454 | 0.6555 | -279.545 | 176.637 | 68.903 | 0.5015 | -133.704 | 271.510 |
| Cos2 | 29.051 | 0.4173 | -41.694 | 99.796 | 49.044 | 0.1247 | -13.797 | 111.884 |
| Sin2 | 3.570 | 0.9194 | -66.248 | 73.387 | 2.938 | 0.9253 | -59.080 | 64.956 |
| Pandemic outbreak β_2_ | -309.362 | 0.0000 | -389.027 | -229.696 | -275.060 | 0.0000 | -345.825 | -204.296 |
| Pandemic remission β_3_ | 57.270 | 0.1302 | -17.179 | 131.718 | 51.284 | 0.1271 | -14.847 | 117.414 |
| Outbreak-time interaction β_4_ | 20.081 | 0.0128 | 4.354 | 35.808 | 23.919 | 0.0010 | 9.949 | 37.888 |
| Remission-time interaction β_5_ | -25.580 | 0.0019 | -41.482 | -9.678 | -27.453 | 0.0002 | -41.578 | -13.328 |
| Holiday β_6_ | -136.706 | 0.0000 | -164.020 | -109.391 | -121.439 | 0.0000 | -145.701 | -97.176 |
| **Inpatient unit (surgery)** | | | | | | | | |
| Constant β_0_ | 4.614 | 0.9857 | -505.304 | 514.532 | 82.071 | 0.6878 | -321.838 | 485.980 |
| Time(weeks)β_1_ | 5.119 | 0.4429 | -8.061 | 18.299 | 2.886 | 0.5847 | -7.554 | 13.326 |
| Seasonality terms β_ck_ and β_sk_ |  |  |  |  |  |  |  |  |
| Cos1 | 113.875 | 0.5384 | -252.011 | 479.761 | 146.328 | 0.3190 | -143.490 | 436.146 |
| Sin1 | 174.040 | 0.2866 | -148.226 | 496.306 | 117.563 | 0.3632 | -137.707 | 372.833 |
| Cos2 | 65.527 | 0.1964 | -34.427 | 165.481 | 55.587 | 0.1668 | -23.588 | 134.762 |
| Sin2 | -12.117 | 0.8080 | -110.764 | 86.530 | -36.990 | 0.3500 | -115.128 | 41.147 |
| Pandemic outbreak β_2_ | -383.291 | 0.0000 | -495.846 | -270.736 | -329.795 | 0.0000 | -418.953 | -240.637 |
| Pandemic remission β_3_ | 70.052 | 0.1895 | -35.133 | 17.236 | 31.296 | 0.4580 | -52.023 | 114.616 |
| Outbreak-time interaction β_4_ | 34.585 | 0.0026 | 12.366 | 56.805 | 34.453 | 0.0002 | 16.853 | 52.054 |
| Remission-time interaction β_5_ | -31.151 | 0.0070 | -53.618 | -8.864 | -30.958 | 0.0008 | -48.754 | -13.161 |
| Holiday β_6_ | -160.224 | 0.0000 | -198.816 | -121.633 | -136.125 | 0.0000 | -166.694 | -105.555 |
| **Outpatient physician visit unit (diagnosed)** | | | | | | | | |
| Constant β_0_ | 2343.230 | 0.0122 | 521.577 | 4164.882 | -1167.133 | 0.1322 | -2692.305 | 358.040 |
| Time(weeks)β_1_ | 0.627 | 0.9790 | -46.459 | 47.713 | 75.867 | 0.0002 | 36.444 | 115.290 |
| Seasonality terms β_ck_ and β_sk_ |  |  |  |  |  |  |  |  |
| Cos1 | -528.233 | 0.4247 | -1835.330 | 778.864 | 2127.403 | 0.00002 | 1033.040 | 3221.765 |
| Sin1 | -189.233 | 0.7450 | -1340.642 | 962.002 | -79.965 | 0.8696 | -1043.882 | 883.953 |
| Cos2 | -70.769 | 0.6951 | -427.859 | 286.320 | 496.291 | 0.0014 | 197.324 | 795.259 |
| Sin2 | 94.645 | 0.5954 | -257.772 | 447.062 | -220.283 | 0.1417 | -515.336 | 74.770 |
| Pandemic outbreak β_2_ | -2152.509 | 0.0000 | -2554.635 | -1750.383 | -1535.026 | 0.0000 | -1871.691 | -1198.362 |
| Pandemic remission β_3_ | 53.627 | 0.7777 | -322.158 | 429.413 | -15.089 | 0.9244 | -329.709 | 299.530 |
| Outbreak-time interaction β_4_ | 224.159 | 0.0000 | 144.778 | 303.540 | 85.666 | 0.0120 | 19.205 | 152.217 |
| Remission-time interaction β_5_ | -230.458 | 0.0000 | -310.726 | -150.191 | -155.633 | 0.0000 | -222.833 | -88.432 |
| Holiday β_6_ | -675.746 | 0.0000 | -813.619 | -537.873 | -531.099 | 0.00000 | -646.529 | -415.669 |
| **Outpatient physician visit unit (suspected)** | | | | | | | | |
| Constant β_0_ | 407.890 | 0.0471 | 5.383 | 810.396 | -160.810 | 0.3358 | -490.611 | 168.991 |
| Time(weeks)β_1_ | -1.849 | 0.7252 | -12.253 | 8.555 | 6.539 | 0.1313 | -1.986 | 15.063 |
| Seasonality terms β_ck_ and β_sk_ |  |  |  |  |  |  |  |  |
| Cos1 | -106.051 | 0.4681 | -394.863 | 182.761 | 372.059 | 0.0024 | 135.416 | 608.702 |
| Sin1 | -66.791 | 0.6037 | -321.174 | 187.592 | 101.478 | 0.3365 | -106.956 | 309.912 |
| Cos2 | 33.006 | 0.4087 | -45.894 | 111.906 | 63.000 | 0.0560 | -1.648 | 127.648 |
| Sin2 | -31.783 | 0.4201 | -109.649 | 46.083 | -127.902 | 0.0001 | -191.704 | -64.101 |
| Pandemic outbreak β_2_ | -282.789 | 0.0000 | -371.638 | -193.941 | -190.812 | 0.0000 | -263.612 | -118.013 |
| Pandemic remission β_3_ | 13.034 | 0.7560 | -69.987 | 96.073 | -14.387 | 0.6758 | -82.419 | 53.646 |
| Outbreak-time interaction β_4_ | 18.261 | 0.0415 | 0.721 | 35.800 | 9.947 | 0.1728 | -4.424 | 24.319 |
| Remission-time interaction β_5_ | -15.105 | 0.0942 | -32.839 | 35.800 | -6.428 | 0.3824 | -20.959 | 8.103 |
| Holiday β_6_ | -89.226 | 0.0000 | -119.689 | -58.763 | -58.758 | 0.0000 | -83.719 | -33.798 |
| **Outpatient chemotherapy unit** | | | | | | | | |
| Constant β_0_ | -159.064 | 0.2871 | -135.777 | 453.906 | 44.145 | 0.6999 | -182.370 | 270.659 |
| Time(weeks)β_1_ | 1.421 | 0.7123 | -6.200 | 9.042 | 2.497 | 0.3995 | -3.358 | 8.352 |
| Seasonality terms β_ck_ and β_sk_ |  |  |  |  |  |  |  |  |
| Cos1 | -28.063 | 0.7930 | -239.622 | 183.495 | 87.606 | 0.2876 | -74.926 | 250.137 |
| Sin1 | -3.483 | 0.9705 | -189.821 | 182.855 | 20.977 | 0.7719 | -122.179 | 164.134 |
| Cos2 | -4.440 | 0.8792 | -62.235 | 53.356 | 18.964 | 0.3989 | -25.438 | 63.366 |
| Sin2 | 33.546 | 0.2461 | -23.492 | 90.583 | 1.961 | 0.9294 | -41.859 | 45.781 |
| Pandemic outbreak β_2_ | -147.651 | 0.0000 | -212.734 | -82.568 | -74.767 | 0.0038 | -124.767 | -24.766 |
| Pandemic remission β_3_ | 41.462 | 0.1793 | -19.359 | 102.283 | 14.395 | 0.5426 | -32.332 | 61.121 |
| Outbreak-time interaction β_4_ | 8.125 | 0.2126 | -4.723 | 20.973 | 1.940 | 0.6975 | -7.931 | 11.810 |
| Remission-time interaction β_5_ | -10.487 | 0.1124 | -23.478 | 2.504 | -4.072 | 0.4203 | -14.053 | 5.908 |
| Holiday β_6_ | -68.117 | 0.0000 | -90.431 | -45.802 | -56.273 | 0.0000 | -73.416 | -39.129 |
| **Outpatient radiotherapy unit** | | | | | | | | |
| Constant β_0_ | 120.007 | 0.0564 | -3.338 | 243.352 | -41.280 | 0.4768 | -155.924 | 73.364 |
| Time(weeks)β_1_ | -0.655 | 0.6843 | -3.844 | 2.533 | 1.492 | 0.3202 | -1.471 | 4.456 |
| Seasonality terms β_ck_ and β_sk_ |  |  |  |  |  |  |  |  |
| Cos1 | -42.607 | 0.3419 | -131.111 | 45.897 | 81.120 | 0.0532 | -1.141 | 163.381 |
| Sin1 | -26.419 | 0.5030 | -104.373 | 51.535 | 85.262 | 0.0215 | 12.808 | 157.717 |
| Cos2 | -10.970 | 0.3703 | -35.148 | 13.209 | 27.516 | 0.0169 | 5.043 | 49.988 |
| Sin2 | 14.567 | 0.2288 | -9.295 | 38.428 | -10.763 | 0.3381 | -32.941 | 11.415 |
| Pandemic outbreak β_2_ | -21.981 | 0.1124 | -49.208 | 5.246 | -42.084 | 0.0013 | -67.391 | -16.778 |
| Pandemic remission β_3_ | 13.929 | 0.2801 | -11.515 | 39.373 | 2.746 | 0.8183 | -20.903 | 26.395 |
| Outbreak-time interaction β_4_ | 2.587 | 0.3420 | -2.787 | 7.962 | 4.203 | 0.0982 | -0.792 | 9.199 |
| Remission-time interaction β_5_ | -2.665 | 0.3331 | -8.099 | 2.770 | -2.819 | 0.2710 | -7.870 | 2.233 |
| Holiday β_6_ | -48.043 | 0.0000 | -57.378 | -38.708 | -41.637 | 0.0000 | -50.314 | -32.961 |
| **Pharmacy and Laboratory unit** | | | | | | | | |
| Constant β_0_ | 1181.081 | 0.0038 | 389.6445 | 1972.517 | 437.879 | 0.0776 | -49.227 | 924.985 |
| Time(weeks)β_1_ | -18.784 | 0.0715 | -39.242 | 1.673 | 3.691 | 0.5622 | -8.900 | 16.282 |
| Seasonality terms β_ck_ and β_sk_ |  |  |  |  |  |  |  |  |
| Cos1 | -485.738 | 0.0928 | -1053.620 | 82.144 | 36.968 | 0.8343 | -312.547 | 386.483 |
| Sin1 | 336.831 | 0.1846 | -163.355 | 837.016 | -67.310 | 0.6655 | -375.160 | 240.540 |
| Cos2 | -30.160 | 0.7006 | -185.298 | 124.979 | -19.164 | 0.6914 | -114.648 | 76.319 |
| Sin2 | 78.247 | 0.3121 | -74.679 | 231.532 | 17.461 | 0.7140 | -76.771 | 111.694 |
| Pandemic outbreak β_2_ | -200.196 | 0.0251 | -374.896 | -25.496 | -260.444 | 0.0000 | -367.966 | -152.921 |
| Pandemic remission β_3_ | -56.219 | 0.4962 | -219.478 | 107.040 | 29.207 | 0.5656 | -71.275 | 129.688 |
| Outbreak-time interaction β_4_ | 25.870 | 0.1399 | -8.617 | 60.358 | 10.439 | 0.3316 | -10.787 | 31.665 |
| Remission-time interaction β_5_ | 2.986 | 0.8655 | -31.885 | 37.857 | -18.804 | 0.0853 | -40.267 | 2.658 |
| Holiday β_6_ | -258.593 | 0.0000 | -318.492 | -198.695 | -143.486 | 0.0000 | -180.352 | -106.620 |

Coef., coefficient

CI, confidence interval.

**eTable 6. Changes in cancer care utilization in West China Hospital Cancer Center, stratified by service type and residence distance.**

|  | **Coef.** | **P value** | **L 95%CI** | **U 95%CI** | **Coef.** | **P value** | **L 95%CI** | **U 95%CI** | **Coef.** | **Pvalue** | **L 95%CI** | **U 95%CI** |
| --- | --- | --- | --- | --- | --- | --- | --- | --- | --- | --- | --- | --- |
|  | **Chengdu** | | | | **Outside Chengdu** | | | | **Outside Sichuan** | | | |
| **Inpatient unit (non-surgery)** | | | | | | | | | | | | |
| Constant β_0_ | -58.480 | 0.7044 | -363.354 | 246.395 | 290.197 | 0.0862 | -41.996 | 622.390 | 66.113 | 0.1450 | -23.180 | 155.406 |
| Time(weeks)β_1_ | 9.036 | 0.0250 | 1.156 | 16.917 | 0.299 | 0.9451 | -8.288 | 8.885 | -0.220 | 0.8503 | -2.528 | 2.088 |
| Seasonality terms β_ck_ and β_sk_ |  |  |  |  |  |  |  |  |  |  |  |  |
| Cos1 | 179.376 | 0.1070 | -39.382 | 398.134 | 52.416 | 0.6637 | -185.943 | 290.774 | 0.171 | 0.9958 | -63.898 | 64.241 |
| Sin1 | -47.852 | 0.6234 | -240.533 | 144.830 | 50.466 | 0.6346 | -159.489 | 260.421 | 14.836 | 0.6032 | -41.590 | 71.262 |
| Cos2 | 70.620 | 0.0210 | 10.858 | 130.382 | 6.041 | 0.8544 | -59.076 | 71.159 | 1.432 | 0.8714 | -16.071 | 18.935 |
| Sin2 | -0.407 | 0.9891 | -59.387 | 58.573 | 1.183 | 0.9709 | -63.082 | 65.448 | 5.372 | 0.5119 | -11.540 | 23.003 |
| Pandemic outbreak β_2_ | -227.513 | 0.0000 | -294.810 | -160.216 | -300.618 | 0.0000 | -373.947 | -227.290 | -56.291 | 0.0000 | -75.998 | -36.583 |
| Pandemic remission β_3_ | 29.159 | 0.3600 | -33.731 | 92.048 | 62.212 | 0.0747 | -6.315 | 130.739 | 17.183 | 0.0671 | -1.235 | 35.600 |
| Outbreak-time interaction β_4_ | 18.324 | 0.0073 | 5.039 | 31.609 | 22.955 | 0.0022 | 8.480 | 37.431 | 2.720 | 0.1685 | -1.170 | 6.611 |
| Remission-time interaction β_5_ | -28.596 | 0.0001 | -42.029 | -15.163 | -22.102 | 0.0034 | -36.739 | -7.465 | -2.335 | 0.2418 | -6.269 | 1.599 |
| Holiday β_6_ | -104.776 | 0.0000 | -127.850 | -81.703 | -128.155 | 0.0000 | -153.297 | -103.013 | -25.213 | 0.0000 | -31.970 | -18.456 |
| **Inpatient unit (surgery)** | | | | | | | | |  |  |  |  |
| Constant β_0_ | 195.411 | 0.4030 | -266.140 | 656.962 | -133.504 | 0.4761 | -503.727 | 236.718 | 22.779 | 0.6560 | -85.241 | 134.799 |
| Time(weeks)β_1_ | 2.693 | 0.6553 | -9.237 | 14.624 | 4.995 | 0.3030 | -4.575 | 14.564 | 0.317 | 0.8254 | -2.527 | 3.161 |
| Seasonality terms β_ck_ and β_sk_ |  |  |  |  |  |  |  |  |  |  |  |  |
| Cos1 | -15.911 | 0.9243 | -347.090 | 315.267 | 253.182 | 0.0615 | -21.465 | 518.829 | 22.931 | 0.5658 | -56.012 | 101.874 |
| Sin1 | -19.313 | 0.8958 | -311.010 | 272.384 | 270.504 | 0.0239 | 36.522 | 504.486 | 40.410 | 0.2517 | -29.123 | 109.942 |
| Cos2 | 3.289 | 0.9427 | -87.185 | 93.763 | 102.706 | 0.0060 | 30.135 | 175.278 | 15.118 | 0.1674 | -6.448 | 36.684 |
| Sin2 | 40.061 | 0.3756 | -49.227 | 129.349 | -77.176 | 0.0350 | -148.797 | -5.555 | -11.992 | 0.2664 | -33.276 | 9.292 |
| Pandemic outbreak β_2_ | -314.623 | 0.0000 | -416.505 | -212.742 | -322.783 | 0.0000 | -404.506 | -241.061 | -75.680 | 0.0000 | -99.966 | -51.395 |
| Pandemic remission β_3_ | 72.897 | 0.1320 | -22.313 | 168.107 | 9.122 | 0.8132 | -67.249 | 85.493 | 19.329 | 0.0942 | -3.366 | 42.024 |
| Outbreak-time interaction β_4_ | 31.085 | 0.0028 | 10.973 | 51.198 | 31.945 | 0.0002 | 15.813 | 48.078 | 6.008 | 0.0146 | 1.213 | 10.802 |
| Remission-time interaction β_5_ | -32.937 | 0.0018 | -53.273 | -12.601 | -24.594 | 0.0035 | -40.906 | -8.281 | -4.578 | 0.0639 | -9.425 | 0.270 |
| Holiday β_6_ | -140.519 | 0.0000 | -175.451 | -105.587 | -122.535 | 0.0000 | -150.554 | -94.515 | -33.295 | 0.0000 | -41.622 | -24.968 |
| **Outpatient physician visit unit (diagnosed)** | | | | | | | | |  |  |  |  |
| Constant β_0_ | 1266.858 | 0.0860 | -182.584 | 2716.300 | 9.455 | 0.9906 | -1574.654 | 1593.565 | -100.274 | 0.5432 | -419.106 | 218.558 |
| Time(weeks)β_1_ | 19.334 | 0.3085 | -18.132 | 56.800 | 45.566 | 0.0295 | 4.629 | 86.503 | 11.595 | 0.0063 | 3.354 | 19.836 |
| Seasonality terms β_ck_ and β_sk_ |  |  |  |  |  |  |  |  |  |  |  |  |
| Cos1 | 19.994 | 0.9697 | -1020.025 | 1060.013 | 1261.078 | 0.0300 | 124.427 | 2397.729 | 318.139 | 0.0069 | 89.366 | 546.912 |
| Sin1 | -343.548 | 0.4587 | -1259.555 | 572.458 | 89.402 | 0.8598 | -911.738 | 1090.541 | -15.124 | 0.8820 | -216.625 | 186.377 |
| Cos2 | 27.917 | 0.8459 | -256.208 | 312.042 | 312.492 | 0.0486 | 1.972 | 623.012 | 85.124 | 0.0081 | 22.626 | 147.623 |
| Sin2 | -41.110 | 0.7718 | -321.497 | 239.277 | -58.595 | 0.7053 | -365.074 | 247.883 | -25.940 | 0.4062 | -87.619 | 35.739 |
| Pandemic outbreak β_2_ | -1772.337 | 0.0000 | -2092.275 | -1452.399 | -1613.849 | 0.0000 | -1963.469 | -1264.229 | -301.351 | 0.0000 | -371.730 | -230.973 |
| Pandemic remission β_3_ | -30.970 | 0.8376 | -329.955 | 268.015 | 56.772 | 0.7311 | -269.956 | 383.500 | 12.736 | 0.7017 | -53.034 | 78.505 |
| Outbreak-time interaction β_4_ | 161.494 | 0.0000 | 98.335 | -119.099 | 129.188 | 0.0003 | 60.174 | 198.202 | 19.142 | 0.0074 | 5.249 | 33.035 |
| Remission-time interaction β_5_ | -182.961 | 0.0000 | -246.822 | -119.099 | -173.195 | 0.0000 | -242.980 | -103.409 | -29.936 | 0.0001 | -43.984 | -15.888 |
| Holiday β_6_ | -515.256 | 0.0000 | -624.951 | -405.561 | -578.599 | 0.0000 | -698.470 | -458.728 | -112.990 | 0.0000 | -137.121 | -88.860 |
| **Outpatient physician visit unit (suspected)** | | | | | | | | | | | | |
| Constant β_0_ | 244.626 | 0.1140 | -59.741 | 548.994 | 0.918 | 0.9956 | -326.963 | 328.798 | 1.528 | 0.9739 | -90.709 | 93.764 |
| Time(weeks)β_1_ | -0.237 | 0.9524 | -8.105 | 7.630 | 3.946 | 0.3579 | -4.529 | 12.421 | 0.980 | 0.4166 | -1.404 | 3.365 |
| Seasonality terms β_ck_ and β_sk_ |  |  |  |  |  |  |  |  |  |  |  |  |
| Cos1 | 9.599 | 0.9307 | -208.795 | 227.994 | 210.100 | 0.0795 | -25.164 | 445.364 | 46.314 | 0.1682 | -19.869 | 112.498 |
| Sin1 | -33.029 | 0.7341 | -225.390 | 159.331 | 60.568 | 0.5634 | -146.647 | 267.784 | 7.149 | 0.8083 | -51.142 | 65.441 |
| Cos2 | 11.772 | 0.6964 | -47.890 | 71.434 | 68.324 | 0.0374 | 4.053 | 132.596 | 15.911 | 0.0839 | -2.169 | 33.990 |
| Sin2 | -46.488 | 0.1205 | -105.369 | 12.393 | -89.865 | 0.0059 | -153.293 | -26.437 | -23.333 | 0.0109 | -41.177 | -5.490 |
| Pandemic outbreak β_2_ | -215.404 | 0.0000 | -54.572 | 70.999 | -212.136 | 0.0000 | -284.510 | -139.762 | -46.062 | 0.0000 | -66.421 | -25.703 |
| Pandemic remission β_3_ | 8.213 | 0.7958 | -54.572 | 70.999 | -12.741 | 0.7095 | -80.375 | 54.894 | 3.183 | 0.7407 | -15.843 | 22.209 |
| Outbreak-time interaction β_4_ | 14.674 | 0.0305 | 1.411 | 27.937 | 12.949 | 0.0752 | -1.339 | 27.236 | 0.585 | 0.7733 | -3.434 | 4.604 |
| Remission-time interaction β_5_ | -11.012 | 0.1065 | -24.422 | 2.399 | -10.370 | 0.1576 | -24.816 | 4.077 | -0.151 | 0.9413 | -4.215 | 3.913 |
| Holiday β_6_ | -67.358 | 0.0000 | -90.393 | -44.322 | -67.624 | 0.0000 | -92.079 | -42.450 | -13.363 | 0.0002 | -20.343 | -6.382 |
| **Outpatient chemotherapy unit** | | | | | | | | | | | | |
| Constant β_0_ | -74.583 | 0.5248 | -306.366 | 157.200 | 139.639 | 0.1995 | -74.840 | 354.119 | 2.736 | 0.8887 | -35.940 | 41.411 |
| Time(weeks)β_1_ | 5.572 | 0.0597 | -0.239 | 11.744 | 1.132 | 0.6864 | -4.412 | 6.676 | 1.568 | 0.224 | 0.568 | 2.567 |
| Seasonality terms β_ck_ and β_sk_ |  |  |  |  |  |  |  |  |  |  |  |  |
| Cos1 | 140.131 | 0.0977 | -26.182 | 306.443 | 2.266 | 0.9768 | -151.630 | 156.162 | 11.898 | 0.3935 | -15.762 | 39.741 |
| Sin1 | 43.447 | 0.5577 | -103.039 | 189.934 | -12.829 | 0.8515 | -148.375 | 122.718 | -44.137 | 0.0005 | -68.580 | -19.694 |
| Cos2 | 51.408 | 0.0270 | 5.973 | 96.842 | -0.900 | 0.6829 | -32.921 | 50.060 | 1.720 | 0.6537 | -5.861 | 9.031 |
| Sin2 | -22.771 | 0.3162 | -67.610 | 22.068 | 8.569 | 0.6829 | -32.921 | 50.060 | 7.751 | 0.0425 | 0.269 | 15.233 |
| Pandemic outbreak β_2_ | -138.384 | 0.0000 | -189.547 | -87.220 | -106.213 | 0.0000 | -153.555 | -58.870 | -30.081 | 0.0000 | -39.338 | -22.264 |
| Pandemic remission β_3_ | -8.065 | 0.7387 | -55.878 | 39.748 | 10.553 | 0.6372 | -33.690 | 54.795 | 2.893 | 0.4736 | -5.085 | 10.871 |
| Outbreak-time interaction β_4_ | 6.095 | 0.2341 | -4.005 | 16.195 | 3.968 | 0.4017 | -5.377 | 13.314 | 0.540 | 0.5265 | -1.145 | 2.225 |
| Remission-time interaction β_5_ | -8.542 | 0.1002 | -18.754 | 1.671 | -6.291 | 0.1897 | -15.741 | 3.159 | -3.988 | 0.0000 | -5.962 | -2.284 |
| Holiday β_6_ | -50.011 | 0.0000 | -67.554 | -32.469 | -53.299 | 0.0000 | -69.531 | -37.066 | -8.389 | 0.0000 | -11.316 | -5.462 |
| **Outpatient radiotherapy unit** | | | | | | | | | | | | |
| Constant β_0_ | 97.254 | 0.0445 | 2.436 | 193.072 | 18.289 | 0.7570 | -98.640 | 135.219 | -37.316 | 0.1150 | -83.869 | 9.237 |
| Time(weeks)β_1_ | -1.440 | 0.2490 | -3.904 | 1.024 | 1.109 | 0.4684 | -1.913 | 4.131 | 1.168 | 0.0570 | -0.035 | 2.371 |
| Seasonality terms β_ck_ and β_sk_ |  |  |  |  |  |  |  |  |  |  |  |  |
| Cos1 | -33.196 | 0.3380 | -101.589 | 35.198 | 29.992 | 0.4800 | -53.909 | 113.893 | 41.7717 | 0.0149 | 8.313 | 75.120 |
| Sin1 | 7.711 | 0.8001 | -52.530 | 67.952 | 32.055 | 0.3916 | -41.843 | 105.954 | 19.077 | 0.2013 | -10.344 | 48.499 |
| Cos2 | -11.639 | 0.2195 | -30.323 | 7.046 | 14.179 | 0.2227 | -8.742 | 37.099 | 14.006 | 0.0030 | 4.881 | 23.131 |
| Sin2 | -3.304 | 0.7231 | -21.743 | 15.136 | 1.223 | 0.9148 | -21.397 | 23.844 | 5.884 | 0.1979 | 3.122 | 14.890 |
| Pandemic outbreak β_2_ | -16.313 | 0.1272 | -37.354 | 4.727 | -37.808 | 0.0045 | -63.618 | -11.997 | -9.944 | 0.0577 | -20.220 | 0.332 |
| Pandemic remission β_3_ | 5.242 | 0.5981 | -14.421 | 24.904 | 6.080 | 0.6182 | -18.040 | 30.201 | 5.353 | 0.2715 | -4.250 | 14.956 |
| Outbreak-time interaction β_4_ | 3.204 | 0.1291 | -0.949 | 7.358 | 2.822 | 0.2745 | -2.273 | 7.917 | 0.764 | 0.4568 | -1.264 | 2.793 |
| Remission-time interaction β_5_ | -0.686 | 0.7466 | -4.886 | 3.514 | -3.183 | 0.2232 | -8.335 | 1.969 | -1.614 | 0.1217 | -3.665 | 0.437 |
| Holiday β_6_ | -34.167 | 0.0000 | -41.381 | -26.953 | -44.044 | 0.0000 | -52.893 | -35.194 | -11.470 | 0.0000 | -14.993 | -7.946 |
| **Pharmacy and Laboratory unit** | | | | | | | | | | | | |
| Constant β_0_ | 293.404 | 0.4688 | -506.894 | 1093.703 | 245.120 | 0.3903 | -318.391 | 808.632 | -24.846 | 0.6173 | -123.158 | 73.466 |
| Time(weeks)β_1_ | 15.338 | 0.1445 | -5.348 | 36.024 | 9.767 | 0.1865 | -4.799 | 24.332 | 3.256 | 0.0125 | 0.715 | 5.797 |
| Seasonality terms β_ck_ and β_sk_ |  |  |  |  |  |  |  |  |  |  |  |  |
| Cos1 | 203.351 | 0.4841 | -370.890 | 777.592 | 162.923 | 0.4261 | -241.415 | 567.262 | 42.074 | 0.2396 | -28.468 | 112.616 |
| Sin1 | -137.180 | 0.5918 | -642.952 | 368.592 | -63.456 | 0.7245 | -419.594 | 292.682 | -59.989 | 0.0583 | -122.123 | 2.144 |
| Cos2 | 44.780 | 0.5725 | -112.095 | 201.655 | 43.496 | 0.4366 | -66.965 | 153.956 | 12.134 | 0.2146 | -7.137 | 31.046 |
| Sin2 | 0.924 | 0.9906 | -153.892 | 155.740 | 25.902 | 0.6385 | -83.111 | 134.916 | 5.331 | 0.5795 | -13.688 | 24.349 |
| Pandemic outbreak β_2_ | -769.008 | 0.0000 | -945.659 | -592.358 | -476.305 | 0.0000 | -600.693 | -351.917 | -25.969 | 0.0195 | -47.670 | -4.268 |
| Pandemic remission β_3_ | -137.018 | 0.1028 | -302.097 | 28.061 | 67.541 | 0.2518 | -48.701 | 183.784 | 12.727 | 0.2161 | -7.554 | 33.007 |
| Outbreak-time interaction β_4_ | 61.011 | 0.0008 | 26.139 | 95.883 | 25.856 | 0.0392 | 1.301 | 50.412 | -2.957 | 0.1739 | -7.241 | 1.327 |
| Remission-time interaction β_5_ | -81.750 | 0.0000 | -117.012 | -46.489 | -41.633 | 0.0012 | -66.462 | -16.804 | -2.609 | 0.2350 | -6.941 | 1.723 |
| Holiday β_6_ | -216.656 | 0.0000 | -277.224 | -156.089 | -159.450 | 0.0000 | -202.098 | -116.802 | -17.863 | 0.0000 | -25.303 | -10.422 |

Coef., coefficient

CI, confidence interval.

L 95%CI, 95%CI(Lower)

U 95%CI, 95%CI(Upper)

**eTable 7. Changes in cancer care utilization in West China Hospital Cancer Center, stratified by service type and payment method.**

|  | **Coef.** | **P value** | **95%CI(Lower)** | **95%CI(Upper)** | **Coef.** | **P value** | **95%CI(Lower)** | **95%CI(Upper)** |  |
| --- | --- | --- | --- | --- | --- | --- | --- | --- | --- |
|  | **Insured** | | | | **Uninsured** | | | | |
| **Inpatient unit (non-surgery)** | | | | | | | | | |
| Constant β_0_ | 221.608 | 0.3927 | -290.401 | 733.617 | 76.220 | 0.3900 | -98.878 | 251.317 |  |
| Time(weeks)β_1_ | 6.120 | 0.3613 | -7.115 | 19.354 | 2.995 | 0.1923 | -1.531 | 7.521 |  |
| Seasonality terms β_ck_ and β_sk_ |  |  |  |  |  |  |  |  |  |
| Cos1 | 152.901 | 0.4110 | -214.482 | 520.285 | 79.063 | 0.2148 | -46.576 | 204.701 |  |
| Sin1 | 57.770 | 0.7240 | -265.821 | 381.361 | -40.320 | 0.4716 | -150.982 | 70.341 |  |
| Cos2 | 53.916 | 0.2892 | -46.449 | 154.281 | 24.178 | 0.1654 | -10.145 | 58.501 |  |
| Sin2 | 3.645 | 0.9420 | -95.406 | 102.695 | 2.863 | 0.8672 | -31.010 | 36.736 |  |
| Pandemic outbreak β_2_ | -457.808 | 0.0000 | -570.827 | -344.789 | -126.614 | 0.0000 | -165.264 | -87.963 |  |
| Pandemic remission β_3_ | 76.220 | 0.1554 | -29.399 | 181.838 | 32.334 | 0.0788 | -3.786 | 68.453 |  |
| Outbreak-time interaction β_4_ | 36.953 | 0.0014 | 14.641 | 59.264 | 7.047 | 0.0699 | -0.583 | 14.677 |  |
| Remission-time interaction β_5_ | -40.969 | 0.0005 | -63.528 | -18.410 | -12.064 | 0.0025 | -19.779 | -4.349 |  |
| Holiday β_6_ | -201.476 | 0.0000 | -240.226 | -162.726 | -56.668 | 0.0000 | -69.920 | -43.416 |  |
| **Inpatient unit (surgery)** | | | | | | | | | |
| Constant β_0_ | 64.148 | 0.8513 | -612.797 | 741.094 | 22.541 | 0.8436 | -203.537 | 248.619 |  |
| Time(weeks)β_1_ | 4.705 | 0.5950 | -12.793 | 22.203 | 3.300 | 0.2653 | -2.544 | 9.144 |  |
| Seasonality terms β_ck_ and β_sk_ |  |  |  |  |  |  |  |  |  |
| Cos1 | 163.299 | 0.5062 | -322.332 | 649.130 | 96.800 | 0.2393 | -65.418 | 259.019 |  |
| Sin1 | 272.419 | 0.2095 | -155.409 | 700.246 | 19.181 | 0.7906 | -123.698 | 162.061 |  |
| Cos2 | 73.370 | 0.2754 | -59.326 | 206.066 | 47.743 | 0.0350 | 3.427 | 92.059 |  |
| Sin2 | -33.105 | 0.6172 | -164.062 | 97.852 | -16.001 | 0.4697 | -59.736 | 27.734 |  |
| Pandemic outbreak β_2_ | -543.949 | 0.0000 | -693.376 | -394.522 | -169.138 | 0.0000 | -219.041 | -119.234 |  |
| Pandemic remission β_3_ | 72.830 | 0.3034 | -66.812 | 212.471 | 28.519 | 0.2280 | -18.117 | 75.154 |  |
| Outbreak-time interaction β_4_ | 55.930 | 0.0003 | 26.431 | 85.428 | 13.109 | 0.0096 | 3.258 | 22.960 |  |
| Remission-time interaction β_5_ | -46.657 | 0.0025 | -76.484 | -16.831 | -15.451 | 0.0027 | -25.412 | -5.490 |  |
| Holiday β_6_ | -225.759 | 0.0000 | -276.992 | -174.526 | -70.590 | 0.0000 | -87.700 | -53.480 |  |
| **Outpatient physician visit unit (diagnosed)** | | | | | | | | | |
| Constant β_0_ | -132.008 | 0.7611 | -990.990 | 726.974 | 2229.429 | 0.0684 | -171.807 | 4630.664 |  |
| Time(weeks)β_1_ | 32.923 | 0.0040 | 10.719 | 55.126 | 23.077 | 0.4626 | -38.991 | 85.145 |  |
| Seasonality terms β_ck_ and β_sk_ |  |  |  |  |  |  |  |  |  |
| Cos1 | 717.815 | 0.0229 | 101.467 | 1334.163 | 461.014 | 0.5968 | -1261.952 | 2183.980 |  |
| Sin1 | -321.978 | 0.2422 | -864.860 | 220.905 | -251.715 | 0.7429 | -1769.305 | 1265.876 |  |
| Cos2 | 162.003 | 0.0592 | -6.377 | 330.383 | 124.389 | 0.6013 | -346.306 | 595.083 |  |
| Sin2 | -30.044 | 0.7206 | -196.218 | 136.130 | 20.104 | 0.9318 | -444.423 | 484.630 |  |
| Pandemic outbreak β2 | -771.552 | 0.0000 | -961.165 | -581.939 | -2593.693 | 0.0000 | -2123.738 | -2063.648 |  |
| Pandemic remission β_3_ | -35.707 | 0.6902 | -212.902 | 141.488 | 192.902 | 0.4417 | -302.432 | 688.236 |  |
| Outbreak-time interaction β_4_ | 77.451 | 0.0001 | 40.020 | 114.883 | 247.516 | 0.0000 | 142.880 | 352.152 |  |
| Remission-time interaction β_5_ | -119.392 | 0.0000 | -157.240 | -81.544 | -277.688 | 0.0000 | -383.490 | -171.887 |  |
| Holiday β_6_ | -367.255 | 0.0000 | -432.266 | -302.243 | -1139.748 | 0.0000 | -1321.482 | -958.014 |  |
| **Outpatient physician visit unit (suspected)** | | | | | | | | | |
| Constant β_0_ | -8.175 | 0.7761 | -65.049 | 48.698 | -161.923 | 0.4077 | -548.180 | 224.334 |  |
| Time(weeks)β_1_ | 1.300 | 0.0823 | -0.170 | 2.771 | 9.129 | 0.0700 | -0.765 | 19.203 |  |
| Seasonality terms β_ck_ and β_sk_ |  |  |  |  |  |  |  |  |  |
| Cos1 | 36.025 | 0.0830 | -4.783 | 76.833 | 352.421 | 0.0132 | 75.268 | 629.574 |  |
| Sin1 | 8.268 | 0.6492 | -27.676 | 44.212 | 245.322 | 0.0489 | 1.230 | 489.413 |  |
| Cos2 | 10.653 | 0.0609 | -0.496 | 21.801 | 128.444 | 0.0011 | 52.729 | 204.159 |  |
| Sin2 | -8.342 | 0.1357 | -19.344 | 2.661 | -97.452 | 0.0111 | -172.166 | -22.738 |  |
| Pandemic outbreak β_2_ | -33.190 | 0.0000 | -45.744 | 13.020 | -420.713 | 0.0000 | -505.976 | -335.450 |  |
| Pandemic remission β_3_ | 1.288 | 0.8280 | -10.444 | 13.020 | -0.359 | 0.9929 | -80.041 | 79.323 |  |
| Outbreak-time interaction β_4_ | 1.309 | 0.2972 | -1.169 | 3.788 | 28.111 | 0.0013 | 11.280 | 44.942 |  |
| Remission-time interaction β_5_ | -1.730 | 0.1740 | -4.236 | 0.776 | -22.399 | 0.0104 | -39.418 | -5.380 |  |
| Holiday β_6_ | -16.800 | 0.0000 | -21.104 | -12.495 | -109.912 | 0.0000 | -139.146 | -80.678 |  |
| **Outpatient chemotherapy unit** | | | | | | | | | |
| Constant β_0_ | 104.367 | 0.3692 | -125.124 | 333.858 | 238.342 | 0.0088 | 61.467 | 415.217 |  |
| Time(weeks)β_1_ | 2.613 | 0.3844 | -3.319 | 8.545 | -2.549 | 0.2714 | -7.121 | 2.023 |  |
| Seasonality terms β_ck_ and β_sk_ |  |  |  |  |  |  |  |  |  |
| Cos1 | 54.804 | 0.5017 | -109.863 | 219.471 | -56.632 | 0.3782 | -183.545 | 70.282 |  |
| Sin1 | 14.195 | 0.8465 | -130.843 | 159.234 | 24.388 | 0.6661 | -87.396 | 136.173 |  |
| Cos2 | 13.910 | 0.5411 | -31.075 | 58.895 | -14.023 | 0.4243 | -48.694 | 20.648 |  |
| Sin2 | -23.497 | 0.2963 | -67.893 | 20.899 | 19.339 | 0.2649 | -14.878 | 53.557 |  |
| Pandemic outbreak β_2_ | -197.215 | 0.0000 | -247.873 | -146.558 | -47.796 | 0.0169 | -86.839 | -8.753 |  |
| Pandemic remission β_3_ | 9.773 | 0.6831 | -37.568 | 57.113 | 9.845 | 0.5937 | -26.642 | 46.331 |  |
| Outbreak-time interaction β_4_ | 13.469 | 0.0088 | 3.469 | 23.469 | 0.715 | 0.8544 | -6.993 | 8.422 |  |
| Remission-time interaction β_5_ | -13.750 | 0.0082 | -23.861 | -3.638 | 1.736 | 0.6596 | -6.057 | 9.529 |  |
| Holiday β_6_ | -78.708 | 0.0000 | -96.077 | -61.340 | -41.641 | 0.0000 | -55.027 | -28.254 |  |
| **Outpatient radiotherapy unit** | | | | | | | | | |
| Constant β_0_ | 66.637 | 0.0259 | 8.157 | 125.116 | 12.091 | 0.8849 | -153.145 | 177.327 |  |
| Time(weeks)β_1_ | -0.905 | 0.2377 | -2.417 | 0.606 | 1.742 | 0.4204 | -2.529 | 6.013 |  |
| Seasonality terms β_ck_ and β_sk_ |  |  |  |  |  |  |  |  |  |
| Cos1 | -29.336 | 0.1686 | -71.297 | 12.625 | 67.849 | 0.2590 | -50.714 | 186.412 |  |
| Sin1 | -12.794 | 0.4939 | -49.753 | 24.165 | 71.638 | 0.1766 | -32.791 | 176.067 |  |
| Cos2 | -12.805 | 0.0289 | -24.268 | -1.342 | 29.351 | 0.0752 | -3.039 | 61.741 |  |
| Sin2 | -3.505 | 0.5402 | -14.818 | 7.807 | 7.309 | 0.6511 | -24.656 | 39.275 |  |
| Pandemic outbreak β_2_ | -5.373 | 0.4110 | -18.281 | 7.536 | -58.692 | 0.0019 | -95.166 | -22.218 |  |
| Pandemic remission β_3_ | 6.717 | 0.2720 | -5.346 | 18.781 | 9.957 | 0.5636 | -24.128 | 44.034 |  |
| Outbreak-time interaction β_4_ | 0.427 | 0.7405 | -2.122 | 2.975 | 6.364 | 0.0826 | -0.836 | 13.564 |  |
| Remission-time interaction β_5_ | 0.467 | 0.7200 | -2.110 | 3.044 | -5.950 | 0.1081 | -13.231 | 1.330 |  |
| Holiday β_6_ | -12.246 | 0.0000 | -16.852 | -8.000 | -77.254 | 0.0000 | -89.760 | -64.749 |  |
| **Pharmacy and Laboratory unit** | | | | | | | | | |
| Constant β_0_ | 296.148 | 0.3142 | -284.544 | 876.841 | 1481.138 | 0.0001 | 779.810 | 2182.466 |  |
| Time(weeks)β_1_ | 0.770 | 0.9191 | -14.240 | 15.780 | -22.862 | 0.0140 | -40.990 | -4.734 |  |
| Seasonality terms β_ck_ and β_sk_ |  |  |  |  |  |  |  |  |  |
| Cos1 | 146.656 | 0.4867 | -270.011 | 563.323 | -691.031 | 0.0076 | -1194.257 | -187.804 |  |
| Sin1 | 308.239 | 0.0988 | -58.754 | 675.232 | 107.579 | 0.6313 | -335.660 | 550.818 |  |
| Cos2 | 52.989 | 0.3580 | -60.838 | 166.817 | -211.053 | 0.0030 | -348.529 | -73.578 |  |
| Sin2 | -29.323 | 0.6058 | -141.659 | 83.013 | -211.053 | 0.0539 | -2.268 | 269.081 |  |
| Pandemic outbreak β_2_ | -411.422 | 0.0000 | -539.603 | -283.242 | -413.488 | 0.0000 | -568.298 | -258.679 |  |
| Pandemic remission β_3_ | -44.437 | 0.4636 | -164.222 | 75.349 | 85.778 | 0.2423 | -58.894 | 230.450 |  |
| Outbreak-time interaction β_4_ | 63.755 | 0.0000 | 38.541 | 89.059 | 42.852 | 0.0064 | 12.291 | 73.413 |  |
| Remission-time interaction β_5_ | -54.090 | 0.0001 | -79.676 | -28.504 | -25.766 | 0.1012 | -56.667 | 5.135 |  |
| Holiday β_6_ | -296.551 | 0.0000 | -340.499 | -252.602 | -212.072 | 0.0000 | -265.151 | -158.993 |  |

Coef., coefficient

CI, confidence interval.

**eTable 8. Changes in cancer care utilization in West China Hospital Cancer Center, stratified by service type and occupation.**

|  | **Coef.** | **P value** | **L 95%CI** | **U 95%CI** | **Coef.** | **P value** | **L95%CI** | **U95%CI 95%CI** | **Coef.** | **P value** | **L 95%CI** | **U95%CI** | **Coef.** | **P value** | **L95%CI** | **U 95%CI** |
| --- | --- | --- | --- | --- | --- | --- | --- | --- | --- | --- | --- | --- | --- | --- | --- | --- |
|  | **Employed by government or companies** | | | | **Retired** | | | | **Peasant** | | | | **Self-employed** | | | |
| **Inpatient unit (non-surgery)** | | | | | | | | | | | | | | | | |
| Constant β_0_ | 327.932 | 0.0860 | -47.275 | 703.140 | 29.403 | 0.7051 | -124.260 | 183.065 | -50.457 | 0.3517 | -157.421 | 56.506 | 78.727 | 0.4391 | -122.312 | 279.767 |
| Time(weeks)β_1_ | -4.966 | 0.3123 | -14.664 | 4.733 | 3.681 | 0.0690 | -0.291 | 7.652 | 3.125 | 0.0271 | 0.360 | 5.890 | 0.837 | 0.7501 | -4.360 | 6.033 |
| Seasonality terms β_ck_ and β_sk_ |  |  |  |  |  |  |  |  |  |  |  |  |  |  |  |  |
| Cos1 | -15.109 | 0.9116 | -264.333 | 254.115 | 41.956 | 0.4522 | -68.303 | 152.214 | 91.896 | 0.0194 | 15.146 | 168.647 | 38.513 | 0.5976 | -105.740 | 182.766 |
| Sin1 | 162.644 | 0.1767 | -74.484 | 399.772 | -61.539 | 0.2117 | -158.653 | 35.576 | 12.285 | 0.7193 | -55.315 | 79.886 | 58.844 | 0.3605 | -68.213 | 185.900 |
| Cos2 | -14.350 | 0.6996 | -87.899 | 59.199 | 23.832 | 0.1197 | -6.289 | 53.953 | 26.437 | 0.0140 | 5.470 | 47.404 | 16.546 | 0.4069 | -22.862 | 55.954 |
| Sin2 | 22.152 | 0.5463 | -50.433 | 94.736 | -3.634 | 0.8089 | -33.360 | 26.093 | -17.788 | 0.0912 | -38.480 | 2.905 | 3.804 | 0.8466 | -35.088 | 42.696 |
| Pandemic outbreak β_2_ | -191.031 | 0.0000 | -273.853 | -108.209 | -127.316 | 0.0000 | -161.235 | -93.397 | -80.933 | 0.0000 | -104.544 | -57.322 | -64.065 | 0.0051 | -108.442 | -19.688 |
| Pandemic remission β_3_ | 19.533 | 0.6174 | -57.845 | 96.951 | 11.487 | 0.4740 | -20.211 | 43.184 | 12.241 | 0.2738 | -9.824 | 34.306 | 16.675 | 0.4270 | -24.796 | 58.146 |
| Outbreak-time interaction β_4_ | 21.871 | 0.0092 | 5.521 | 38.221 | 6.010 | 0.0780 | -0.686 | 12.706 | 2.978 | 0.2080 | -1.683 | 7.639 | 6.791 | 0.1273 | -1.970 | 15.551 |
| Remission-time interaction β_5_ | -12.700 | 0.1307 | -29.231 | 3.832 | -11.024 | 0.0017 | -17.794 | -4.523 | -4.246 | 0.0769 | -8.959 | 0.467 | -5.483 | 0.2224 | -14.341 | 3.375 |
| Holiday β_6_ | -98.491 | 0.0000 | -126.888 | -70.094 | -52.429 | 0.0000 | -64.059 | -40.800 | -30.510 | 0.0000 | -38.606 | -22.4155 | -89.680 | 0.0000 | -104.896 | -74.465 |
| **Inpatient unit (surgery)** | | | | | | | | | | | | | | | | |
| Constant β_0_ | -153.648 | 0.3108 | -452.808 | 145.512 | 130.480 | 0.1106 | -30.312 | 291.272 | -86.736 | 0.3655 | -275.996 | 102.525 | -196.589 | 0.3077 | -183.712 | 576.890 |
| Time(weeks)β_1_ | 7.345 | 0.0624 | -0.388 | 15.078 | -0.854 | 0.6845 | -5.010 | 3.302 | 2.652 | 0.2849 | -2.240 | 7.544 | -1.137 | 0.8190 | -10.967 | 8.693 |
| Seasonality terms β_ck_ and β_sk_ |  |  |  |  |  |  |  |  |  |  |  |  |  |  |  |  |
| Cos1 | 220.287 | 0.0444 | 5.630 | 434.945 | -87.075 | 0.1375 | -202.449 | 28.299 | 155.589 | 0.0251 | 19.788 | 291.390 | -28.600 | 0.8357 | -301.478 | 244.279 |
| Sin1 | 85.944 | 0.3694 | -103.124 | 275.013 | -47.994 | 0.3511 | -149.615 | 53.626 | 82.797 | 0.1728 | -36.815 | 202.410 | 170.853 | 0.1616 | -69.498 | 411.204 |
| Cos2 | 68.611 | 0.0223 | 9.970 | 127.253 | -18.844 | 0.2385 | -50.363 | 12.675 | 34.205 | 0.0696 | -2.794 | 71.404 | 37.041 | 0.3267 | -37.506 | 111.588 |
| Sin2 | -3.087 | 0.9160 | -60.960 | 54.787 | 7.223 | 0.6461 | -23.883 | 38.329 | -32.203 | 0.0841 | -68.816 | 4.410 | -21.040 | 0.5718 | -94.610 | 52.531 |
| Pandemic outbreak β_2_ | -203.925 | 0.0000 | -269.961 | -137.889 | -89.308 | 0.0000 | -124.801 | -53.815 | -98.360 | 0.0000 | -140.137 | -56.583 | -321.494 | 0.0000 | -405.441 | -237.547 |
| Pandemic remission β_3_ | 50.506 | 0.1076 | -11.205 | 112.218 | 13.131 | 0.4342 | -20.038 | 46.299 | 15.494 | 0.4330 | -23.547 | 54.536 | 22.217 | 0.5756 | -56.233 | 100.667 |
| Outbreak-time interaction β_4_ | 14.607 | 0.0284 | 1.571 | 27.644 | 8.242 | 0.0216 | 1.236 | 15.249 | 12.730 | 0.0028 | 4.482 | 20.977 | 33.549 | 0.0001 | 16.887 | 50.031 |
| Remission-time interaction β_5_ | -17.575 | 0.0095 | -30.757 | -4.394 | -8.280 | 0.0224 | -15.365 | -1.195 | -10.271 | 0.0163 | -18.610 | -1.932 | -25.982 | 0.0027 | -42.739 | -9.226 |
| Holiday β_6_ | -88.157 | 0.0000 | -110.798 | -65.516 | -27.250 | 0.0000 | -39.420 | -15.081 | -53.495 | 0.0000 | -67.819 | -39.171 | -127.446 | 0.0000 | -156.229 | -98.664 |
| **Outpatient physician visit unit (diagnosed)** | | | | | | | | | | | | | | | | |
| Constant β_0_ | 352.757 | 0.5099 | -705.233 | 1410.746 | -32.732 | 0.9133 | -627.348 | 561.884 | 78.835 | 0.6567 | -273.070 | 430.740 | 777.250 | 0.2558 | -571.835 | 2126.335 |
| Time(weeks)β_1_ | 20.704 | 0.1363 | -6.643 | 48.051 | 17.979 | 0.0223 | 2.609 | 33.349 | 9.249 | 0.0463 | 0.152 | 18.345 | 28.562 | 0.1073 | -6.309 | 63.433 |
| Seasonality terms β_ck_ and β_sk_ |  |  |  |  |  |  |  |  |  |  |  |  |  |  |  |  |
| Cos1 | 587.429 | 0.1279 | -171.715 | 1326.573 | 383.542 | 0.0776 | -43.115 | 810.198 | 160.794 | 0.2095 | -91.710 | 413.297 | 467.398 | 0.3405 | -500.616 | 1435.412 |
| Sin1 | 78.225 | 0.8170 | -590.422 | 746.871 | -26.869 | 0.8875 | -402.665 | 348.927 | -60.560 | 0.5903 | -282.965 | 161.845 | -260.075 | 0.5465 | -1112.683 | 592.532 |
| Cos2 | 132.355 | 0.2085 | -75.034 | 339.744 | 99.350 | 0.0940 | -17.208 | 215.908 | 50.209 | 0.1519 | -18.772 | 119.191 | 143.606 | 0.2840 | -120.844 | 408.055 |
| Sin2 | -4.074 | 0.9686 | -208.745 | 200.957 | -76.677 | 0.1891 | -191.708 | 38.353 | -18.012 | 0.6009 | -86.090 | 50.065 | -26.875 | 0.8386 | -287.860 | 234.110 |
| Pandemic outbreak β_2_ | -1075.651 | 0.0000 | -1309.191 | -842.111 | -605.164 | 0.0000 | -736.419 | -473.910 | -400.109 | 0.0000 | -477.788 | -322.429 | -1606.610 | 0.0000 | -1904.405 | -1308.816 |
| Pandemic remission β_3_ | -8.008 | 0.9421 | -226.255 | 210.239 | 37.746 | 0.5430 | -84.913 | 160.405 | 19.931 | 0.5873 | -52.662 | 92.524 | -11.133 | 0.9369 | -289.418 | 267.151 |
| Outbreak-time interaction β_4_ | 96.886 | 0.0001 | 50.784 | 142.989 | 37.882 | 0.0046 | 11.971 | 63.793 | 35.868 | 0.0000 | 20.533 | 51.202 | 139.189 | 0.0000 | -235.814 | -116.929 |
| Remission-time interaction β_5_ | -114.627 | 0.0000 | -161.243 | -68.011 | -49.908 | 0.0003 | -76.108 | -23.709 | -45.184 | 0.0000 | -60.689 | -29.679 | -176.371 | 0.0000 | -235.814 | -116.929 |
| Holiday β_6_ | -357.105 | 0.0000 | -437.177 | -277.032 | -193.889 | 0.0000 | -238.891 | -148.886 | -124.820 | 0.0000 | -151.453 | -98.186 | -531.032 | 0.0000 | -633.135 | -428.928 |
| **Outpatient physician visit unit (suspected)** | | | | | | | | | | | | | | | | |
| Constant β_0_ | 310.248 | 0.1716 | -136.718 | 757.215 | -5.090 | 0.8812 | -72.451 | 62.270 | -58.439 | 0.0922 | -126.637 | 9.758 | 0.363 | 0.9972 | -205.096 | 205.821 |
| Time(weeks)β_1_ | -1.026 | 0.8606 | -12.579 | 10.527 | 1.326 | 0.1341 | -0.416 | 3.067 | 1.388 | 0.1215 | -0.375 | 3.151 | 3.002 | 0.2649 | -2.309 | 8.313 |
| Seasonality terms β_ck_ and β_sk_ |  |  |  |  |  |  |  |  |  |  |  |  |  |  |  |  |
| Cos1 | 77.999 | 0.6306 | -242.715 | 398.712 | 16.856 | 0.4907 | -31.477 | 65.190 | 66.904 | 0.0078 | 17.970 | 115.838 | 104.248 | 0.1638 | -43.175 | 251.671 |
| Sin1 | -6.091 | 0.9660 | -288.574 | 276.393 | -12.976 | 0.5469 | -55.548 | 29.597 | 37.784 | 0.0851 | -5.317 | 80.884 | 15.968 | 0.8078 | -113.881 | 145.817 |
| Cos2 | 26.274 | 0.5533 | -61.341 | 113.889 | 14.673 | 0.0298 | 1.469 | 27.877 | 18.970 | 0.0059 | 5.601 | 32.884 | 35.088 | 0.0785 | -4.186 | 76.363 |
| Sin2 | -63.415 | 0.1488 | -149.882 | 23.052 | -3.488 | 0.5967 | -16.519 | 9.543 | -30.849 | 0.0000 | -44.042 | -17.656 | -61.933 | 0.0026 | -101.679 | -22.187 |
| Pandemic outbreak β_2_ | -242.793 | 0.0000 | -341.456 | -144.130 | -40.305 | 0.0000 | -55.174 | -25.436 | -42.406 | 0.0000 | -57.460 | -27.352 | -148.098 | 0.0000 | -193.451 | -102.746 |
| Pandemic remission β_3_ | 5.409 | 0.9076 | -86.792 | 97.611 | 3.328 | 0.6358 | -10.568 | 17.223 | -5.125 | 0.4716 | -19.193 | 8.943 | -4.956 | 0.8171 | -47.338 | 37.427 |
| Outbreak-time interaction β_4_ | 13.331 | 0.1776 | -6.146 | 31.808 | 1.718 | 0.2483 | -1.217 | 4.654 | 5.193 | 0.0008 | 2.221 | 8.165 | 7.965 | 0.0806 | -0.988 | 16.918 |
| Remission-time interaction β_5_ | -8.631 | 0.3868 | --28.325 | 11.063 | -2.774 | 0.0667 | -5.742 | 0.194 | -3.721 | 0.0157 | -6.725 | -0.716 | -6.407 | 0.1634 | -15.460 | 2.645 |
| Holiday β_6_ | -76.957 | 0.0000 | -110.785 | -43.129 | -11.992 | 0.0000 | -17.090 | -6.894 | -12.718 | 0.0000 | -17.879 | -7.557 | -46.317 | 0.0000 | -61.867 | -30.768 |
| **Outpatient chemotherapy unit** | | | | | | | | | | | | | | | | |
| Constant β_0_ | -17.974 | 0.7506 | -129.809 | 83.861 | 56.567 | 0.2314 | -36.615 | 149.749 | 16.396 | 0.5467 | -37.374 | 70.165 | 154.864 | 0.2135 | -90.501 | 400.228 |
| Time(weeks)β_1_ | 3.880 | 0.0090 | 0.990 | 6.771 | 1.131 | 0.3541 | -1.278 | 3.539 | 0.623 | 0.3759 | -0.767 | 2.013 | 1.441 | 0.6533 | -4.902 | 7.783 |
| Seasonality terms β_ck_ and β_sk_ |  |  |  |  |  |  |  |  |  |  |  |  |  |  |  |  |
| Cos1 | 78.949 | 0.0537 | -.1297 | 159.194 | 4.708 | 0.8892 | -62.153 | 71.569 | 13.173 | 0.4998 | -25.408 | 51.755 | -30.789 | 0.7294 | -206.847 | 145.269 |
| Sin1 | -51.395 | 0.1523 | -122.075 | 19.284 | -41.701 | 0.1632 | -100.592 | 17.190 | -6.595 | 0.7011 | -40.577 | 27.837 | -62.188 | 0.4282 | -217.258 | 92.882 |
| Cos2 | 19.113 | 0.0868 | -2.809 | 41.035 | -2.808 | 0.7611 | -21.074 | 15.458 | -1.783 | 0.7379 | -12.323 | 8.757 | -4.872 | 0.8412 | -52.968 | 43.225 |
| Sin2 | 10.637 | 0.3318 | -10.998 | 32.272 | 9.124 | 0.3178 | -8.902 | 27.151 | -0.586 | 0.9112 | -10.988 | 9.815 | 34.229 | 0.1557 | -13.237 | 81.666 |
| Pandemic outbreak β_2_ | -65.173 | 0.0000 | -89.860 | -40.487 | -53.221 | 0.0000 | -73.790 | -32.652 | -30.730 | 0.0000 | -42.599 | -18.861 | -75.903 | 0.0065 | -130.065 | -21.742 |
| Pandemic remission β_3_ | -8.268 | 0.4788 | -31.337 | 14.802 | -17.823 | 0.0688 | -37.045 | 1.398 | -0.038 | 0.9562 | -11.400 | 10.784 | -20.903 | 0.4276 | -70.940 | 30.288 |
| Outbreak-time interaction β_4_ | 5.274 | 0.0342 | 0.401 | 10.147 | 6.992 | 0.0009 | 2.932 | 11.053 | 2.460 | 0.0398 | 0.117 | 4.803 | 9.073 | 0.0954 | -1.619 | 19.765 |
| Remission-time interaction β_5_ | -10.945 | 0.0000 | -15.872 | -6.017 | -9.847 | 0.0000 | -13.952 | -5.741 | -3.244 | 0.0078 | -5.614 | -0.875 | -14.400 | 0.0095 | -25.211 | -3.589 |
| Holiday β_6_ | -27.643 | 0.0000 | -36.107 | -19.179 | -19.442 | 0.0000 | -26.495 | -12.390 | -14.315 | 0.0000 | -18.384 | -10.245 | -42.827 | 0.0000 | -61.397 | -24.257 |
| **Outpatient radiotherapy unit** | | | | | | | | | | | | | | | | |
| Constant β_0_ | 18.958 | 0.6057 | -53.644 | 91.560 | -15.067 | 0.5305 | -62.544 | 32.411 | -20.460 | 0.3396 | -62.756 | 21.835 | 95.297 | 0.0843 | -13.133 | 203.727 |
| Time(weeks)β_1_ | 0.570 | 0.5481 | -1.306 | 2.447 | 0.938 | 0.1328 | -0.290 | 2.165 | 0.894 | 0.1079 | -0.199 | 1.987 | -1.565 | 0.2707 | -4.368 | 1.238 |
| Seasonality terms β_ck_ and β_sk_ |  |  |  |  |  |  |  |  |  |  |  |  |  |  |  |  |
| Cos1 | 13.667 | 0.6040 | -38.427 | 65.761 | 22.373 | 0.1957 | -11.694 | 56.440 | 22.513 | 0.1443 | -7.835 | 52.862 | -20.040 | 0.6105 | -97.843 | 57.762 |
| Sin1 | -9.223 | 0.6910 | -55.107 | 36.661 | 6.298 | 0.6781 | -23.708 | 36.304 | 6.407 | 0.6355 | -20.324 | 33.138 | 55.362 | 0.1122 | -13.166 | 123.890 |
| Cos2 | 1.354 | 0.8507 | -12.878 | 15.585 | 9.089 | 0.0555 | -0.218 | 18.396 | 8.201 | 0.0525 | -0.090 | 16.492 | -2.098 | 0.8452 | -23.352 | 19.157 |
| Sin2 | 0.578 | 0.9351 | -13.467 | 14.623 | -2.498 | 0.5908 | -11.683 | 6.687 | -1.883 | 0.6490 | -10.065 | 6.299 | 7.607 | 0.4737 | -13.370 | 28.583 |
| Pandemic outbreak β_2_ | -11.894 | 0.1441 | -27.920 | 4.132 | -14.536 | 0.0070 | -25.016 | -4.056 | -15.931 | 0.0010 | -25.267 | -6.595 | -21.704 | 0.0750 | -45.639 | 2.230 |
| Pandemic remission β_3_ | 4.527 | 0.5501 | -10.449 | 19.504 | 3.834 | 0.4393 | -5.960 | 13.628 | 1.665 | 0.7059 | -7.060 | 10.390 | 6.649 | 0.5568 | -15.719 | 29.016 |
| Outbreak-time interaction β_4_ | 1.322 | 0.4090 | -1.841 | 4.486 | 0.126 | 0.9038 | -1.943 | 2.195 | 0.715 | 0.4434 | -1.128 | 2.558 | 4.627 | 0.0549 | -0.098 | 9.352 |
| Remission-time interaction β_5_ | -1.806 | 0.2655 | -5.005 | 1.393 | -0.635 | 0.5486 | -2.727 | 1.457 | -1.198 | 0.2053 | -3.061 | 0.666 | -1.845 | 0.4455 | -6.623 | 2.933 |
| Holiday β_6_ | -21.125 | 0.0000 | -26.620 | -15.630 | -14.418 | 0.0000 | -18.011 | -10.825 | -10.859 | 0.0000 | -14.060 | -7.658 | -43.278 | 0.0000 | -51.485 | -35.072 |
| **Pharmacy and Laboratory unit** | | | | | | | | | | | | | | | | |
| Constant β_0_ | 231.050 | 0.2295 | -148.056 | 610.156 | 144.587 | 0.4583 | -240.614 | 529.788 | 122.378 | 0.3513 | -136.816 | 381.571 | 736.713 | 0.0045 | 233.329 | 1240.096 |
| Time(weeks)β_1_ | 5.629 | 0.2573 | -4.171 | 15.428 | 1.486 | 0.7679 | -8.471 | 11.443 | -0.381 | 0.9105 | -7.080 | 6.319 | -10.771 | 0.1037 | -23.782 | 2.241 |
| Seasonality terms β_ck_ and β_sk_ |  |  |  |  |  |  |  |  |  |  |  |  |  |  |  |  |
| Cos1 | 44.316 | 0.7473 | -227.705 | 316.338 | 18.101 | 0.8969 | -258.294 | 294.496 | -10.291 | 0.9128 | -196.271 | 175.689 | -211.524 | 0.2481 | -572.718 | 149.671 |
| Sin1 | -113.888 | 0.3480 | -353.486 | 125.710 | 41.446 | 0.7363 | -201.999 | 284.891 | 50.380 | 0.5432 | -113.431 | 214.190 | 219.362 | 0.1744 | -98.775 | 537.500 |
| Cos2 | 14.008 | 0.7093 | -60.305 | 88.321 | 1.765 | 0.9631 | -73.742 | 77.273 | -10.327 | 0.6877 | -61.135 | 40.480 | -71.986 | 0.1510 | -170.660 | 26.688 |
| Sin2 | 30.888 | 0.4055 | -42.452 | 104.228 | 10.568 | 0.7791 | -63.951 | 85.086 | 11.437 | 0.6520 | -38.705 | 61.579 | 23.781 | 0.6292 | -73.600 | 121.162 |
| Pandemic outbreak β_2_ | -290.306 | 0.0000 | -373.989 | -206.623 | -33.441 | 0.4372 | -118.470 | 51.588 | -20.866 | 0.4711 | -78.080 | 36.348 | -349.369 | 0.0000 | -460.484 | -238.253 |
| Pandemic remission β_3_ | 8.320 | 0.8333 | -69.883 | 86.524 | -19.164 | 0.6334 | -98.624 | 60.297 | -0.491 | 0.9855 | -53.958 | 52.976 | -51.310 | 0.3294 | -155.149 | 52.529 |
| Outbreak-time interaction β_4_ | 19.692 | 0.0199 | 3.172 | 36.212 | -0.853 | 0.9199 | -17.639 | 15.932 | 1.831 | 0.7484 | -9.463 | 13.126 | 58.452 | 0.0000 | 36.516 | 80.387 |
| Remission-time interaction β_5_ | -31.401 | 0.0003 | -48.104 | -14.697 | -0.644 | 0.9401 | -17.617 | 16.328 | -0.720 | 0.9007 | -12.141 | 10.700 | -43.490 | 0.0002 | -65.670 | -21.311 |
| Holiday β_6_ | -99.031 | 0.0000 | -127.723 | -70.339 | -74.038 | 0.0000 | -103.191 | -44.884 | -53.115 | 0.0000 | -72.732 | -33.499 | -187.090 | 0.0000 | -225.188 | -148.992 |

Coef., coefficient

CI, confidence interval.

L 95%CI, 95%CI(Lower)

U 95%CI, 95%CI(Upper)

**eTable 9. Changes in cancer care utilization in West China Hospital Cancer Center, stratified by service type and age.**

|  | **Coef.** | **P value** | **L 95%CI** | **U 95%CI** | **Coef.** | **P** | **L 95%CI** | **U 95%CI** | **Coef.** | **P valuee** | **L 95%CI** | **U 95%CI** | **Coef.** | **P** | **L 95%CI** | **U 95%CI** | **Coef.** | **P** | **L 95%CI** | **U 95%CI** |
| --- | --- | --- | --- | --- | --- | --- | --- | --- | --- | --- | --- | --- | --- | --- | --- | --- | --- | --- | --- | --- |
|  | **≤18 years** | | | | **19-44 years** | | | | **45-59 years** | | | | **60-74 years** | | | | **≥75 years** | | | |
| **Inpatient unit (non-surgery)** | | | | | | | | | | | | | | | | | | | | |
| Constant β_0_ | 7.857 | 0.3233 | -7.842 | 23.556 | -14.166 | 0.8444 | -156.925 | 128.593 | 211.872 | 0.1862 | -103.885 | 527.630 | 71.322 | 0.5191 | -147.321 | 289.964 | 20.943 | 0.2499 | -14.951 | 56.837 |
| Time(weeks)β_1_ | 0.018 | 0.9286 | -0.387 | 0.424 | 3.444 | 0.0670 | -0.246 | 7.134 | 2.811 | 0.4961 | -5.351 | 10.972 | 3.366 | 0.2401 | -2.284 | 9.017 | -0.525 | 0.2643 | -1.453 | 0.403 |
| Seasonality terms β_ck_ and β_sk_ |  |  |  |  |  |  |  |  |  |  |  |  |  |  |  |  |  |  |  |  |
| Cos1 | -2.542 | 0.6554 | -13.807 | 8.722 | 109.104 | 0.0371 | 6.670 | 211.539 | 58.074 | 0.6123 | -168.493 | 284.641 | 74.031 | 0.3515 | -82.849 | 230.910 | -6.702 | 0.6069 | -32.457 | 19.053 |
| Sin1 | -5.028 | 03172 | -14.950 | 4.893 | 19.328 | 0.6718 | -70.896 | 109.552 | -10.808 | 0.9147 | -210.364 | 188.748 | -5.800 | 0.9338 | -143.928 | 132.328 | 19.758 | 0.0871 | -2.927 | 42.443 |
| Cos2 | -0.074 | 0.9621 | -3.151 | 3.003 | 28.696 | 0.0445 | 0.713 | 56.680 | 18.932 | 0.5454 | -42.964 | 80.827 | 30.953 | 0.1551 | -11.907 | 73.814 | -0.413 | 0.9076 | -7.449 | 6.623 |
| Sin2 | 1.221 | 0.4270 | -1.816 | 4.258 | -10.062 | 0.4716 | -37.679 | 17.556 | 14.774 | 0.6325 | -46.309 | 75.858 | -0.007 | 0.9997 | -42.315 | 42.300 | 0.581 | 0.8686 | -6.363 | 7.525 |
| Pandemic outbreak β_2_ | -2.702 | 0.1250 | -6.168 | 0.763 | -119.926 | 0.0000 | -151.439 | -88.414 | -263.724 | 0.0000 | -333.424 | -194.024 | -183.763 | 0.0000 | -232.016 | -135.511 | -14.306 | 0.0005 | -22.229 | -6.383 |
| Pandemic remission β_3_ | 2.357 | 0.1520 | -0.882 | 5.595 | 23.915 | 0.1103 | -5.534 | 53.364 | 60.478 | 0.0684 | -4.657 | 125.613 | 21.210 | 0.3528 | -23.859 | 66.279 | 0.594 | 0.8739 | -6.810 | 7.998 |
| Outbreak-time interaction β_4_ | 0.032 | 0.9267 | -0.652 | 0.716 | 7.790 | 0.0146 | 1.569 | 14.010 | 19.909 | 0.0050 | 6.150 | 33.669 | 13.331 | 0.0065 | 3.806 | 22.855 | 2.938 | 0.0003 | 1.374 | 4.502 |
| Remission-time interaction β_5_ | -0.280 | 0.4245 | -0.971 | 0.412 | -10.446 | 0.0014 | -16.736 | -4.155 | -23.622 | 0.0011 | -37.535 | -9.170 | -16.766 | 0.0008 | -26.397 | -7.135 | -1.920 | 0.0178 | -3.501 | -0.338 |
| Holiday β_6_ | -1.999 | 0.0012 | -3.187 | -0.811 | -48.924 | 0.0000 | -59.729 | -38.120 | -119.083 | 0.0000 | -142.980 | -95.185 | -80.285 | 0.0000 | -96.829 | -63.742 | -7.853 | 0.0000 | -10.570 | -5.137 |
| **Inpatient unit (surgery)** | | | | | | | | | | | | | | | | | | | | |
| Constant β_0_ | -1.773 | 0.9633 | -77.885 | 74.340 | 33.233 | 0.7544 | -176.866 | 243.331 | 43.942 | 0.7997 | -298.700 | 386.584 | 10.143 | 0.9397 | -254.924 | 275.210 | 1.143 | 0.9763 | -75.104 | 77.389 |
| Time(weeks)β_1_ | 0.674 | 0.4981 | -1.293 | 2.642 | 1.368 | 0.6185 | -4.063 | 6.799 | 2.875 | 0.5212 | -5.982 | 11.731 | 2.483 | 0.4739 | -4.368 | 9.335 | 0.605 | 0.5440 | -1.366 | 2.576 |
| Seasonality terms β_ck_ and β_sk_ |  |  |  |  |  |  |  |  |  |  |  |  |  |  |  |  |  |  |  |  |
| Cos1 | 34.798 | 0.2091 | -19.815 | 89.411 | 52.663 | 0.4900 | -98.090 | 203.416 | 81.577 | 0.5120 | -164.280 | 327.435 | 73.793 | 0.4433 | -116.398 | 263.991 | 17.365 | 0.5304 | -37.344 | 72.074 |
| Sin1 | 14.265 | 0.5577 | -33.838 | 62.368 | 79.884 | 0.2355 | -52.898 | 212.666 | 99.794 | 0.3629 | -116.754 | 316.343 | 87.731 | 0.3014 | -79.793 | 255.255 | 9.926 | 0.6837 | -38.261 | 58.113 |
| Cos2 | 4.266 | 0.5719 | -10.654 | 19.186 | 34.361 | 0.1010 | -6.823 | 75.545 | 39.490 | 0.2463 | -27.675 | 106.655 | 39.457 | 0.1351 | -12.502 | 91.416 | 3.539 | 0.6396 | -11.701 | 18.485 |
| Sin2 | -7.503 | 0.3146 | -22.227 | 7.222 | -19.931 | 0.3330 | -60.576 | 20.713 | -10.115 | 0.7628 | -76.400 | 56.171 | -11.606 | 0.6545 | -62.885 | 39.672 | 0.049 | 0.9948 | -14.701 | 14.798 |
| Pandemic outbreak β2 | -31.938 | 0.0003 | -48.739 | -15.138 | -166.348 | 0.0000 | -212.725 | -119.971 | -274.914 | 0.0000 | -350.548 | -199.280 | -197.030 | 0.0000 | -255.542 | -138.519 | -42.856 | 0.0000 | -59.688 | -26.025 |
| Pandemic remission β_3_ | 15.773 | 0.0490 | 0.072 | 31.473 | 17.967 | 0.4129 | -25.373 | 61.306 | 30.963 | 0.3870 | -39.718 | 101.644 | 24.700 | 0.3724 | -29.980 | 79.380 | 11.946 | 0.1350 | -3.783 | 27.675 |
| Outbreak-time interaction β_4_ | 2.265 | 0.1785 | -1.051 | 5.582 | 14.888 | 0.0017 | 5.733 | 24.043 | 28.623 | 0.0002 | 13.692 | 43.553 | 18.440 | 0.0020 | 6.889 | 29.990 | 4.823 | 0.0049 | 1.501 | 8.146 |
| Remission-time interaction β_5_ | -1.950 | 0.2514 | -5.304 | 1.403 | -11.864 | 0.0125 | -21.121 | -2.067 | -26.181 | 0.0008 | -41.278 | -11.083 | -17.064 | 0.0046 | -28.743 | -5.384 | -5.049 | 0.0036 | -8.049 | -1.690 |
| Holiday β_6_ | -16.114 | 0.0000 | -21.875 | -10.354 | -67.123 | 0.0000 | -83.024 | -51.222 | -113.293 | 0.0000 | -139.225 | -87.361 | -83.956 | 0.0000 | -104.017 | -63.894 | -15.863 | 0.0000 | -21.634 | -10.093 |
| **Outpatient physician visit unit (diagnosed)** | | | | | | | | | | | | | | | | | | | | |
| Constant β_0_ | -1.874 | 0.9578 | -71.861 | 68.114 | 180.409 | 0.6061 | -511.369 | 872.188 | 430.974 | 0.5436 | -971.592 | 1833.540 | 567.509 | 0.2572 | -420.454 | 1555.471 | -0.911 | 0.9934 | -218.229 | 216.408 |
| Time(weeks)β_1_ | -0.114 | 0.9006 | -1.923 | 1.695 | 19.927 | 0.0293 | 2.046 | 37.808 | 37.320 | 0.0437 | 1.066 | 73.574 | 14.382 | 0.2666 | -11.155 | 39.919 | 4.979 | 0.0817 | -0.638 | 10.596 |
| Seasonality terms β_ck_ and β_sk_ |  |  |  |  |  |  |  |  |  |  |  |  |  |  |  |  |  |  |  |  |
| Cos1 | 34.248 | 0.1792 | -15.970 | 84.466 | 439.943 | 0.0818 | -56.413 | 936.317 | 738.005 | 0.1489 | -268.384 | 1744.393 | 262.606 | 0.4642 | -446.288 | 971.504 | 124.359 | 0.1168 | -31.567 | 280.286 |
| Sin1 | 59.633 | 0.0087 | 15.432 | 103.895 | -143.816 | 0.5156 | -581.023 | 293.391 | -280.948 | 0.5310 | -1167.355 | 605.464 | 52.001 | 0.8691 | -572.380 | 676.383 | 43.816 | 0.5284 | -93.539 | 181.171 |
| Cos2 | 9.746 | 0.1619 | -3.973 | 23.465 | 114.854 | 0.0960 | -20.750 | 250.457 | 170.953 | 0.2203 | -103.981 | 445.886 | 88.894 | 0.3648 | -104.768 | 282.556 | 41.074 | 0.0587 | -1.531 | 83.678 |
| Sin2 | -15.450 | 0.0257 | -28.989 | -1.911 | -27.049 | 0.6894 | -160.977 | 106.778 | -28.752 | 0.8340 | -300.081 | 242.578 | -34.776 | 0.7189 | -225.897 | 156.345 | -19.611 | 0.3571 | -61.653 | 22.432 |
| Pandemic outbreak β_2_ | -33.415 | 0.0000 | -1.923 | 1.695 | -811.565 | 0.0000 | -964.268 | -658.862 | -1608.510 | 0.0000 | -1918.108 | -1298.913 | -1042.369 | 0.0000 | -1260.451 | -824.288 | -191.674 | 0.0000 | -239.647 | -143.701 |
| Pandemic remission β_3_ | 2.686 | 0.7129 | -11.751 | 17.123 | 9.563 | 0.8945 | -133.138 | 152.264 | 4.744 | 0.9741 | -284.579 | 294.067 | 6.429 | 0.9502 | -197.368 | 210.227 | 15.114 | 0.5052 | -29.717 | 59.946 |
| Outbreak-time interaction β_4_ | 5.315 | 0.0008 | 2.265 | 8.364 | 64.428 | 0.0000 | 34.283 | 94.573 | 139.178 | 0.0000 | 78.061 | 200.795 | 90.134 | 0.0001 | 47.083 | 133.185 | 10.771 | 0.0262 | 1.301 | 20.241 |
| Remission-time interaction β_5_ | -2.744 | 0.0806 | 2.265 | 0.340 | -87.843 | 0.0000 | -118.324 | -57.362 | -181.593 | 0.0000 | -243.391 | -119.795 | -100.801 | 0.0000 | -144.332 | -57.271 | -13.110 | 0.0078 | -22.686 | -3.534 |
| Holiday β_6_ | -15.566 | 0.0000 | -20.863 | -10.269 | -248.398 | 0.0000 | -300.754 | -196.041 | -515.508 | 0.0000 | -621.658 | -409.357 | -355.089 | 0.0000 | -429.861 | -280.316 | -72.285 | 0.0000 | -88.733 | -55.836 |
| **Outpatient physician visit unit (suspected)** | | | | | | | | | | | | | | | | | | | | |
| Constant β_0_ | 24.525 | 0.2699 | -19.321 | 68.370 | 250.051 | 0.1038 | -52.086 | 552.188 | 28.324 | 0.8348 | -240.408 | 297.055 | -66.098 | 0.2854 | -188.182 | 55.987 | 10.275 | 0.6219 | -30.923 | 51.472 |
| Time(weeks)β_1_ | -0.614 | 0.2851 | -1.747 | 0.519 | 0.187 | 0.9623 | -7.623 | 7.996 | 2.823 | 0.4221 | -4.123 | 9.769 | 2.808 | 0.0806 | -0.348 | 5.963 | -0.514 | 0.3408 | -1.579 | 0.551 |
| Seasonality terms β_ck_ and β_sk_ |  |  |  |  |  |  |  |  |  |  |  |  |  |  |  |  |  |  |  |  |
| Cos1 | -6.400 | 0.6874 | -37.861 | 25.069 | 30.257 | 0.7825 | -186.536 | 247.050 | 123.838 | 0.2056 | -68.986 | 316.661 | 111.461 | 0.0131 | 23.861 | 199.060 | 6.855 | 0.6465 | -22.705 | 36.416 |
| Sin1 | 11.462 | 0.4139 | -16.248 | 39.172 | -73.935 | 0.4443 | -264.883 | 117.013 | 27.872 | 0.7455 | -141.969 | 197.713 | 36.902 | 0.3451 | -40.254 | 114.058 | 32.386 | 0.00153 | 6.349 | 58.422 |
| Cos2 | 0.329 | 0.9396 | -8.266 | 8.924 | 8.786 | 0.7692 | -50.439 | 68.011 | 44.905 | 0.0939 | -7.772 | 97.582 | 37.450 | 0.0025 | 13.519 | 61.381 | 4.536 | 0.2678 | -3.539 | 12.612 |
| Sin2 | -6.501 | 0.1315 | -14.983 | 1.981 | -67.135 | 0.0248 | -125.584 | -8.686 | -53.161 | 0.0451 | -105.148 | -1.173 | -24.145 | 0.0452 | -47.762 | -0.528 | -8.734 | 0.0319 | -16.713 | -0.773 |
| Pandemic outbreak β_2_ | -7.697 | 0.1178 | -17.375 | 1.981 | -215.880 | 0.0000 | -282.573 | -149.188 | -167.559 | 0.0000 | -226.878 | -108.240 | -68.624 | 0.0000 | -95.573 | -41.676 | -13.841 | 0.0032 | -22.935 | -4.747 |
| Pandemic remission β_3_ | -3.809 | 0.4055 | -12.854 | 5.235 | 4.807 | 0.8787 | -57.518 | 67.133 | 0.851 | 0.9758 | -54.584 | 56.287 | -1.046 | 0.9345 | -26.230 | 24.138 | -2.148 | 0.6173 | -10.646 | 6.351 |
| Outbreak-time interaction β_4_ | 0.921 | 0.3415 | -0.990 | 2.831 | 9.833 | 0.1416 | -3.333 | 22.998 | 10.661 | 0.0739 | -1.049 | 22.371 | 4.380 | 0.1056 | -0.940 | 9.700 | 2.414 | 0.0089 | 0.619 | 4.209 |
| Remission-time interaction β_5_ | 0.393 | 0.6871 | -1.538 | 2.325 | -7.713 | 0.2532 | -21.025 | 5.599 | -9.346 | 0.1205 | -21.187 | 2.494 | -4.440 | 0.1047 | -9.820 | 0.939 | -0.426 | 0.6423 | -2.242 | 1.389 |
| Holiday β_6_ | -2.276 | 0.1767 | -5.595 | 1.0422 | -60.406 | 0.0000 | -83.272 | -37.539 | -52.403 | 0.0000 | -72.742 | -32.065 | -27.095 | 0.0000 | -36.335 | -17.855 | -5.084 | 0.0004 | -9.922 | -2.686 |
| **Outpatient chemotherapy unit** | | | | | | | | | | | | | | | | | | | | |
| Constant β_0_ | -3.354 | 0.0263 | -6.305 | -0.403 | 87.571 | 0.1203 | -23.306 | 198.447 | -21.291 | 0.8586 | -257.692 | 215.110 | 35.818 | 0.5029 | -69.845 | 141.482 | -6.494 | 0.5800 | -29.695 | 16.708 |
| Time(weeks)β_1_ | -0.003 | 0.9645 | -0.079 | 0.074 | 0.394 | 0.7865 | -2.472 | 3.260 | 5.003 | 0.1074 | -1.107 | 11.114 | 1.547 | 0.2638 | -1.184 | 4.279 | 0.723 | 0.0187 | 0.123 | 1.322 |
| Seasonality terms β_ck_ and β_sk_ |  |  |  |  |  |  |  |  |  |  |  |  |  |  |  |  |  |  |  |  |
| Cos1 | 2.713 | 0.0126 | 0.595 | 4.830 | -20.075 | 0.6178 | -99.633 | 59.483 | 127.822 | 0.1381 | -41.804 | 297.448 | 23.469 | 0.5406 | -52.348 | 99.286 | 14.401 | 0.0892 | -2.246 | 31.049 |
| Sin1 | 4.230 | 0.0000 | 2.365 | 6.095 | -37.829 | 0.26868 | -107.903 | 32.245 | 18.114 | 0.8105 | -131.291 | 167.519 | -7.826 | 0.8167 | -74.606 | 58.953 | -5.024 | 0.4983 | -19.688 | 9.639 |
| Cos2 | 0.528 | 0.0733 | -0.051 | 1.106 | -4.389 | 0.6896 | -26.123 | 17.345 | 35.832 | 0.1282 | -10.508 | 82.171 | 6.829 | 0.5146 | -13.883 | 27.542 | 2.872 | 0.2133 | -1.676 | 7.420 |
| Sin2 | -1.507 | 0.0000 | -2.078 | -0.936 | 17.111 | 0.1167 | -4.339 | 38.560 | -13.916 | 0.5475 | -59.649 | 31.816 | 8.815 | 0.3944 | -11.626 | 29.256 | -0.944 | 0.6774 | -5.433 | 3.544 |
| Pandemic outbreak β_2_ | 0.118 | 0.7207 | -0.534 | 0.769 | -39.319 | 0.0019 | -63.794 | -14.845 | -124.089 | 0.0000 | -176.271 | -71.906 | -62.666 | 0.0000 | -85.990 | -39.342 | -15.001 | 0.0000 | -20.122 | -9.880 |
| Pandemic remission β_3_ | -0.710 | 0.0228 | -1.318 | -0.101 | -5.490 | 0.6350 | -28.362 | 17.382 | -9.687 | 0.6944 | -58.452 | 39.078 | -5.962 | 0.5887 | -27.759 | 15.834 | 5.214 | 0.0330 | 0.428 | 10.000 |
| Outbreak-time interaction β_4_ | 0.075 | 0.2523 | -0.054 | 0.203 | 3.684 | 0.1335 | -1.147 | 8.516 | 6.497 | 0.2138 | -3.804 | 16.798 | 4.534 | 0.0536 | -0.071 | 9.138 | 0.053 | 0.9179 | -0.958 | 1.064 |
| Remission-time interaction β_5_ | 0.136 | 0.0412 | 0.005 | 0.266 | -5.876 | 0.0189 | -10.761 | -0.990 | -9.976 | 0.0603 | -20.392 | 0.440 | -6.555 | 0.0062 | -11.210 | -1.899 | -0.920 | 0.0774 | -1.942 | 0.103 |
| Holiday β_6_ | -0.038 | 0.7353 | -0.262 | 0.185 | -20.548 | 0.0000 | -28.939 | -12.156 | -50.073 | 0.0000 | -67.964 | -32.181 | -23.855 | 0.0000 | -31.852 | -15.858 | -3.663 | 0.0001 | -5.419 | -1.908 |
| **Outpatient radiotherapy unit** | | | | | | | | | | | | | | | | | | | | |
| Constant β_0_ | 5.729 | 0.5572 | -13.562 | 25.020 | 33.517 | 0.1740 | -15.036 | 82.070 | -38.876 | 0.4714 | -145.534 | 67.783 | 78.934 | 0.0359 | 5.281 | 152.588 | -0.578 | 0.9708 | -31.824 | 30.668 |
| Time(weeks)β_1_ | -0.108 | 0.6684 | -0.607 | 0.391 | -0.490 | 0.4403 | -1.745 | 0.765 | 2.203 | 0.11160 | -0.553 | 4.960 | -0.826 | 0.3916 | -2.730 | 1.078 | 0.058 | 0.8877 | -0.750 | 0.865 |
| Seasonality terms β_ck_ and β_sk_ |  |  |  |  |  |  |  |  |  |  |  |  |  |  |  |  |  |  |  |  |
| Cos1 | -2.501 | 0.7208 | -16.343 | 11.341 | -7.421 | 0.6736 | -42.259 | 27.417 | 74.429 | 0.0565 | -2.102 | 150.960 | -29.242 | 0.2750 | -82.091 | 23.607 | 3.249 | 0.7744 | -19.171 | 25.669 |
| Sin1 | 3.296 | 0.5930 | -8.895 | 15.488 | 13.206 | 0.3953 | -17.479 | 43.892 | 25.829 | 0.4490 | -41.580 | 93.237 | 3.875 | 0.8962 | -42.673 | 50.424 | 12.637 | 0.2072 | -7.110 | 32.384 |
| Cos2 | -0.041 | 0.9828 | -3.823 | 3.740 | -1.331 | 0.7820 | -10.849 | 8.186 | 20.757 | 0.0516 | -0.150 | 41.664 | -5.745 | 0.4319 | -20.182 | 8.693 | 2.906 | 0.3489 | -3.219 | 9.031 |
| Sin2 | 0.971 | 0.6071 | -2.761 | 4.703 | -1.878 | 0.6925 | -11.271 | 7.515 | -4.429 | 0.6712 | -25.062 | 16.205 | 8.667 | 0.2304 | -5.581 | 22.916 | 0.472 | 0.8771 | -5.572 | 6.571 |
| Pandemic outbreak β_2_ | -0.885 | 0.6811 | -5.143 | 3.373 | -10.730 | 0.0497 | -21.447 | -0.012 | -27.976 | 0.0203 | -51.520 | -4.433 | -20.290 | 0.0149 | -36.549 | -4.032 | -4.183 | 0.2318 | -11.080 | 2.714 |
| Pandemic remission β_3_ | 1.604 | 0.4258 | -2.375 | 5.584 | -1.030 | 0.8388 | -11.046 | 8.985 | 4.054 | 0.7155 | -17.948 | 26.056 | 10.117 | 0.1896 | -5.077 | 25.310 | 1.930 | 0.5539 | -4.516 | 8.376 |
| Outbreak-time interaction β_4_ | 0.565 | 0.1856 | -0.276 | 1.405 | 1.895 | 0.0786 | -0.220 | 4.011 | 1.850 | 0.4316 | -2.797 | 6.498 | 2.381 | 0.1443 | -0.829 | 5.590 | 0.099 | 0.8851 | -1.262 | 1.461 |
| Remission-time interaction β_5_ | -0.482 | 0.2631 | -1.332 | 0.368 | -0.717 | 0.5077 | -2.856 | 1.422 | -2.709 | 0.2556 | -7.408 | 1.991 | -1.783 | 0.2785 | -5.028 | 1.463 | 0.207 | 0.7656 | -1.169 | 1.584 |
| Holiday β_6_ | -2.126 | 0.0047 | -3.586 | -0.666 | -14.064 | 0.0000 | -17.739 | -10.389 | -38.809 | 0.0000 | -46.882 | -30.737 | -28.725 | 0.0000 | -34.299 | -23.150 | -5.596 | 0.0000 | -8.321 | -3.591 |
| **Pharmacy and Laboratory unit** | | | | | | | | | | | | | | | | | | | | |
| Constant β_0_ | 1.344 | 0.7265 | -6.255 | 8.943 | 326.282 | 0.0532 | -4.560 | 657.125 | 791.270 | 0.0460 | 14.325 | 1568.215 | 468.902 | 0.0084 | 122.872 | 814.933 | 86.442 | 0.0429 | 2.834 | 170.050 |
| Time(weeks)β_1_ | -0.069 | 0.4888 | -0.265 | 0.128 | -4.650 | 0.2834 | -13.201 | 3.902 | -5.178 | 0.6102 | -25.260 | 14.905 | -8.091 | 0.0757 | -17.036 | 0.853 | 0.192 | 0.8608 | -1.970 | 2.353 |
| Seasonality terms β_ck_ and β_sk_ |  |  |  |  |  |  |  |  |  |  |  |  |  |  |  |  |  |  |  |  |
| Cos1 | -0.488 | 0.8594 | -5.941 | 4.964 | -70.638 | 0.5564 | -308.028 | 166.752 | -232.705 | 0.4097 | -790.189 | 324.780 | -149.088 | 0.2364 | -397.376 | 99.021 | -9.908 | 0.7439 | -69.900 | 50.083 |
| Sin1 | 2.979 | 0.2215 | -1.824 | 7.782 | 105.552 | 0.3191 | -103.542 | 314.646 | 60.667 | 0.8069 | -430.359 | 551.693 | 161.753 | 0.1454 | -56.938 | 380.445 | -20.158 | 0.4510 | -72.998 | 32.683 |
| Cos2 | -0.209 | 0.7812 | -1.699 | 1.280 | -38.618 | 0.2403 | -103.471 | 26.234 | -68.196 | 0.3766 | -220.494 | 84.102 | -57.711 | 0.0946 | -125.541 | 10.119 | -5.165 | 0.5333 | -21.554 | 11.224 |
| Sin2 | -0.696 | 0.3498 | -2.166 | 0.774 | 17.443 | 0.5900 | -45.560 | 81.445 | 69.136 | 0.3638 | -81.166 | 219.438 | 36.852 | 0.2775 | -30.089 | 103.793 | 5.876 | 0.4278 | -10.298 | 22.050 |
| Pandemic outbreak β_2_ | -1.778 | 0.0380 | -3.455 | -0.100 | -125.945 | 0.0009 | -198.974 | -52.915 | -153.906 | 0.0781 | -325.407 | 17.596 | -238.114 | 0.0000 | -314.496 | -161.732 | -26.447 | 0.0054 | -44.903 | -7.991 |
| Pandemic remission β_3_ | -0.466 | 0.5568 | -2.034 | 1.102 | 2.808 | 0.9351 | -65.440 | 71.055 | 11.459 | 0.8875 | -148.811 | 171.729 | -23.021 | 0.5238 | -94.401 | 48.360 | 2.496 | 0.7747 | -14.751 | 19.743 |
| Outbreak-time interaction β_4_ | 0.287 | 0.0892 | -0.045 | 0.618 | 23.299 | 0.0018 | 8.882 | 37.716 | 11.219 | 0.5125 | -22.637 | 45.074 | 43.555 | 0.0000 | 28.477 | 58.634 | 0.925 | 0.6156 | -2.718 | 4.568 |
| Remission-time interaction β_5_ | -0.101 | 0.5515 | -0.436 | 0.234 | -16.927 | 0.0233 | -31.504 | -2.350 | -8.914 | 0.6067 | -43.147 | 25.319 | -33.226 | 0.0000 | -48.472 | -17.979 | -2.231 | 0.2325 | -5.915 | 1.453 |
| Holiday β_6_ | -0.380 | 0.1930 | -0.955 | 0.195 | -85.875 | 0.0000 | -110.914 | -60.836 | -184.173 | 0.0000 | -242.974 | -125.371 | -125.383 | 0.0000 | -151.572 | -99.194 | -12.441 | 0.0002 | -18.769 | -6.113 |
| **Telemedicine (diagnosed)** | | | | | | | | | | | | | | | | | | | | |
| Constant β_0_ | 0.431 | 0.9627 | -17.773 | 18.635 | 26.592 | 0.8357 | -227.001 | 280.184 | -119.680 | 0.3942 | -397.083 | 157.724 | -132.818 | 0.1969 | -335.636 | 70.000 | -23.658 | 0.4437 | -84.679 | 37.364 |
| Time(weeks)β_1_ | -0.106 | 0.6568 | -0.576 | 0.365 | -4.748 | 0.1529 | -22.303 | 1.807 | -2.988 | 0.4015 | -10.158 | 4.183 | -1.927 | 0.4677 | -7.169 | 3.316 | -0.166 | 0.8351 | -1.734 | 1.411 |
| Seasonality terms β_ck_ and β_sk_ |  |  |  |  |  |  |  |  |  |  |  |  |  |  |  |  |  |  |  |  |
| Cos1 | 0.153 | 0.9815 | -12.909 | 13.215 | -19.814 | 0.8294 | -201.776 | 162.147 | 90.015 | 0.3719 | -109.032 | 289.062 | 107.124 | 0.1474 | -38.405 | 252.653 | 24.062 | 0.2783 | -19.723 | 67.847 |
| Sin1 | 5.073 | 0.3839 | -6.432 | 16.578 | 232.646 | 0.0049 | 72.376 | 392.916 | 305.951 | 0.0008 | 130.632 | 481.270 | 257.269 | 0.0001 | 129.087 | 385.451 | 34.033 | 0.0831 | -4.533 | 72.598 |
| Cos2 | 0.028 | 0.9875 | -3.540 | 3.597 | 6.343 | 0.8007 | -43.366 | 56.053 | 29.598 | 0.2829 | -24.779 | 83.975 | 23.607 | 0.2417 | -16.150 | -33.539 | -0.405 | 0.9466 | -12.366 | 11.557 |
| Sin2 | -0.850 | 0.6331 | -4.372 | 2.672 | -34.962 | 0.1605 | -84.021 | 14.096 | -73.634 | 0.0076 | -127.299 | -19.969 | -72.775 | 0.0004 | -112.011 | -33.539 | -11.471 | 0.0567 | -23.276 | 0.333 |
| Pandemic outbreak β_2_ | -1.219 | 0.5486 | -5.238 | 2.799 | -36.207 | 0.2024 | -92.184 | 19.771 | 26.243 | 0.3973 | -34.9990 | 87.477 | 49.399 | 0.8890 | 4.629 | 94.169 | 24.029 | 0.0006 | 10.560 | 37.499 |
| Pandemic remission β_3_ | 1.088 | 0.5667 | -2.667 | 4.843 | 50.611 | 0.0578 | -1.701 | 102.923 | 7.371 | 0.7989 | -49.853 | 64.595 | 2.951 | 0.8890 | -38.887 | 44.789 | -11.596 | 0.0706 | -24.183 | 0.992 |
| Outbreak-time interaction β_4_ | 0.833 | 0.0397 | 0.040 | 1.626 | 31.010 | 0.0000 | 19.959 | 42.060 | 31.130 | 0.0000 | 19.042 | 43.218 | 18.153 | 0.0001 | 9.315 | 26.991 | 3.328 | 0.0147 | 0.669 | 5.987 |
| Remission-time interaction β_5_ | -0.549 | 0.1775 | -1.351 | 0.253 | -16.510 | 0.0042 | --27.683 | -5.336 | -13.098 | 0.0359 | -25.321 | -0.876 | -3.720 | 0.4110 | -12.656 | 5.217 | -1.283 | 0.3463 | -3.971 | 1.406 |
| Holiday β_6_ | -0.849 | 0.2246 | -2.226 | 0.529 | -30.117 | 0.0024 | -49.310 | -10.924 | -22.290 | 0.0377 | -43.285 | -1.295 | -12.043 | 0.1228 | -27.393 | 3.307 | -4.736 | 0.0446 | -9.354 | -0.117 |
| **Telemedicine (suspected)** | | | | | | | | | | | | | | | | | | | | |
| Constant β_0_ | 11.720 | 0.3132 | -11.213 | 34.653 | 259.623 | 0.2828 | -217.269 | 736.515 | -102.752 | 0.5670 | -457.579 | 252.075 | -11.275 | 0.8832 | -163.128 | 140.579 | -25.101 | 0.0224 | -46.581 | -3.621 |
| Time(weeks)β_1_ | -0.572 | 0.0583 | -1.165 | 0.021 | -8.894 | 0.1555 | -21.221 | 3.433 | -3.303 | 0.4766 | -12.475 | 5.868 | 0.240 | 0.9438 | -3.785 | 4.065 | 0.001 | 0.9961 | -0.554 | 0.557 |
| Seasonality terms β_ck_ and β_sk_ |  |  |  |  |  |  |  |  |  |  |  |  |  |  |  |  |  |  |  |  |
| Cos1 | -9.048 | 0.2780 | -25.504 | 7.407 | -193.480 | 0.2647 | -535.666 | 148.706 | 83.673 | 0.5160 | -170.927 | 338.273 | 11.351 | 0.8367 | -97.609 | 120.310 | 20.440 | 0.0098 | 5.027 | 35.853 |
| Sin1 | 17.442 | 0.0188 | 2.949 | 31.936 | 210.040 | 0.1699 | -91.354 | 511.434 | 319.006 | 0.0057 | 94.756 | 543.257 | 15.556 | 0.7485 | -80.417 | 111.530 | 29.424 | 0.0000 | 15.851 | 42.997 |
| Cos2 | 0.656 | 0.7728 | -3.839 | 5.152 | -29.489 | 0.5329 | -122.971 | 63.992 | 24.898 | 0.4794 | -44.656 | 94.452 | 0.130 | 0.9931 | -29.636 | 29.897 | 3.638 | 0.0896 | -0.572 | 7.849 |
| Sin2 | -1.665 | 0.4584 | -6.102 | 2.771 | 10.460 | 0.8225 | -81.796 | 102.716 | -74.192 | 0.0344 | -142.835 | -5.550 | -0.718 | 0.9614 | -30.094 | 28.659 | -9.867 | 0.0000 | -14.022 | -5.712 |
| Pandemic outbreak β_2_ | -0.967 | 0.7055 | -6.029 | 4.095 | -109.890 | 0.0409 | -215.158 | -4.621 | -62.831 | 0.1147 | -141.155 | 15.493 | -14.701 | 0.3864 | -48.222 | 18.820 | -0.028 | 0.9906 | -4.769 | 4.713 |
| Pandemic remission β_3_ | 2.620 | 0.2745 | -2.110 | 7.351 | 129.847 | 0.0102 | 31.473 | 228.222 | 66.264 | 0.0755 | -6.931 | 139.459 | 34.681 | 0.0304 | 3.355 | 66.007 | -2.387 | 0.2879 | -6.817 | 2.044 |
| Outbreak-time interaction β_4_ | 0.903 | 0.0760 | -0.096 | 1.902 | 40.932 | 0.0002 | 20.151 | 61.713 | 28.458 | 0.0004 | 12.996 | 43.920 | 6.470 | 0.0522 | -0.148 | 13.087 | 1.338 | 0.0055 | 0.402 | 2.274 |
| Remission-time interaction β_5_ | 0.391 | 0.4445 | -0.619 | 1.402 | -20.638 | 0.0541 | -41.650 | 0.375 | -7.285 | 0.3576 | -22.919 | 8.349 | -4.762 | 0.1612 | -11.453 | 1.929 | 0.226 | 0.6371 | -0.721 | 1.172 |
| Holiday β_6_ | -1.439 | 0.1032 | -3.174 | 0.297 | -68.620 | 0.0003 | -104.712 | -32.527 | -37.424 | 0.0068 | -64.279 | -10.570 | -13.838 | 0.0188 | -25.331 | -2.344 | 0.180 | 0.8267 | -1.446 | 1.805 |

Coef., coefficient

P, P value

CI, confidence interval.

L, 95%CI(Lower)

U, 95%CI(Upper)
